# Supplementary material for: Target-Cell-Directed Bioengineering Approaches for Gene Therapy of Hemophilia A
Source: Mol Ther Methods Clin Dev. 2018 Jan 31;9:57–69. doi: 10.1016/j.omtm.2018.01.004 (PMC5852392; doi:10.1016/j.omtm.2018.01.004)
Supplement: Document S1. Supplemental Materials and Methods and Figures S1 and S2 [file mmc1.pdf]

OMTM, Volume *9*

Supplemental Information

Target-Cell-Directed Bioengineering Approaches for Gene Therapy of Hemophilia A

Harrison C. Brown, Philip M. Zakas, Stephan N. George, Ernest T. Parker, H. Trent Spencer, and Christopher B. Doering

Supplementary Figure 1

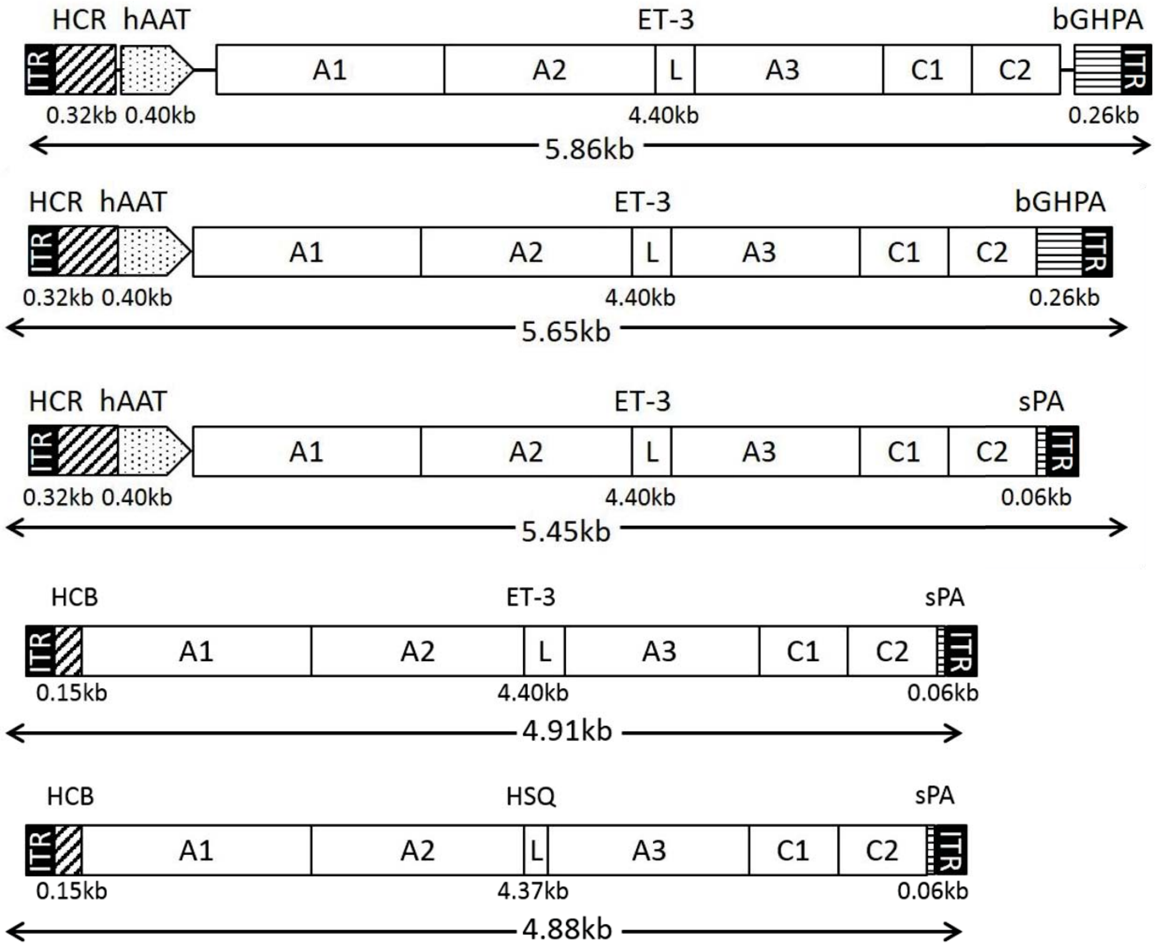

AAV transgene designs: To-scale vector schematics show the discrete elements and size of the resulting AAV transgene designs.

Promoter Sequences

The promoters were synthesized *de novo* and cloned into an expression plasmid driving the expression of coagulation factor VIII. Factor VIII activity was measured 48 hours after transfection by one-stage clot assay. As a comparator, the hybrid liver promoter (HLP) is used in this and other experiments. HLP represents one of the shortest yet most powerful liver-directed promoters described to date.

HLP reference: Therapeutic levels of FVIII following a single peripheral vein administration of rAAV vector encoding a novel human factor VIII variant. McIntosh J. Blood. 2013 Apr 25;121(17):3335-44. doi: 10.1182/blood-2012-10-462200. Epub 2013 Feb 20.

Native Sequence Elements

ABP element
5’GTTAATTTTTAAAAAGCAGTCAAAAGTCCAAGTGGCCCTTGCGAGCATTTACTCTCTCTGTTTGCTCTGGTTAATAATCTCAGGAGCACAAACA 3’

GTTA HNF-1-1 transcription factor binding site
HNF-4 transcription factor binding site
HNF-3a transcription factor binding site
HNF1-2 transcription factor binding site
HNF-3-2 transcription factor binding site

Reference: A potent enhancer made of clustered liver-specific elements in the transcription control sequences of human alpha 1-microglobulin/bikunin gene. Rouet P. J Biol Chem. 1992 Oct 15;267(29):20765-73.

HP1 element
5’GTTAATAATTTTC3’

Reference: Hepatocyte-specific promoter element HP1 of the Xenopus albumin gene interacts with transcriptional factors of mammalian hepatocytes. Schorpp M. J Mol Biol. 1988 Jul 20;202(2):307-20.

AFP element
5’AGTCATATGTTTGCTCACTGAAGGTTACTAGTTAACAGGCATCCCTTAAACAGGA3’

Reference: Multiple regulatory elements in the intergenic region between the alpha-fetoprotein and albumin genes. Godbout R, Mol Cell Biol. 1986 Feb;6(2):477-87.

SynO element
5’GAGGTTAATAATTTTCCAGATCTCTCTGAGCAATAGTATAA3’

HP1 binding element

Reference: Liver cell specific gene transcription in vitro: the promoter elements HP1 and TATA box are necessary and sufficient to generate a liver-specific promoter. G U Ryffel Nucleic Acids Res. 1989 Feb 11; 17(3): 939–953.

HNF1a transcription factor binding site (liver-directed transcription factor)
5’GTTAATCATTAA3’

SP1 binding site (liver-directed transcription factor)
5’TGGGCGGAGT3’

Modifications to native sequences

Transcription Start Site
5’GCCAGCAGCAGCCTGACCACATCTCATCCTC3’

CATC GC rich spacer
Transcription start site

Transcription start site (contains a 23 contains a GC rich spacer immediately after the TATA box for optimal spacing and a transcription start motif immediately after the spacer)

ABPshort
GTTAATTTTTGTGGCCCTTGCGATGTTTGCTCTGGTTAATAATCTCAGGACAAACA

GTTA HNF-1-1 transcription factor binding site
HNF-4 transcription factor binding site
HNF-3a transcription factor binding site
HNF1-2 transcription factor binding site
HNF-3-2 transcription factor binding site

Complete Promoter Sequences

HNF1-shortABP-SynO-TSS (designated the Hepatic Combinitoral Bundle, or HCB)
5’GTTAATCATTAAGTCGTTAATTTTTGTGGCCCTTGCGATGTTTGCTCTGGTTAATAATCTCAGGACAAACAGAGGT
TAATAATTTTCCAGATCTCTCTGAGCAATAGTATAAAAGGCCAGCAGCAGCCTGACCACATCTCATCCTC3’

HNF1a
shortABP
SynO
CCTC TSS

ABPshort-HP1-AFP-TSS
5’GTTAATTTTTGTGGCCCTTGCGATGTTTGCTCTGGTTAATAATCTCAGGACAAACATACATTTTCAGTCATATGTTT
GCTCACTGAAGGTTACTAGTTAACAGGCATCCCTTAAACAGGATATAAAAGGCCAGCAGCAGCCTGACCACATCTC
ATCCTC3’

shortABP
HP1

AFP
TSS

ABPnat-HP1-AFP-TSS

5'GTTAATTTTTAAAAAGCAGTCAAAAGTCCAAGTGGCCCTTGCGAGCATTTACTCTCTCTGTTTGCTCTGGTTAATA
ATCTCAGGAGCACAAACAGAGGTTAATAATTTTCAGTCATATGTTTGCTCACTGAAGGTTACTAGTTAACAGGCAT
CCCTTAAACAGGATATAAAAGGCCAGCAGCAGCCTGACCACATCTCATCCTC3'

ABP enhancer
HP1
AFP
Transcription start site

HNF1-ABP-SynO

5'GTTAATCATTAAGTCGTTAATTTTTAAAAAGCAGTCAAAAGTCCAAGTGGCCCTTGCGAGCATTTACTCTCTCTGT
TTGCTCTGGTTAATAATCTCAGGAGCACAAACAGAGGTTAATAATTTTCCAGATCTCTCTGAGCAATAGTATAAAA3

HNF1 transcription factor binding site
ABP enhancer
SynO

SP1-ABP-SynO

5'TGGGCGGAGTGTCGTTAATTTTTAAAAAGCAGTCAAAAGTCCAAGTGGCCCTTGCGAGCATTTACTCTCTCTGTT
TGCTCTGGTTAATAATCTCAGGAGCACAAACAGAGGTTAATAATTTTCCAGATCTCTCTGAGCAATAGTATAAAA3'

HNF1 transcription factor binding site
ABP enhancer
SynO

ABP-SynO

5'GTTAATTTTTAAAAAGCAGTCAAAAGTCCAAGTGGCCCTTGCGAGCATTTACTCTCTCTGTTTGCTCTGGTTAATA
ATCTCAGGAGCACAAACAGAGGTTAATAATTTTCCAGATCTCTCTGAGCAATAGTATAAAA3'

ABP element
SynO element

ABP-HP1-AFP

5'GTTAATTTTTAAAAAGCAGTCAAAAGTCCAAGTGGCCCTTGCGAGCATTTACTCTCTCTGTTTGCTCTGGTTAATA
ATCTCAGGAGCACAAACAGAGGTTAATAATTTTCAGTCATATGTTTGCTCACTGAAGGTTACTAGTTAACAGGCAT
CCCTTAAACAGGATATAAAA3'

ABP element
HP1 element
AFP element

FVIII sequences

An53-HCO

ATGCAGATTGAGCTGTCCACTTGCTTTTTCCTGTGCCTGCTGCAGTTTTCATTTTCCGCCACTAGAAGATACTACCTG
GGGGCTGTCGAACTGTCCTGGGATTACATGCAGTCCGACCTGCTGTCTGAGCTGCATGTGGACACCCGATTTCCAC
CTCGCGTCCCACGAAGCTTCCCCTTTAATACATCCGTGATGTACAAGAAAACTGTGTTCGTCGAGTTCACCGATCAC
CTGTTCAACATCGCAAAGCCCCGGCCACCCTGGATGGGACTGCTGGGCCCTACCATCAGAGCCGAGGTGTACGAC
ACCGTGGTCATTACACTGAAAAACATGGCAAGTCACCCCGTGTCACTGCATGCCGTGGGAGTCTCCTACTGGAAG
GCATCTGAAGGCGCCGAGTATGACGATCAGACTAGTCAGAGAGAAAAAGAGGACGATAAGGTGTTTCCCGGAGA
ATCTCATACCTATGTGTGGCAGGTCCTGAAGGAGAATGGCCCTATGGCCAGCGACCCTCCATGCCTGACCTACTCC
TATCTGTCTCACGTGGACCTGGTCAAAGATCTGAACTCCGGGCTGATCGGAGCCCTGCTGGTGTGTCGGGAAGGA
TCTCTGGCTAAGGAGAGAACCCAGACACTGCATCAGTTCGTGCTGCTGTTCGCTGTCTTTGACGAAGGCAAAAGTT
GGCACTCAGAGACAAAGGATTCCCTGACTCAGGCAATGGACTCTGCCAGTGCTAGGGCATGGCCAAAAATGCACA
CCGTGAACGGCTACGTCAATAGAAGCCTGCCAGGACTGATCGGATGCCACAGGAAGTCCGTGTATTGGCATGTCA
TCGGCATGGGGACCACACCAGAAGTCCACTCTATTTTCCTGGAGGGACATACATTTCTGGTGCGGAATCACAGACA
GGCTAGCCTGGAGATCTCCCCCATTACCTTCCTGACAGCACAGACTCTGCTGATGGATCTGGGCCAGTTCCTGCTG
TTTTGCCACATCAGCTCCCACCAGCATGATGGGATGGAGGCCTACGTGAAAGTCGACAGCTGTCCAGAGGAACCC
CAGCTGAGGATGAAGAACAATGAGGAAGAGGAAGACTACGACGATGACCTGTATGACAGCGAGATGGATGTGG
TCCGATTCGATGACGATAACTCACCCCCTTTTATCCAGATTAGAAGCGTCGCCAAGAAACACCCTAAGACTTGGGT
GCATTACATCGCCGCTGAGGAAGAGGACTGGGATTATGCTCCTTCCGTGCTGACCCCAGACGATCGCAGCTACAA
ATCCCAGTATCTGAACAATGGCCCTCAGAGGATTGGGCGCAAGTACAAGAAAGTGAGGTTCATGGCTTATACCGA
TGAAACCTTCAAGACTCGCGAAGCAATCCAGTACGAGTCCGGAATTCTGGGCCCACTGCTGTATGGGGAAGTGGG
AGACACCCTGCTGATCATTTTCAAGAACCAGGCCTCTAGGCCCTACAATATCTATCCTCATGGCATTACAGATGTGT
CTCCCCTGCACAGTGGACGCCTGCCTAAGGGCGTGAAACACCTGAAGGACCTGCCTATCCTGCCAGGGGAAATTT
TTAAGTACAAATGGACTGTGACCGTCGAGGATGGACCAACTAAGAGCGACCCCAGGTGCCTGACCCGCTACTATT
CTAGTTTCATCAATCTGGAAAGAGATCTGGCAAGCGGACTGATCGGACCACTGCTGATTTGTTACAAAGAGTCCGT
GGATCAGCGAGGCAACCAGATGATGTCTGACAAGCGGAATGTGATCCTGTTCTCAGTCTTTGACGAAAACCGCAG
CTGGTATCTGACCGAGAACATGCAGCGATTCCTGCCAAATGCAGCAGGAGTGCAGCCACAGGATCCTGAGTTTCA
GGCTAGTAACATCATGCATTCAATTAATGGCTACGTGTTCGACTCACTGCAGCTGAGCGTGTGTCTGCACGAGGTC
GCTTACTGGTATATCCTGAGCGTCGGAGCACAGACAGATTTCCTGTCCGTGTTCTTTTCTGGCTACACTTTTAAGCA
TAAAATGGTGTATGAGGACACACTGACTCTGTTCCCTTTTTCCGGCGAAACCGTCTTTATGTCTATGGAGAATCCAG
GGCTGTGGGTGCTGGGATGCCACAACTCCGATTTCCGGAATAGAGGAATGACTGCCCTGCTGAAAGTGTCAAGCT
GTGACCGGAACACCGGCGACTACTATGAAGATACATACGAGGACATCCCAACTTATCTGCTGTCTGAAAACAATGT
GATTGAGCCCAGAAGCTTCAGCCAGAATCCACCCGTGCTGAAGCGACACCAGCGGGAAATCACCCTGACTACCCT
GCAGTCAGAGCAGGAAGAGATTGATTACGACGATACCATCAGCATTGAAACAAAAAGGGAGGACTTCGATATCTA
TGGGGAAGACGAGAACCAGGGACCTCGCTCCTTCCAGAAGAGGACACGCCATTACTTTATTGCTGCAGTGGAGAG
GCTGTGGGATTATGGGATGTCCCGCTCTCCCCACGTCCTGCGAAATCGGGCCCAGAGTGGATCAGTGCCTCAGTTC
AAGAAAGTGGTCTTCCAGGAGTTTACTGACGGGAGCTTTACCCAGCCTCTGTACCGGGGAGAACTGAACGAGCAC
CTGGGACTGCTGGGCCCATATATCAGAGCAGAAGTGGAGGATAACATTATGGTCACCTTCAAGAATCAGGCCAGT
CGGCCCTACTCATTTTATTCCTCTCTGATCAGCTACGAAGAGGACCAGCGCCAGGGCGCAGAACCACGAAAAAACT
TCGTGAAGCCCAATGAGACCAAAACATACTTTTGGAAGGTGCAGCACCATATGGCTCCTACAAAAGACGAATTCG
ATTGCAAGGCCTGGGCTTATTTTAGTGACGTGGATCTGGAGAAGGACATGCACTCAGGGCTGATCGGACCTCTGC
TGATTTGTCATACTAACACCCTGAATCCAGCACACGGACGACAGGTGACAGTCCAGGAATTCGCTCTGTTCTTTAC
AATCTTCGATGAGACTAAGAGCTGGTACTTCACTGAAAACATGGAGAGAAATTGCAGGGCCCCTTGTAATATCCA
GATGGAAGACCCAACATTCAAGGAGAACTACAGATTTCATGCTATTAATGGCTATGTGATGGATACTCTGCCAGG

GCTGGTCATGGCACAGGACCAGAGAATCAGGTGGTACCTGCTGTCTATGGGGAGTAACGAGAATATCCACAGCAT
TCATTTCTCCGGACACGTGTTTACTGTCAGGAAGAAAGAAGAGTATAAAATGGCCGTGTACAACCTGTATCCAGGC
GTGTTCGAAACCGTCGAGATGCTGCCAAGCAAGGCAGGAATCTGGCGAGTGGAATGCCTGATTGGCGAGCACCT
GCATGCTGGGATGAGTACCCTGTTTCTGGTGTACTCAAAACAGTGTCAGACACCTCTGGGAATGGCATCTGGCCAT
ATCCGGGATTTCCAGATTACCGCAAGTGGACAGTACGGACAGTGGGCTCCAAAGCTGGCAAGACTGCACTATAGC
GGCTCCATCAACGCCTGGTCTACAAAAGAGCCCTTTAGTTGGATTAAGGTGGACCTGCTGGCCCCTATGATCATTC
ATGGCATCAAAACTCAGGGGGCTAGGCAGAAGTTCAGTTCACTGTACATCAGCCAGTTTATCATCATGTACTCCCT
GGATGGGAAGAAATGGCAGACCTACCGCGGGAATAGCACAGGAACTCTGATGGTGTTCTTTGGAAACGTCGACA
GCTCCGGCATCAAGCACAACATTTTCAATCCTCCAATCATTGCCCGCTACATCCGACTGCACCCCACCCATTATTCAA
TTCGAAGCACACTGCGGATGGAACTGATGGGCTGCGATCTGAACTCTTGTAGTATGCCTCTGGGGATGGAGTCTA
AGGCCATCAGTGACGCTCAGATTACCGCATCTAGTTACTTCACCAATATGTTTGCCACATGGTCACCAAGCCAGGC
TAGGCTGCACCTGCAGGGAAGAACAAACGCCTGGAGGCCTCAGGTGAACAATCCAAAGGAGTGGCTGCAGGTGG
ATTTCCAGAAAACTATGAAGGTCACCGGAATCACAACTCAGGGCGTGAAATCACTGCTGACCAGCATGTATGTGA
AGGAGTTTCTGATTTCAAGCTCCCAGGACGGCCACCATTGGACACTGTTCCTGCAGAACGGGAAGGTGAAAGTCT
TCCAGGGAAATCAGGATTCTTTTACACCAGTGGTCAACAGTCTGGACCCCCCTCTGCTGACTCGGTACCTGAGAAT
CCACCCCCAGAGCTGGGTCCATCAGATTGCACTGCGACTGGAAGTGCTGGGATGCGAGGCACAGCAGCTGTATTG
A

An53-LCO
ATGCAGATTGAGCTGAGCACCTGCTTCTTCCTGTGCCTGCTGCAGTTCTCATTCTCTGCCACCAGGAGATACTACCT
GGGCGCCGTGGAGCTGAGCTGGGACTACATGCAGTCTGACCTGCTGTCTGAGCTGCATGTGGACACCAGGTTCCC
CCCCAGAGTGCCCCGAAGCTTCCCCTTCAACACCAGCGTGATGTACAAGAAGACCGTGTTCGTGGAGTTCACTGAC
CACCTGTTCAACATCGCCAAGCCCAGGCCCCCCTGGATGGGCCTGCTGGGCCCCACCATCAGAGCCGAGGTGTAC
GACACCGTGGTCATCACCCTGAAGAACATGGCCAGCCACCCCGTCTCCCTGCACGCCGTGGGGGTGAGCTACTGG
AAGGCCTCTGAGGGCGCCGAGTACGACGACCAGACCAGCCAGAGGGAGAAGGAGGACGACAAGGTGTTCCCTG
GGGAAAGCCACACCTACGTGTGGCAGGTCCTGAAGGAGAACGGCCCCATGGCCTCTGACCCCCCATGCCTGACCT
ACAGCTACCTGAGCCACGTGGACCTGGTGAAGGACCTGAACTCTGGCCTGATTGGGGCCCTGCTGGTGTGCAGGG
AGGGCAGCCTGGCCAAGGAGAGAACCCAGACCCTGCACCAGTTCGTGCTGCTGTTCGCCGTGTTCGACGAGGGC
AAGAGCTGGCACTCTGAAACCAAGGATAGCCTGACTCAGGCCATGGACTCTGCCTCTGCCAGGGCCTGGCCCAAG
ATGCACACCGTCAACGGCTACGTCAACAGGAGCCTGCCTGGCCTGATTGGCTGCCACAGGAAGAGCGTGTACTGG
CATGTGATCGGCATGGGCACCACCCCTGAGGTGCACAGCATCTTCCTGGAGGGCCACACCTTCCTGGTCAGGAAC
CACAGGCAGGCCAGCCTGGAGATCAGCCCCATCACCTTCCTGACCGCCCAGACCCTGCTGATGGACCTGGGCCAG
TTCCTGCTGTTCTGCCACATCTCCAGCCACCAGCACGACGGCATGGAGGCCTACGTGAAAGTGGACAGCTGCCCTG
AGGAGCCCCAGCTGAGGATGAAGAACAACGAGGAGGAGGAGGACTATGATGACGACCTGTATGACAGCGAGAT
GGACGTGGTCAGGTTCGACGACGACAACAGCCCCCCTTTCATCCAGATCAGGAGCGTGGCCAAGAAGCACCCCAA
GACCTGGGTGCACTACATCGCTGCTGAGGAGGAGGACTGGGACTATGCCCCCTCCGTGCTGACCCCTGATGACAG
GAGCTACAAGAGCCAGTACCTGAACAATGGCCCCCAGAGGATTGGCAGGAAGTACAAGAAAGTCAGGTTCATGG
CCTACACTGATGAAACCTTCAAGACCAGGGAGGCCATCCAGTACGAGTCTGGCATCCTGGGCCCCCTGCTGTACG
GGGAGGTGGGGGACACCCTGCTGATCATCTTCAAGAACCAGGCCAGCAGGCCCTACAACATCTACCCCCATGGCA
TCACCGACGTGAGCCCCCTGCACAGCGGAAGGCTGCCTAAGGGGGTGAAGCACCTGAAAGACCTGCCCATCCTGC
CTGGGGAGATCTTCAAGTACAAGTGGACTGTGACTGTGGAGGACGGCCCCACCAAGAGCGACCCCAGGTGCCTG
ACCAGATACTACAGCAGCTTCATCAACCTGGAGAGGGACCTGGCCTCTGGCCTGATTGGCCCCCTGCTGATCTGCT
ACAAGGAGTCTGTGGACCAGAGGGGCAACCAGATGATGAGCGACAAGAGGAACGTGATCCTGTTCTCTGTCTTC
GACGAGAACAGGAGCTGGTACCTGACCGAGAACATGCAGAGGTTCCTGCCCAACGCAGCTGGGGTGCAGCCACA
GGACCCCGAGTTCCAGGCCAGCAACATCATGCACAGCATCAATGGCTACGTGTTCGACAGCCTGCAGCTGAGCGT
GTGCCTGCACGAGGTGGCCTACTGGTACATCCTGAGCGTCGGCGCCCAGACCGACTTCCTGAGCGTGTTCTTCTCT
GGCTACACCTTCAAGCACAAGATGGTGTATGAGGACACCCTGACCCTGTTCCCCTTCAGCGGGGAGACTGTCTTCA
TGAGCATGGAGAACCCTGGCCTGTGGGTGCTGGGCTGCCACAACAGCGACTTCAGGAACAGGGGCATGACTGCC

CTGCTGAAAGTCTCCAGCTGTGACCGGAACACCGGGGACTACTACGAGGACACATACGAGGACATCCCAACTTAC
CTGCTGAGCGAAAACAATGTGATCGAGCCCAGGAGCTTCTCTCAGAACCCCCCAGTGCTGAAGAGGCACCAGAGG
GAGATCACCCTGACCACCCTGCAGTCTGAGCAGGAGGAGATCGACTATGATGACACCATCAGCATTGAGACAAAG
AGGGAGGACTTCGACATCTACGGGGAGGACGAGAACCAGGGACCCAGGAGCTTCCAGAAGAGGACCAGGCACT
ACTTCATTGCTGCTGTGGAGAGGCTGTGGGACTATGGCATGTCCCGCAGCCCCCATGTGCTGAGGAACAGGGCCC
AGTCTGGCAGCGTGCCCCAGTTCAAGAAAGTCGTGTTCCAGGAGTTCACCGACGGCAGCTTCACCCAGCCCCTGTA
CAGAGGGGAGCTGAACGAGCACCTGGGCCTGCTGGGCCCCTACATCAGGGCCGAGGTGGAGGACAACATCATG
GTGACCTTCAAGAACCAGGCCAGCAGGCCCTACAGCTTCTACAGCAGCCTGATCAGCTACGAGGAGGACCAGAGG
CAGGGGGCTGAGCCCAGGAAGAACTTTGTGAAGCCCAATGAAACCAAGACCTACTTCTGGAAGGTGCAGCACCA
CATGGCCCCCACCAAGGACGAGTTCGACTGCAAGGCCTGGGCCTACTTCTCTGACGTGGACCTGGAGAAGGACAT
GCACTCTGGCCTGATTGGCCCCCTGCTGATTTGCCACACCAACACCCTGAACCCTGCCCATGGCAGGCAGGTGACT
GTGCAGGAGTTCGCCCTGTTCTTCACCATCTTCGATGAAACCAAGAGCTGGTACTTCACTGAGAACATGGAGAGG
AACTGCAGGGCCCCCTGCAACATCCAGATGGAGGACCCCACCTTCAAGGAGAACTACAGGTTCCATGCCATCAAT
GGCTACGTGATGGACACCCTGCCTGGCCTGGTCATGGCCCAGGACCAGAGGATCAGGTGGTATCTGCTGAGCATG
GGCAGCAACGAGAACATCCACAGCATCCACTTCTCTGGCCACGTGTTCACTGTGAGGAAGAAGGAGGAGTACAAG
ATGGCCGTGTACAACCTGTACCCTGGGGTGTTCGAAACCGTGGAGATGCTGCCCAGCAAGGCCGGCATCTGGAG
GGTGGAGTGCCTGATTGGGGAGCACCTGCACGCCGGCATGAGCACCCTGTTCCTGGTGTACAGCAAACAGTGCCA
GACCCCCCTGGGCATGGCCTCTGGCCACATCAGGGACTTCCAGATCACTGCCTCTGGCCAGTACGGCCAGTGGGC
CCCCAAGCTGGCCAGGCTGCACTACTCCGGAAGCATCAATGCCTGGAGCACCAAGGAGCCCTTCAGCTGGATCAA
AGTGGACCTGCTGGCCCCCATGATCATCCACGGCATCAAGACCCAGGGGGCCAGGCAGAAGTTCTCCAGCCTGTA
CATCAGCCAGTTCATCATCATGTACAGCCTGGACGGCAAGAAGTGGCAGACCTACAGGGGCAACAGCACCGGCAC
CCTGATGGTGTTCTTCGGCAACGTGGACAGCAGCGGCATCAAGCACAACATCTTCAACCCCCCCATCATCGCCAGA
TACATCAGGCTGCACCCCACCCACTACAGCATCAGGAGCACCCTGAGGATGGAGCTGATGGGCTGTGACCTGAAC
AGCTGCAGCATGCCCCTGGGCATGGAGAGCAAGGCCATCTCTGACGCCCAGATCACTGCCTCCAGCTACTTCACCA
ACATGTTTGCCACCTGGAGCCCCAGCCAGGCCAGGCTGCACCTGCAGGGCAGGACAAATGCCTGGAGGCCCCAG
GTCAACAACCCCAAGGAGTGGCTGCAGGTGGACTTCCAGAAGACCATGAAGGTGACTGGGATCACCACCCAGGG
GGTGAAGAGCCTGCTGACCAGCATGTACGTGAAGGAGTTCCTGATCTCCAGCAGCCAGGACGGCCACCATTGGAC
CCTGTTCCTGCAGAATGGCAAGGTGAAGGTGTTCCAGGGCAACCAGGACAGCTTCACCCCTGTGGTCAACAGCCT
GGACCCCCCCCTGCTGACCAGATACCTGAGGATCCACCCCCAGAGCTGGGTGCACCAGATCGCCCTGAGGCTGGA
GGTGCTGGGCTGTGAGGCCCAGCAGCTGTACTGA

ET3-LCO with CpGs

ATGCAGCTGGAACTGTCTACCTGTGTGTTTCTGTGTCTGCTGCCTCTGGGGTTTTCTGCT
ATCCGCCGCTACTATCTGGGAGCCGTGGAGCTGTCCTGGGACTACAGGCAGAGCGAGCTG
CTGAGAGAACTGCACGTGGATACCAGATTCCCAGCTACCGCTCCAGGAGCTCTGCCTCTG
GGCCCATCCGTGCTGTACAAGAAAACCGTCTTCGTGGAGTTTACCGACCAGCTGTTCAGC
GTGGCCAGGCCAAGACCACCTTGGATGGGACTGCTGGGACCAACCATCCAGGCTGAGGTG
TACGATACCGTGGTCGTGACCCTGAAAAACATGGCCTCCCATCCCGTGAGCCTGCACGCT
GTCGGGGTGTCCTTCTGGAAGTCCAGCGAGGGAGCCGAGTACGAAGACCATACCTCCCAG
CGCGAGAAAGAAGACGATAAGGTGCTGCCTGGCAAAAGCCAGACCTATGTCTGGCAGGTG
CTGAAGGAGAACGGACCAACCGCTAGCGACCCACCATGCCTGACCTACTCTTATCTGTCC
CACGTCGATCTGGTGAAGGACCTGAATTCCGGACTGATCGGAGCTCTGCTGGTGTGTAGA
GAGGGAAGCCTGACCAGAGAAAGAACCCAGAACCTGCATGAGTTCGTCCTGCTGTTCGCC
GTGTTTGACGAAGGGAAGAGCTGGCACTCTGCCCGCAATGACTCCTGGACCAGAGCTATG
GATCCAGCTCCTGCTAGAGCTCAGCCTGCTATGCACACCGTCAACGGCTACGTGAATCGG
TCTCTGCCAGGACTGATCGGCTGCCATAAGAAAAGCGTCTATTGGCACGTGATCGGAATG
GGCACCAGCCCCGAGGTGCATTCTATCTTCCTGGAAGGCCACACCTTTCTGGTCAGGCAC
CATAGACAGGCCTCTCTGGAGATCTCCCCTCTGACCTTCCTGACCGCTCAGACCTTTCTG

ATGGACCTGGGGCAGTTCCTGCTGTTTTGCCATATCTCTTCCCACCATCACGGAGGAATG
GAGGCTCACGTCAGGGTGGAATCCTGTGCTGAGGAACCACAGCTGAGAAGAAAGGCTGAT
GAGGAAGAGGACTACGACGATAACCTGTATGACAGCGATATGGACGTCGTGCGCCTGGAC
GGCGACGATGTCAGCCCTTTCATCCAGATCCGGTCTGTGGCCAAGAAACATCCAAAGACC
TGGGTCCACTACATCGCCGCTGAAGAGGAAGATTGGGACTATGCCCCCCTGGTGCTGGCT
CCTGACGATAGATCCTACAAAAGCCAGTATCTGAACAATGGGCCCCAGCGCATCGGACGG
AAGTACAAGAAAGTGAGGTTCATGGCCTATACCGACGAGACCTTTAAGACCAGAGAGGCT
ATCCAGCACGAATCCGGGATCCTGGGACCTCTGCTGTACGGCGAAGTGGGGGATACCCTG
CTGATCATCTTCAAGAACCAGGCCTCCAGGCCATACAATATCTATCCCCATGGCATCACC
GACGTGAGACCACTGTACAGCAGGAGACTGCCCAAGGGGGTCAAACACCTGAAGGATTTC
CCCATCCTGCCTGGAGAGATCTTTAAGTATAAATGGACCGTCACCGTGGAAGACGGGCCT
ACCAAGTCCGATCCACGCTGCCTGACCCGGTACTATAGCTCTTTCGTGAACATGGAGAGA
GACCTGGCTAGCGGACTGATCGGACCCCTGCTGATCTGTTACAAAGAGAGCGTGGACCAG
AGGGGCAACCAGATCATGTCTGATAAGAGAAATGTCATCCTGTTCTCCGTGTTTGACGAG
AACCGCAGCTGGTACCTGACCGAGAACATCCAGCGGTTCCTGCCAAATCCAGCTGGAGTG
CAGCTGGAGGACCCAGAATTTCAGGCTTCCAACATCATGCATAGCATCAATGGCTACGTG
TTCGATAGCCTGCAGCTGTCTGTCTGCCTGCACGAGGTGGCCTACTGGTATATCCTGTCC
ATCGGCGCTCAGACCGACTTCCTGTCCGTGTTCTTTAGCGGGTACACCTTTAAGCATAAA
ATGGTGTATGAGGATACCCTGACCCTGTTCCCCTTTTCTGGCGAGACCGTGTTCATGTCC
ATGGAAAACCCTGGCCTGTGGATCCTGGGGTGCCACAACAGCGACTTCAGGAATAGAGGA
ATGACCGCCCTGCTGAAAGTGTCCAGCTGTGATAAGAATACCGGCGATTACTATGAGGAC
TCTTACGAAGATATCTCCGCTTATCTGCTGAGCAAGAACAATGCCATCGAGCCCAGGTCT
TTCGCTCAGAACTCCAGACCTCCAAGCGCTTCTGCTCCTAAGCCACCTGTGCTGAGAAGA
CATCAGAGGGACATCTCCCTGCCTACCTTCCAGCCAGAGGAAGATAAAATGGACTACGAC
GATATCTTCAGCACCGAGACCAAGGGGGAAGATTTTGACATCTATGGAGAGGACGAAAAC
CAGGATCCAAGATCCTTCCAGAAGAGAACCAGACACTACTTTATCGCCGCTGTGGAGCAG
CTGTGGGACTATGGGATGTCCGAAAGCCCACGGGCCCTGAGGAACAGAGCTCAGAATGGA
GAGGTGCCCCGCTTCAAGAAAGTCGTGTTCCGGGAGTTTGCCGACGGCAGCTTTACCCAG
CCATCTTACAGGGGGGAGCTGAACAAGCATCTGGGGCTGCTGGGACCCTATATCAGAGCC
GAGGTCGAAGATAACATCATGGTGACCTTCAAGAATCAGGCTTCTCGCCCCTACTCCTTT
TATTCTTCCCTGATCTCCTACCCTGACGATCAGGAGCAGGGCGCCGAACCTAGGCACAAC
TTCGTGCAGCCAAATGAGACCAGAACCTACTTTTGGAAGGTGCAGCATCACATGGCTCCC
ACCGAGGATGAATTCGACTGCAAAGCTTGGGCCTATTTTTCCGATGTCGACCTGGAGAAG
GACGTGCATAGCGGCCTGATCGGGCCTCTGCTGATCTGTCGCGCCAACACCCTGAATGCT
GCTCACGGAAGACAGGTCACCGTGCAGGAGTTCGCTCTGTTCTTTACCATCTTTGACGAA
ACCAAGAGCTGGTACTTCACCGAGAACGTGGAAAGGAATTGCAGAGCCCCCTGTCATCTG
CAGATGGAGGACCCTACCCTGAAGGAAAACTACAGGTTCCACGCCATCAATGGATATGTC
ATGGATACCCTGCCCGGCCTGGTCATGGCTCAGAACCAGCGCATCCGGTGGTACCTGCTG
TCTATGGGATCCAACGAGAATATCCATAGCATCCACTTCTCTGGCCATGTCTTTTCCGTG
AGGAAGAAAGAGGAATACAAAATGGCCGTGTACAATCTGTATCCTGGGGTCTTCGAGACC
GTGGAAATGCTGCCAAGCAAAGTGGGAATCTGGAGAATCGAGTGCCTGATCGGCGAACAC
CTGCAGGCCGGGATGAGCACCACCTTCCTGGTGTACTCTAAGAAATGTCAGACCCCACTG
GGGATGGCCTCCGGACATATCCGCGACTTCCAGATCACCGCTAGCGGACAGTACGGACAG
TGGGCTCCAAAGCTGGCTAGACTGCACTATTCTGGCTCCATCAACGCCTGGTCTACCAAA
GAGCCATTCTCCTGGATCAAGGTGGACCTGCTGGCCCCCATGATCATCCACGGAATCAAA
ACCCAGGGCGCTAGGCAGAAGTTCAGCTCTCTGTACATCTCCCAGTTTATCATCATGTAT
AGCCTGGACGGGAAGAAATGGCAGACCTACAGAGGCAATTCCACCGGGACCCTGATGGTC
TTCTTTGGAAACGTGGATTCCAGCGGCATCAAGCACAACATCTTCAATCCACCCATCATC

GCCCGCTACATCCGGCTGCATCCTACCCACTATAGCATCAGGTCTACCCTGAGAATGGAG
CTGATGGGATGCGACCTGAACAGCTGTTCTATGCCACTGGGCATGGAGTCCAAGGCTATC
AGCGATGCCCAGATCACCGCTTCTTCCTACTTCACCAATATGTTTGCTACCTGGTCCCCA
AGCAAGGCTAGACTGCACCTGCAGGGAAGATCCAACGCTTGGAGACCCCAGGTGAACAAT
CCTAAGGAGTGGCTGCAGGTCGACTTCCAGAAAACCATGAAGGTCACCGGGGTGACCACC
CAGGGAGTGAAATCTCTGCTGACCTCCATGTACGTCAAGGAGTTCCTGATCAGCTCTTCC
CAGGACGGCCACCAGTGGACCCTGTTCTTTCAGAACGGCAAGGTCAAAGTGTTCCAGGGG
AATCAGGACTCTTTTACCCCCGTCGTGAACTCCCTGGATCCTCCACTGCTGACCAGGTAC
CTGAGAATCCATCCTCAGAGCTGGGTGCACCAGATCGCTCTGAGAATGGAGGTCCTGGGA
TGCGAAGCTCAGGACCTGTATTGA

<u>ET3-LCO CpGs removed</u>

ATGCAGCTGGAACTGTCTACCTGTGTGTTTCTGTGTCTGCTGCCTCTGGGGTTTTCTGCT
ATCAGGAGATACTATCTGGGAGCTGTGGAGCTGTCCTGGGACTACAGGCAGTCTGAGCTG
CTGAGAGAACTGCATGTGGATACCAGATTCCCAGCTACAGCTCCAGGAGCTCTGCCTCTG
GGCCCATCTGTGCTGTACAAGAAAACAGTCTTTGTGGAGTTTACAGACCAGCTGTTCTCT
GTGGCCAGGCCAAGACCACCTTGGATGGGACTGCTGGGACCAACCATCCAGGCTGAGGTG
TATGATACAGTGGTGGTGACCCTGAAAAACATGGCCTCCCATCCTGTGAGCCTGCATGCT
GTGGGGGTGTCCTTCTGGAAGTCCTCTGAGGGAGCTGAGTATGAAGACCATACCTCCCAG
AGGGAGAAAGAAGATGATAAGGTGCTGCCTGGCAAAAGCCAGACCTATGTCTGGCAGGTG
CTGAAGGAGAATGGACCAACTGCTTCTGACCCACCATGCCTGACCTACTCTTATCTGTCC
CATGTGGATCTGGTGAAGGACCTGAATTCTGGACTGATTGGAGCTCTGCTGGTGTGTAGA
GAGGGAAGCCTGACCAGAGAAAGAACCCAGAACCTGCATGAGTTTGTCCTGCTGTTTGCT
GTGTTTGATGAAGGGAAGAGCTGGCACTCTGCCAGGAATGACTCCTGGACCAGAGCTATG
GATCCAGCTCCTGCTAGAGCTCAGCCTGCTATGCACACAGTCAATGGCTATGTGAATAGG
TCTCTGCCAGGACTGATTGGCTGCCATAAGAAATCTGTCTATTGGCATGTGATTGGAATG
GGCACCAGCCCTGAGGTGCATTCTATCTTCCTGGAAGGCCACACCTTTCTGGTCAGGCAC
CATAGACAGGCCTCTCTGGAGATCTCCCCTCTGACCTTCCTGACAGCTCAGACCTTTCTG
ATGGACCTGGGGCAGTTCCTGCTGTTTTGCCATATCTCTTCCCACCATCATGGAGGAATG
GAGGCTCATGTCAGGGTGGAATCCTGTGCTGAGGAACCACAGCTGAGAAGAAAGGCTGAT
GAGGAAGAGGACTATGATGATAACCTGTATGACTCTGATATGGATGTGGTGAGGCTGGAT
GGGGATGATGTCAGCCCTTTCATCCAGATCAGGTCTGTGGCCAAGAAACATCCAAAGACC
TGGGTCCACTACATTGCTGCTGAAGAGGAAGATTGGGACTATGCCCCCCTGGTGCTGGCT
CCTGATGATAGATCCTACAAAAGCCAGTATCTGAACAATGGGCCCCAGAGGATTGGAAGG
AAGTACAAGAAAGTGAGGTTCATGGCCTATACAGATGAGACCTTTAAGACCAGAGAGGCT
ATCCAGCATGAATCTGGGATCCTGGGACCTCTGCTGTATGGAGAAGTGGGGGATACCCTG
CTGATCATCTTCAAGAACCAGGCCTCCAGGCCATACAATATCTATCCCCATGGCATCACA
GATGTGAGACCACTGTACAGCAGGAGACTGCCCAAGGGGGTCAAACACCTGAAGGATTTC
CCCATCCTGCCTGGAGAGATCTTTAAGTATAAATGGACAGTCACAGTGGAAGATGGGCCT
ACCAAGTCTGATCCAAGGTGCCTGACCAGATACTATAGCTCTTTTGTGAACATGGAGAGA
GACCTGGCTTCTGGACTGATTGGACCCCTGCTGATCTGTTACAAAGAGTCTGTGGACCAG
AGGGGCAACCAGATCATGTCTGATAAGAGAAATGTCATCCTGTTCTCTGTGTTTGATGAG
AACAGGAGCTGGTACCTGACAGAGAACATCCAGAGGTTCCTGCCAAATCCAGCTGGAGTG
CAGCTGGAGGACCCAGAATTTCAGGCTTCCAACATCATGCATAGCATCAATGGCTATGTG
TTTGATAGCCTGCAGCTGTCTGTCTGCCTGCATGAGGTGGCCTACTGGTATATCCTGTCC
ATTGGAGCTCAGACAGACTTCCTGTCTGTGTTCTTTAGTGGGTACACCTTTAAGCATAAA
ATGGTGTATGAGGATACCCTGACCCTGTTCCCCTTTTCTGGGGAGACAGTGTTCATGTCC

ATGGAAAACCCTGGCCTGTGGATCCTGGGGTGCCACAACTCTGACTTCAGGAATAGAGGA
ATGACAGCCCTGCTGAAAGTGTCCAGCTGTGATAAGAATACAGGGGATTACTATGAGGAC
TCTTATGAAGATATCTCTGCTTATCTGCTGAGCAAGAACAATGCCATTGAGCCCAGGTCT
TTTGCTCAGAACTCCAGACCTCCATCTGCTTCTGCTCCTAAGCCACCTGTGCTGAGAAGA
CATCAGAGGGACATCTCCCTGCCTACCTTCCAGCCAGAGGAAGATAAAATGGACTATGAT
GATATCTTCAGCACAGAGACCAAGGGGGAAGATTTTGACATCTATGGAGAGGATGAAAAC
CAGGATCCAAGATCCTTCCAGAAGAGAACCAGACACTACTTTATTGCTGCTGTGGAGCAG
CTGTGGGACTATGGGATGTCTGAAAGCCCAAGGGCCCTGAGGAACAGAGCTCAGAATGGA
GAGGTGCCCAGATTCAAGAAAGTGGTGTTCAGAGAGTTTGCTGATGGCAGCTTTACCCAG
CCATCTTACAGGGGGGAGCTGAACAAGCATCTGGGGCTGCTGGGACCCTATATCAGAGCT
GAGGTGGAAGATAACATCATGGTGACCTTCAAGAATCAGGCTTCTAGGCCCTACTCCTTT
TATTCTTCCCTGATCTCCTACCCTGATGATCAGGAGCAGGGAGCTGAACCTAGGCACAAC
TTTGTGCAGCCAAATGAGACCAGAACCTACTTTTGGAAGGTGCAGCATCACATGGCTCCC
ACAGAGGATGAATTTGACTGCAAAGCTTGGGCCTATTTTTCTGATGTGGACCTGGAGAAG
GATGTGCATTCTGGCCTGATTGGGCCTCTGCTGATCTGTAGGGCCAACACCCTGAATGCT
GCTCATGGAAGACAGGTCACAGTGCAGGAGTTTGCTCTGTTCTTTACCATCTTTGATGAA
ACCAAGAGCTGGTACTTCACAGAGAATGTGGAAAGGAATTGCAGAGCCCCCTGTCATCTG
CAGATGGAGGACCCTACCCTGAAGGAAAACTACAGGTTCCATGCCATCAATGGATATGTC
ATGGATACCCTGCCTGGCCTGGTCATGGCTCAGAACCAGAGGATCAGATGGTACCTGCTG
TCTATGGGATCCAATGAGAATATCCATAGCATCCACTTCTCTGGCCATGTCTTTTCTGTG
AGGAAGAAAGAGGAATACAAAATGGCTGTGTACAATCTGTATCCTGGGGTCTTTGAGACA
GTGGAAATGCTGCCAAGCAAAGTGGGAATCTGGAGAATTGAGTGCCTGATTGGGGAACAC
CTGCAGGCTGGGATGAGCACCACCTTCCTGGTGTACTCTAAGAAATGTCAGACCCCACTG
GGGATGGCCTCTGGACATATCAGGGACTTCCAGATCACAGCTTCTGGACAGTATGGACAG
TGGGCTCCAAAGCTGGCTAGACTGCACTATTCTGGCTCCATCAATGCCTGGTCTACCAAA
GAGCCATTCTCCTGGATCAAGGTGGACCTGCTGGCCCCCATGATCATCCATGGAATCAAA
ACCCAGGGAGCTAGGCAGAAGTTCAGCTCTCTGTACATCTCCCAGTTTATCATCATGTAT
AGCCTGGATGGGAAGAAATGGCAGACCTACAGAGGCAATTCCACTGGGACCCTGATGGTC
TTCTTTGGAAATGTGGATTCCTCTGGCATCAAGCACAACATCTTCAATCCACCCATCATT
GCCAGGTACATCAGGCTGCATCCTACCCACTATAGCATCAGGTCTACCCTGAGAATGGAG
CTGATGGGATGTGACCTGAACAGCTGTTCTATGCCACTGGGCATGGAGTCCAAGGCTATC
TCTGATGCCCAGATCACAGCTTCTTCCTACTTCACCAATATGTTTGCTACCTGGTCCCCA
AGCAAGGCTAGACTGCACCTGCAGGGAAGATCCAATGCTTGGAGACCCCAGGTGAACAAT
CCTAAGGAGTGGCTGCAGGTGGACTTCCAGAAAACCATGAAGGTCACAGGGGTGACCACC
CAGGGAGTGAAATCTCTGCTGACCTCCATGTATGTCAAGGAGTTCCTGATCAGCTCTTCC
CAGGATGGCCACCAGTGGACCCTGTTCTTTCAGAATGGCAAGGTCAAAGTGTTCCAGGGG
AATCAGGACTCTTTTACCCCAGTGGTGAACTCCCTGGATCCTCCACTGCTGACCAGGTAC
CTGAGAATCCATCCTCAGAGCTGGGTGCACCAGATTGCTCTGAGAATGGAGGTCCTGGGA
TGTGAAGCTCAGGACCTGTATTGA

ET3-NoCo
ATGCAGCTAGAGCTCTCCACCTGTGTCTTTCTGTGTCTCTTGCCACTCGGCTTTAGTGCCATCAGGAGATACTACCT
GGGCGCAGTGGAACTGTCCTGGGACTACCGGCAAAGTGAACTCCTCCGTGAGCTGCACGTGGACACCAGATTTCC
TGCTACAGCGCCAGGAGCTCTTCCGTTGGGCCCGTCAGTCCTGTACAAAAAGACTGTGTTCGTAGAGTTCACGGAT
CAACTTTTCAGCGTTGCCAGGCCCAGGCCACCATGGATGGGTCTGCTGGGTCCTACCATCCAGGCTGAGGTTTACG
ACACGGTGGTCGTTACCCTGAAGAACATGGCTTCTCATCCCGTTAGTCTTCACGCTGTCGGCGTCTCCTTCTGGAAA
TCTTCCGAAGGCGCTGAATATGAGGATCACACCAGCCAAAGGGAGAAGGAAGACGATAAAGTCCTTCCCGGTAA
AAGCCAAACCTACGTCTGGCAGGTCCTGAAAGAAAATGGTCCAACAGCCTCTGACCCACCATGTCTTACCTACTCA

TACCTGTCTCACGTGGACCTGGTGAAAGACCTGAATTCGGGCCTCATTGGAGCCCTGCTGGTTTGTAGAGAAGGG
AGTCTGACCAGAGAAAGGACCCAGAACCTGCACGAATTTGTACTACTTTTTGCTGTCTTTGATGAAGGGAAAAGTT
GGCACTCAGCAAGAAATGACTCCTGGACACGGGCCATGGATCCCGCACCTGCCAGGGCCCAGCCTGCAATGCACA
CAGTCAATGGCTATGTCAACAGGTCTCTGCCAGGTCTGATCGGATGTCATAAGAAATCAGTCTACTGGCACGTGAT
TGGAATGGGCACCAGCCCGGAAGTGCACTCCATTTTTCTTGAAGGCCACACGTTTCTCGTGAGGCACCATCGCCAG
GCTTCCTTGGAGATCTCGCCACTAACTTTCCTCACTGCTCAGACATTCCTGATGGACCTTGGCCAGTTCCTACTGTTT
TGTCATATCTCTTCCCACCACCATGGTGGCATGGAGGCTCACGTCAGAGTAGAAAGCTGCGCCGAGGAGCCCCAG
CTGCGGAGGAAAGCTGATGAAGAGGAAGATTATGATGACAATTTGTACGACTCGGACATGGACGTGGTCCGGCT
CGATGGTGACGACGTGTCTCCCTTTATCCAAATCCGCTCAGTTGCCAAGAAGCATCCTAAAACTTGGGTACATTAC
ATTGCTGCTGAAGAGGAGGACTGGGACTATGCTCCCTTAGTCCTCGCCCCCGATGACAGAAGTTATAAAAGTCAAT
ATTTGAACAATGGCCCTCAGCGGATTGGTAGGAAGTACAAAAAAGTCCGATTTATGGCATACACAGATGAAACCT
TTAAGACGCGTGAAGCTATTCAGCATGAATCAGGAATCTTGGGACCTTTACTTTATGGGGAAGTTGGAGACACACT
GTTGATTATATTTAAGAATCAAGCAAGCAGACCATATAACATCTACCCTCACGGAATCACTGATGTCCGTCCTTTGT
ATTCAAGGAGATTACCAAAAGGTGTAAAACATTTGAAGGATTTTCCAATTCTGCCAGGAGAAATATTCAAATATAA
ATGGACAGTGACTGTAGAAGATGGGCCAACTAAATCAGATCCGCGGTGCCTGACCCGCTATTACTCTAGTTTCGTT
AATATGGAGAGAGATCTAGCTTCAGGACTCATTGGCCCTCTCCTCATCTGCTACAAAGAATCTGTAGATCAAAGAG
GAAACCAGATAATGTCAGACAAGAGGAATGTCATCCTGTTTTCTGTATTTGATGAGAACCGAAGCTGGTACCTCAC
AGAGAATATACAACGCTTTCTCCCCAATCCAGCTGGAGTGCAGCTTGAGGATCCAGAGTTCCAAGCCTCCAACATC
ATGCACAGCATCAATGGCTATGTTTTTGATAGTTTGCAGTTGTCAGTTTGTTTGCATGAGGTGGCATACTGGTACAT
TCTAAGCATTGGAGCACAGACTGACTTCCTTTCTGTCTTCTTCTCTGGATATACCTTCAAACACAAAATGGTCTATGA
AGACACACTCACCCTATTCCCATTCTCAGGAGAAACTGTCTTCATGTCGATGGAAAACCCAGGTCTATGGATTCTG
GGGTGCCACAACTCAGACTTTCGGAACAGAGGCATGACCGCCTTACTGAAGGTTTCTAGTTGTGACAAGAACACT
GGTGATTATTACGAGGACAGTTATGAAGATATTTCAGCATACTTGCTGAGTAAAAACAATGCCATTGAACCTAGGA
GCTTTGCCCAGAATTCAAGACCCCCTAGTGCGAGCGCTCCAAAGCCTCCGGTCCTGCGACGGCATCAGAGGGACA
TAAGCCTTCCTACTTTTCAGCCGGAGGAAGACAAAATGGACTATGATGATATCTTCTCAACTGAAACGAAGGGAGA
AGATTTTGACATTTACGGTGAGGATGAAAATCAGGACCCTCGCAGCTTTCAGAAGAGAACCCGACACTATTTCATT
GCTGCGGTGGAGCAGCTCTGGGATTACGGGATGAGCGAATCCCCCCGGGCGCTAAGAAACAGGGCTCAGAACGG
AGAGGTGCCTCGGTTCAAGAAGGTGGTCTTCCGGGAATTTGCTGACGGCTCCTTCACGCAGCCGTCGTACCGCGG
GGAACTCAACAAACACTTGGGGCTCTTGGGACCCTACATCAGAGCGGAAGTTGAAGACAACATCATGGTAACTTT
CAAAAACCAGGCGTCTCGTCCCTATTCCTTCTACTCGAGCCTTATTTCTTATCCGGATGATCAGGAGCAAGGGGCA
GAACCTCGACACAACTTCGTCCAGCCAAATGAAACCAGAACTTACTTTTGGAAAGTGCAGCATCACATGGCACCCA
CAGAAGACGAGTTTGACTGCAAAGCCTGGGCCTACTTTTCTGATGTTGACCTGGAAAAAGATGTGCACTCAGGCTT
GATCGGCCCCCTTCTGATCTGCCGCGCCAACACCCTGAACGCTGCTCACGGTAGACAAGTGACCGTGCAAGAATTT
GCTCTGTTTTTCACTATTTTTGATGAGACAAAGAGCTGGTACTTCACTGAAAATGTGGAAAGGAACTGCCGGGCCC
CCTGCCATCTGCAGATGGAGGACCCCACTCTGAAAGAAAACTATCGCTTCCATGCAATCAATGGCTATGTGATGGA
TACACTCCCTGGCTTAGTAATGGCTCAGAATCAAAGGATCCGATGGTATCTGCTCAGCATGGGCAGCAATGAAAAT
ATCCATTCGATTCATTTTAGCGGACACGTGTTCAGTGTACGGAAAAAGGAGGAGTATAAAATGGCCGTGTACAAT
CTCTATCCGGGTGTCTTTGAGACAGTGGAAATGCTACCGTCCAAAGTTGGAATTTGGCGAATAGAATGCCTGATTG
GCGAGCACCTGCAAGCTGGGATGAGCACGACTTTCCTGGTGTACAGCAAGAAGTGTCAGACTCCCCTGGGAATGG
CTTCTGGACACATTAGAGATTTTCAGATTACAGCTTCAGGACAATATGGACAGTGGGCCCCAAAGCTGGCCAGACT
TCATTATTCCGGATCAATCAATGCCTGGAGCACCAAGGAGCCCTTTTCTTGGATCAAGGTGGATCTGTTGGCACCA
ATGATTATTCACGGCATCAAGACCCAGGGTGCCCGTCAGAAGTTCTCCAGCCTCTACATCTCTCAGTTTATCATCAT
GTATAGTCTTGATGGGAAGAAGTGGCAGACTTATCGAGGAAATTCCACTGGAACCTTAATGGTCTTCTTTGGCAAT
GTGGATTCATCTGGGATAAAACACAATATTTTTAACCCTCCAATTATTGCTCGATACATCCGTTTGCACCCAACTCAT
TATAGCATTCGCAGCACTCTTCGCATGGAGTTGATGGGCTGTGATTTAAATAGTTGCAGCATGCCATTGGGAATGG
AGAGTAAAGCAATATCAGATGCACAGATTACTGCTTCATCCTACTTTACCAATATGTTTGCCACCTGGTCTCCTTCA
AAAGCTCGACTTCACCTCCAAGGGAGGAGTAATGCCTGGAGACCTCAGGTGAATAATCCAAAAGAGTGGCTGCAA
GTGGACTTCCAGAAGACAATGAAAGTCACAGGAGTAACTACTCAGGGAGTAAAATCTCTGCTTACCAGCATGTAT

GTGAAGGAGTTCCTCATCTCCAGCAGTCAAGATGGCCATCAGTGGACTCTCTTTTTTCAGAATGGCAAAGTAAAGG
TTTTTCAGGGAAATCAAGACTCCTTCACACCTGTGGTGAACTCTCTAGACCCACCGTTACTGACTCGCTACCTTCGA
ATTCACCCCCAGAGTTGGGTGCACCAGATTGCCCTGAGGATGGAGGTTCTGGGCTGCGAGGCACAGGACCTCTAC
TGA

ET3-MCO
ATGCAGCTGGAGCTCTCAACCTGTGTGTTCCTCTGCCTGCTCCCCCTGGGATTTTCAGCTATCAGGAGATACTATCT
GGGAGCAGTGGAACTGTCCTGGGACTACAGGCAGTCAGAGCTGCTCAGAGAACTGCATGTGGATACTAGGTTCC
CTGCAACAGCTCCTGGAGCACTGCCACTGGGACCTTCAGTGCTGTACAAGAAAACTGTCTTTGTGGAGTTTACAGA
CCAGCTGTTCAGTGTGGCCAGGCCCAGGCCCCCCTGGATGGGGCTGCTGGGACCCACCATCCAGGCTGAAGTGTA
TGATACTGTGGTGGTGACCCTGAAAAACATGGCCTCTCATCCAGTCAGCCTGCATGCTGTGGGAGTGAGCTTCTGG
AAGAGCAGTGAGGGAGCTGAGTATGAAGACCATACCTCACAGAGGGAGAAAGAAGATGATAAGGTGCTGCCAG
GAAAAAGCCAGACCTATGTGTGGCAGGTGCTGAAGGAGAATGGCCCTACAGCTTCAGATCCTCCCTGCCTCACAT
ACTCTTATCTGAGCCATGTGGATCTGGTGAAGGACCTCAATAGTGGCCTGATTGGGGCACTGCTGGTGTGCAGAG
AGGGGTCCCTCACAAGGGAAAGAACTCAGAACCTGCATGAGTTTGTCCTGCTCTTTGCTGTGTTTGATGAGGGAA
AGTCCTGGCACTCAGCAAGGAATGACAGCTGGACCAGGGCTATGGACCCAGCACCAGCCAGAGCTCAGCCAGCT
ATGCACACTGTCAATGGCTATGTGAATAGGTCCCTGCCTGGACTCATTGGCTGCCATAAGAAATCAGTCTATTGGC
ATGTGATTGGAATGGGCACCAGCCCAGAGGTGCATTCCATCTTCCTGGAAGGCCACACATTTCTGGTCAGGCACCA
TAGACAGGCCAGCCTGGAGATCAGCCCACTGACTTTCCTCACAGCACAGACATTTCTGATGGACCTGGGGCAGTTC
CTGCTCTTTTGCCATATCTCAAGTCACCATCATGGAGGGATGGAGGCTCATGTCAGGGTGGAAAGCTGTGCAGAG
GAACCTCAGCTGAGGAGGAAGGCAGATGAGGAAGAGGACTATGATGATAACCTGTATGACTCAGATATGGATGT
GGTGAGGCTGGATGGAGATGATGTCAGCCCATTCATCCAGATCAGGTCAGTGGCTAAGAAACACCCTAAGACCTG
GGTCCACTACATTGCAGCTGAAGAGGAAGATTGGGACTATGCACCCCTGGTGCTGGCCCCAGATGATAGAAGTTA
CAAATCTCAGTATCTGAACAATGGGCCCCAGAGGATTGGAAGGAAGTACAAGAAAGTGAGGTTCATGGCTTATAC
TGATGAGACCTTTAAGACAAGAGAGGCAATCCAGCATGAAAGTGGCATCCTGGGACCACTGCTCTATGGAGAAGT
GGGGGATACCCTGCTCATCATCTTCAAGAACCAGGCCTCAAGGCCTTACAATATCTATCCCCATGGCATCACAGAT
GTGAGGCCTCTCTACAGCAGGAGACTGCCCAAGGGAGTCAAACACCTCAAGGATTTCCCCATCCTGCCAGGGGAA
ATCTTCAAGTATAAATGGACAGTCACTGTGGAAGATGGGCCAACTAAGTCAGATCCTAGGTGCCTGACCAGGTAC
TATTCTAGCTTTGTGAACATGGAGAGGGACCTGGCTTCAGGACTGATTGGACCTCTGCTCATCTGCTACAAAGAAT
CAGTGGACCAGAGGGGCAACCAGATCATGAGTGATAAGAGAAATGTCATCCTGTTCTCAGTGTTTGATGAGAATA
GGAGTTGGTATCTGACAGAAAACATCCAGAGGTTCCTGCCTAATCCTGCAGGAGTGCAGCTGGAGGACCCAGAAT
TTCAGGCTTCAAACATCATGCATAGTATCAATGGCTATGTGTTTGATAGTCTGCAGCTCTCTGTCTGCCTGCATGAG
GTGGCCTACTGGTATATCCTCAGCATTGGAGCTCAGACTGACTTCCTGAGTGTGTTCTTTTCAGGCTACACATTCAA
GCATAAGATGGTCTATGAAGATACCCTGACACTCTTCCCCTTTTCTGGGGAGACTGTGTTTATGAGCATGGAAAAC
CCAGGCCTGTGGATTCTGGGGTGCCACAACAGTGACTTCAGGAATAGAGGGATGACTGCTCTGCTCAAAGTGTCC
TCATGTGATAAGAATACTGGAGATTACTATGAGGACTCTTATGAAGATATCAGTGCATATCTGCTCTCCAAAAACA
ATGCCATTGAGCCCAGGTCATTTGCTCAGAACAGTAGACCACCTTCTGCAAGTGCACCAAAGCCTCCAGTGCTGAG
GAGACACCAGAGGGACATCAGCCTGCCAACCTTCCAGCCTGAGGAAGATAAAATGGACTATGATGATATCTTCTC
CACTGAGACCAAGGGGGAAGATTTTGACATCTATGGAGAGGATGAAAACCAGGACCCCAGGTCCTTCCAGAAGA
GGACCAGACACTACTTTATTGCAGCTGTGGAGCAGCTGTGGGACTATGGCATGTCTGAATCACCTAGAGCTCTGA
GGAACAGAGCACAGAATGGGGAGGTGCCCAGGTTCAAGAAAGTGGTGTTCAGAGAATTTGCAGATGGCTCTTTT
ACCCAGCCTAGCTACAGGGGGGAGCTCAACAAGCATCTGGGGCTGCTGGGACCCTATATCAGAGCAGAGGTGGA
AGATAACATCATGGTGACATTCAAGAATCAGGCCTCAAGACCCTACAGTTTTTATAGTTCTCTGATCAGCTACCCAG
ATGATCAGGAGCAGGGGGCTGAACCAAGGCACAACTTTGTGCAGCCTAATGAGACAAGAACTTACTTTTGGAAGG
TCCAGCATCACATGGCTCCCACAGAGGATGAGTTTGACTGCAAGGCCTGGGCATATTTTTCTGATGTGGACCTGGA
GAAGGATGTGCATAGTGGCCTCATTGGGCCACTGCTCATCTGCAGGGCAAACACACTGAATGCTGCACATGGCAG
GCAGGTCACTGTGCAGGAGTTTGCCCTGTTCTTTACAATCTTTGATGAAACTAAGTCCTGGTACTTCACAGAGAAT
GTGGAAAGGAATTGCAGAGCCCCCTGCCATCTCCAGATGGAGGACCCAACTCTGAAGGAAAACTACAGGTTCCAT

GCTATCAATGGATATGTCATGGATACACTGCCAGGCCTGGTGATGGCACAGAACCAGAGGATCAGGTGGTATCTG
CTCAGCATGGGGTCCAATGAGAATATCCATTCTATCCACTTCTCAGGACATGTCTTTTCAGTGAGGAAGAAAGAGG
AATATAAAATGGCTGTGTACAATCTGTATCCAGGGGTCTTTGAGACAGTGGAAATGCTGCCTAGCAAAGTGGGGA
TCTGGAGAATTGAGTGCCTCATTGGAGAACACCTGCAGGCAGGGATGTCCACCACATTTCTGGTGTACTCAAAGA
AATGCCAGACTCCCCTGGGGATGGCAAGTGGACATATCAGGGACTTCCAGATCACTGCATCAGGACAGTATGGAC
AGTGGGCACCAAAGCTGGCTAGGCTCCACTATAGTGGCTCTATCAATGCTTGGAGTACCAAAGAGCCTTTCTCTTG
GATCAAGGTGGATCTGCTGGCCCCCATGATCATCCATGGAATCAAAACACAGGGAGCTAGACAGAAGTTCAGCTC
CCTGTACATCAGTCAGTTTATCATCATGTATTCTCTGGATGGGAAGAAATGGCAGACCTACAGGGGCAATAGCACT
GGGACACTGATGGTCTTCTTTGGAAATGTGGATTCAAGTGGCATCAAGCACAACATCTTCAATCCTCCCATCATTGC
CAGGTACATCAGACTGCATCCCACACACTATTCAATCAGGAGTACTCTCAGAATGGAGCTGATGGGGTGTGACCTC
AACAGCTGCTCCATGCCACTGGGAATGGAATCCAAGGCAATCTCAGATGCCCAGATCACTGCTTCTAGCTACTTCA
CCAATATGTTTGCAACATGGTCACCCAGTAAAGCAAGGCTGCACCTCCAGGGAAGGTCCAATGCTTGGAGACCCC
AGGTGAACAATCCAAAGGAGTGGCTGCAGGTGGACTTTCAGAAAACCATGAAGGTCACAGGGGTGACTACCCAG
GGAGTGAAAAGTCTGCTCACCTCTATGTATGTCAAGGAGTTCCTGATCTCCTCAAGTCAGGATGGCCACCAGTGGA
CACTGTTCTTTCAGAATGGCAAGGTCAAAGTGTTCCAGGGGAATCAGGACAGCTTTACACCAGTGGTGAACAGCC
TGGACCCCCCTCTGCTCACTAGATATCTGAGAATCCATCCACAGAGCTGGGTGCACCAGATTGCACTCAGAATGGA
GGTCCTGGGCTGTGAAGCCCAGGACCTGTATTGA

<u>HSQ-LCO with CpGs</u>
ATGCAGATCGAACTGTCTACCTGTTTCTTTCTGTGCCTGCTGCGGTTTTGTTTTTCCGCT
ACCAGAAGATACTACCTGGGAGCCGTCGAACTGAGCTGGGATTACATGCAGTCTGACCTG
GGAGAGCTGCCCGTGGACGCTAGATTCCCACCTAGAGTCCCTAAGTCCTTCCCCTTCAAC
ACCAGCGTGGTCTACAAGAAAACCCTGTTCGTGGAGTTTACCGACCACCTGTTCAACATC
GCTAAGCCTAGACCACCATGGATGGGACTGCTGGGACCAACCATCCAGGCCGAGGTGTAC
GACACCGTGGTCATCACCCTGAAAAACATGGCTTCTCACCCCGTGTCCCTGCATGCTGTG
GGCGTCTCCTACTGGAAGGCCAGCGAAGGGGCTGAGTATGACGATCAGACCAGCCAGCGG
GAAAAAGAGGACGATAAGGTGTTCCCTGGCGGGTCCCATACCTACGTGTGGCAGGTCCTG
AAGGAGAATGGACCAATGGCTTCCGACCCTCTGTGCCTGACCTACTCTTATCTGTCCCAC
GTGGACCTGGTCAAGGATCTGAACAGCGGCCTGATCGGGGCTCTGCTGGTGTGTCGCGAA
GGGTCCCTGGCCAAGGAGAAAACCCAGACCCTGCATAAGTTCATCCTGCTGTTCGCCGTG
TTTGACGAAGGAAAAAGCTGGCACTCTGAGACCAAGAACTCTCTGATGCAGGACAGGGAT
GCCGCTTCCGCCAGAGCTTGGCCCAAGATGCACACCGTGAACGGCTACGTCAATAGGAGC
CTGCCTGGACTGATCGGCTGCCACAGAAAGTCCGTGTATTGGCATGTCATCGGAATGGGC
ACCACCCCTGAAGTGCACAGCATCTTCCTGGAGGGGCATACCTTTCTGGTCCGCAACCAC
CGGCAGGCTAGCCTGGAGATCTCTCCAATCACCTTCCTGACCGCCCAGACCCTGCTGATG
GACCTGGGACAGTTCCTGCTGTTTTGCCACATCTCCAGCCACCAGCATGATGGCATGGAG
GCTTACGTGAAAGTCGACTCCTGTCCCGAGGAACCTCAGCTGAGGATGAAGAACAATGAG
GAAGCCGAAGACTATGACGATGACCTGACCGACAGCGAGATGGATGTGGTCCGCTTCGAT
GACGATAACTCTCCCTCCTTTATCCAGATCCGGTCCGTGGCCAAGAAACACCCTAAGACC
TGGGTCCATTACATCGCCGCTGAGGAAGAGGACTGGGATTATGCTCCACTGGTGCTGGCC
CCCGACGATAGATCCTACAAAAGCCAGTATCTGAACAATGGACCCCAGAGGATCGGCAGA
AAGTACAAGAAAGTGAGGTTCATGGCTTATACCGATGAGACCTTTAAGACCAGAGAAGCC
ATCCAGCACGAGTCCGGGATCCTGGGACCTCTGCTGTACGGCGAAGTGGGGGACACCCTG
CTGATCATCTTCAAGAACCAGGCCAGCAGGCCTTACAATATCTATCCACATGGCATCACC
GATGTGAGACCTCTGTACTCCCGCCGGCTGCCAAAGGGCGTGAAACACCTGAAGGACTTC
CCAATCCTGCCCGGGGAAATCTTTAAGTATAAATGGACCGTCACCGTCGAGGATGGGCCC
ACCAAGAGCGACCCTAGGTGCCTGACCAGATACTATTCTTCCTTCGTGAATATGGAGAGA
GACCTGGCTTCCGGACTGATCGGACCCCTGCTGATCTGTTACAAAGAGAGCGTGGATCAG

CGCGGCAACCAGATCATGTCTGACAAGCGGAATGTGATCCTGTTCAGCGTCTTTGACGAA
AACCGCTCTTGGTACCTGACCGAGAACATCCAGCGGTTCCTGCCTAATCCAGCTGGAGTG
CAGCTGGAAGATCCCGAGTTCCAGGCCTCTAACATCATGCATTCCATCAATGGCTACGTG
TTCGACTCCCTGCAGCTGAGCGTGTGCCTGCACGAGGTCGCTTACTGGTATATCCTGAGC
ATCGGAGCCCAGACCGATTTCCTGTCTGTGTTCTTTTCCGGCTACACCTTTAAGCATAAA
ATGGTGTATGAGGACACCCTGACCCTGTTCCCATTTTCCGGCGAAACCGTGTTCATGAGC
ATGGAGAATCCCGGGCTGTGGATCCTGGGATGCCACAACTCCGATTTCAGGAATAGAGGG
ATGACCGCCCTGCTGAAAGTGAGCTCTTGTGACAAGAACACCGGAGACTACTATGAAGAT
AGCTACGAGGACATCTCTGCTTATCTGCTGTCCAAAAACAATGCCATCGAGCCCAGGAGC
TTCTCTCAGAACCCTCCAGTGCTGAAGCGCCACCAGCGGGAGATCACCAGAACCACCCTG
CAGAGCGATCAGGAAGAGATCGACTACGACGATACCATCTCCGTGGAAATGAAGAAAGAG
GACTTCGATATCTATGACGAAGATGAGAACCAGTCTCCCAGGTCCTTCCAGAAGAAAACC
AGACATTACTTTATCGCCGCTGTGGAGCGGCTGTGGGACTATGGCATGTCCAGCTCTCCT
CACGTGCTGAGAAATAGAGCTCAGTCCGGAAGCGTCCCACAGTTCAAGAAAGTGGTCTTC
CAGGAGTTTACCGACGGAAGCTTTACCCAGCCACTGTACCGCGGCGAACTGAACGAGCAC
CTGGGGCTGCTGGGACCCTATATCCGGGCTGAAGTGGAGGATAACATCATGGTCACCTTC
AGGAATCAGGCCAGCAGACCCTACTCTTTTTATTCCAGCCTGATCTCCTACGAAGAGGAC
CAGAGACAGGGAGCTGAACCAAGAAAAAACTTCGTGAAGCCTAATGAGACCAAAACCTAC
TTTTGGAAGGTGCAGCACCATATGGCCCCTACCAAAGACGAGTTCGATTGCAAGGCCTGG
GCTTATTTTAGCGACGTGGATCTGGAGAAGGACGTCCACTCCGGCCTGATCGGGCCACTG
CTGGTGTGTCATACCAACACCCTGAATCCAGCTCACGGAAGGCAGGTGACCGTCCAGGAA
TTCGCCCTGTTCTTTACCATCTTTGATGAGACCAAGAGCTGGTACTTCACCGAAAACATG
GAGAGGAATTGCAGAGCCCCATGTAACATCCAGATGGAAGACCCCACCTTCAAGGAGAAC
TACAGATTTCATGCTATCAATGGGTATATCATGGATACCCTGCCAGGACTGGTCATGGCT
CAGGACCAGAGGATCAGATGGTACCTGCTGAGCATGGGGTCTAACGAGAATATCCACTCC
ATCCATTTCAGCGGACACGTGTTTACCGTCCGCAAGAAAGAAGAGTACAAGATGGCCCTG
TACAACCTGTATCCCGGCGTGTTCGAAACCGTCGAGATGCTGCCTTCCAAGGCTGGGATC
TGGCGGGTGGAATGCCTGATCGGGGAGCACCTGCATGCCGGAATGTCTACCCTGTTCCTG
GTGTACTCCAATAAGTGTCAGACCCCCCTGGGGATGGCTAGCGGACATATCCGCGACTTC
CAGATCACCGCTTCCGGACAGTACGGACAGTGGGCTCCTAAGCTGGCTAGACTGCACTAT
TCTGGCTCCATCAACGCTTGGTCTACCAAAGAGCCTTTCTCCTGGATCAAGGTGGACCTG
CTGGCTCCAATGATCATCCATGGCATCAAAACCCAGGGGGCCAGGCAGAAGTTCTCTTCC
CTGTACATCAGCCAGTTTATCATCATGTATTCTCTGGATGGGAAGAAATGGCAGACCTAC
AGAGGCAATTCCACCGGGACCCTGATGGTGTTCTTTGGCAACGTCGACAGCTCTGGGATC
AAGCACAACATCTTCAATCCCCCTATCATCGCCCGCTACATCCGGCTGCACCCAACCCAT
TATTCCATCCGCAGCACCCTGCGGATGGAGCTGATGGGGTGCGATCTGAACAGCTGTTCT
ATGCCCCTGGGAATGGAGTCTAAGGCCATCTCCGACGCTCAGATCACCGCCTCCAGCTAC
TTCACCAATATGTTTGCTACCTGGTCCCCAAGCAAGGCTAGACTGCATCTGCAGGGAAGA
AGCAACGCTTGGAGACCACAGGTGAACAATCCCAAGGAGTGGCTGCAGGTCGACTTCCAG
AAAACCATGAAGGTGACCGGAGTCACCACCCAGGGCGTGAAAAGCCTGCTGACCTCTATG
TACGTCAAGGAGTTCCTGATCTCTTCCAGCCAGGACGGCACCAGTGGACCCTGTTCTTT
CAGAACGGAAAGGTGAAAGTCTTCCAGGGCAATCAGGATTCCTTTACCCCTGTGGTCAAC
AGCCTGGACCCACCCCTGCTGACCAGGTACCTGAGAATCCACCCACAGTCCTGGGTGCAT
CAGATCGCTCTGAGGATGGAAGTCCTGGGCTGCGAGGCCCAGGACCTGTATTGA

<u>HSQ-LCO CpGs removed</u>

ATGCAGATTGAACTGTCTACCTGTTTCTTTCTGTGCCTGCTGAGGTTTTGTTTTTCTGCT

ACCAGAAGATACTACCTGGGAGCTGTGGAACTGAGCTGGGATTACATGCAGTCTGACCTG
GGAGAGCTGCCTGTGGATGCTAGATTCCCACCTAGAGTCCCTAAGTCCTTCCCCTTCAAC
ACCTCTGTGGTCTACAAGAAAACCCTGTTTGTGGAGTTTACAGACCACCTGTTCAACATT
GCTAAGCCTAGACCACCATGGATGGGACTGCTGGGACCAACCATCCAGGCAGAGGTGTAT
GACACAGTGGTCATCACCCTGAAAAACATGGCTTCTCACCCTGTGTCCCTGCATGCTGTG
GGAGTCTCCTACTGGAAGGCCTCTGAAGGGGCTGAGTATGATGATCAGACCAGCCAGAGG
GAAAAAGAGGATGATAAGGTGTTCCCTGGAGGGTCCCATACCTATGTGTGGCAGGTCCTG
AAGGAGAATGGACCAATGGCTTCTGACCCTCTGTGCCTGACCTACTCTTATCTGTCCCAT
GTGGACCTGGTCAAGGATCTGAACTCTGGCCTGATTGGGGCTCTGCTGGTGTGTAGGGAA
GGGTCCCTGGCCAAGGAGAAAACCCAGACCCTGCATAAGTTCATCCTGCTGTTTGCTGTG
TTTGATGAAGGAAAAAGCTGGCACTCTGAGACCAAGAACTCTCTGATGCAGGACAGGGAT
GCTGCTTCTGCCAGAGCTTGGCCCAAGATGCACACAGTGAATGGCTATGTCAATAGGAGC
CTGCCTGGACTGATTGGCTGCCACAGAAAGTCTGTGTATTGGCATGTCATTGGAATGGGC
ACCACCCCTGAAGTGCACAGCATCTTCCTGGAGGGGCATACCTTTCTGGTCAGGAACCAC
AGGCAGGCTAGCCTGGAGATCTCTCCAATCACCTTCCTGACAGCCCAGACCCTGCTGATG
GACCTGGGACAGTTCCTGCTGTTTTGCCACATCTCCAGCCACCAGCATGATGGCATGGAG
GCTTATGTGAAAGTGGACTCCTGTCCTGAGGAACCTCAGCTGAGGATGAAGAACAATGAG
GAAGCTGAAGACTATGATGATGACCTGACAGACTCTGAGATGGATGTGGTCAGGTTTGAT
GATGATAACTCTCCCTCCTTTATCCAGATCAGGTCTGTGGCCAAGAAACACCCTAAGACC
TGGGTCCATTACATTGCTGCTGAGGAAGAGGACTGGGATTATGCTCCACTGGTGCTGGCC
CCTGATGATAGATCCTACAAAAGCCAGTATCTGAACAATGGACCCCAGAGGATTGGCAGA
AAGTACAAGAAAGTGAGGTTCATGGCTTATACAGATGAGACCTTTAAGACCAGAGAAGCC
ATCCAGCATGAGTCTGGGATCCTGGGACCTCTGCTGTATGGGGAAGTGGGGGACACCCTG
CTGATCATCTTCAAGAACCAGGCCAGCAGGCCTTACAATATCTATCCACATGGCATCACA
GATGTGAGACCTCTGTACTCCAGGAGGCTGCCAAAGGGGGTGAAACACCTGAAGGACTTC
CCAATCCTGCCTGGGGAAATCTTTAAGTATAAATGGACAGTCACAGTGGAGGATGGGCCC
ACCAAGTCTGACCCTAGGTGCCTGACCAGATACTATTCTTCCTTTGTGAATATGGAGAGA
GACCTGGCTTCTGGACTGATTGGACCCCTGCTGATCTGTTACAAAGAGTCTGTGGATCAG
AGGGGCAACCAGATCATGTCTGACAAGAGGAATGTGATCCTGTTCTCTGTCTTTGATGAA
AACAGGTCTTGGTACCTGACAGAGAACATCCAGAGGTTCCTGCCTAATCCAGCTGGAGTG
CAGCTGGAAGATCCTGAGTTCCAGGCCTCTAACATCATGCATTCCATCAATGGCTATGTG
TTTGACTCCCTGCAGCTGTCTGTGTGCCTGCATGAGGTGGCTTACTGGTATATCCTGAGC
ATTGGAGCCCAGACAGATTTCCTGTCTGTGTTCTTTTCTGGCTACACCTTTAAGCATAAA
ATGGTGTATGAGGACACCCTGACCCTGTTCCCATTTTCTGGAGAAACTGTGTTCATGAGC
ATGGAGAATCCTGGGCTGTGGATCCTGGGATGCCACAACTCTGATTTCAGGAATAGAGGG
ATGACAGCCCTGCTGAAAGTGAGCTCTTGTGACAAGAACACAGGAGACTACTATGAAGAT
AGCTATGAGGACATCTCTGCTTATCTGCTGTCCAAAAACAATGCCATTGAGCCCAGGAGC
TTCTCTCAGAACCCTCCAGTGCTGAAGAGGCACCAGAGGGAGATCACCAGAACCACCCTG
CAGTCTGATCAGGAAGAGATTGACTATGATGATACCATCTCTGTGGAAATGAAGAAAGAG
GACTTTGATATCTATGATGAAGATGAGAACCAGTCTCCCAGGTCCTTCCAGAAGAAAACC
AGACATTACTTTATTGCTGCTGTGGAGAGGCTGTGGGACTATGGCATGTCCAGCTCTCCT
CATGTGCTGAGAAATAGAGCTCAGTCTGGATCTGTCCCACAGTTCAAGAAAGTGGTCTTC
CAGGAGTTTACAGATGGAAGCTTTACCCAGCCACTGTACAGGGGAGAACTGAATGAGCAC
CTGGGGCTGCTGGGACCCTATATCAGGGCTGAAGTGGAGGATAACATCATGGTCACCTTC
AGGAATCAGGCCAGCAGACCCTACTCTTTTTATTCCAGCCTGATCTCCTATGAAGAGGAC
CAGAGACAGGGAGCTGAACCAAGAAAAAACTTTGTGAAGCCTAATGAGACCAAAACCTAC
TTTTGGAAGGTGCAGCACCATATGGCCCCTACCAAAGATGAGTTTGATTGCAAGGCCTGG
GCTTATTTTTCTGATGTGGATCTGGAGAAGGATGTCCACTCTGGCCTGATTGGGCCACTG

CTGGTGTGTCATACCAACACCCTGAATCCAGCTCATGGAAGGCAGGTGACAGTCCAGGAA
TTTGCCCTGTTCTTTACCATCTTTGATGAGACCAAGAGCTGGTACTTCACAGAAAACATG
GAGAGGAATTGCAGAGCCCCATGTAACATCCAGATGGAAGACCCCACCTTCAAGGAGAAC
TACAGATTTCATGCTATCAATGGGTATATCATGGATACCCTGCCAGGACTGGTCATGGCT
CAGGACCAGAGGATCAGATGGTACCTGCTGAGCATGGGGTCTAATGAGAATATCCACTCC
ATCCATTTCTCTGGACATGTGTTTACAGTAAGGAAGAAAGAAGAGTACAAGATGGCCCTG
TACAACCTGTATCCTGGGGTGTTTGAAACAGTGGAGATGCTGCCTTCCAAGGCTGGGATC
TGGAGGGTGGAATGCCTGATTGGGGAGCACCTGCATGCTGGAATGTCTACCCTGTTCCTG
GTGTACTCCAATAAGTGTCAGACCCCCCTGGGGATGGCTTCTGGACATATCAGGGACTTC
CAGATCACAGCTTCTGGACAGTATGGACAGTGGGCTCCTAAGCTGGCTAGACTGCACTAT
TCTGGCTCCATCAATGCTTGGTCTACCAAAGAGCCTTTCTCCTGGATCAAGGTGGACCTG
CTGGCTCCAATGATCATCCATGGCATCAAAACCCAGGGGGCCAGGCAGAAGTTCTCTTCC
CTGTACATCAGCCAGTTTATCATCATGTATTCTCTGGATGGGAAGAAATGGCAGACCTAC
AGAGGCAATTCCACAGGGACCCTGATGGTGTTCTTTGGCAATGTGGACAGCTCTGGGATC
AAGCACAACATCTTCAATCCCCCTATCATTGCCAGGTACATCAGACTGCACCCAACCCAT
TATTCCATCAGGAGCACCCTGAGAATGGAGCTGATGGGGTGTGATCTGAACAGCTGTTCT
ATGCCCCTGGGAATGGAGTCTAAGGCCATCTCTGATGCTCAGATCACAGCCTCCAGCTAC
TTCACCAATATGTTTGCTACCTGGTCCCCAAGCAAGGCTAGACTGCATCTGCAGGGAAGA
AGCAATGCTTGGAGACCACAGGTGAACAATCCCAAGGAGTGGCTGCAGGTGGACTTCCAG
AAAACCATGAAGGTGACAGGAGTCACCACCCAGGGAGTGAAAAGCCTGCTGACCTCTATG
TATGTCAAGGAGTTCCTGATCTCTTCCAGCCAGGATGGGCACCAGTGGACCCTGTTCTTT
CAGAATGGAAAGGTGAAAGTCTTCCAGGGCAATCAGGATTCCTTTACCCCTGTGGTCAAC
AGCCTGGACCCACCCCTGCTGACCAGGTACCTGAGAATCCACCCACAGTCCTGGGTGCAT
CAGATTGCTCTGAGGATGGAAGTCCTGGGCTGTGAGGCCCAGGACCTGTATTGA

<u>HSQ-NoCo</u>
ATGCAAATAGAGCTCTCCACCTGCTTCTTTCTGTGCCTTTTGCGATTCTGCTTTAGTGCCACCAGAAGATACTACCTG
GGTGCAGTGGAACTGTCATGGGACTATATGCAAAGTGATCTCGGTGAGCTGCCTGTGGACGCAAGATTTCCTCCT
AGAGTGCCAAAATCTTTTCCATTCAACACCTCAGTCGTGTACAAAAAGACTCTGTTTGTAGAATTCACGGACCACCT
TTTCAACATCGCTAAGCCAAGGCCACCCTGGATGGGTCTGCTAGGTCCTACCATCCAGGCTGAGGTTTATGATACA
GTGGTCATTACACTTAAGAACATGGCTTCCCATCCTGTCAGTCTTCATGCTGTTGGTGTATCCTACTGGAAAGCTTC
TGAGGGAGCTGAATATGATGATCAGACCAGTCAAAGGGAGAAAGAAGATGATAAAGTCTTCCCTGGTGGAAGCC
ATACATATGTCTGGCAGGTCCTGAAAGAGAATGGTCCAATGGCCTCTGACCCACTGTGCCTTACCTACTCATATCTT
TCTCATGTGGACCTGGTAAAAGACTTGAATTCAGGCCTCATTGGAGCCCTACTAGTATGTAGAGAAGGGAGTCTG
GCCAAGGAAAAGACACAGACCTTGCACAAATTTATACTACTTTTTGCTGTATTTGATGAAGGGAAAAGTTGGCACT
CAGAAACAAAGAACTCCTTGATGCAGGATAGGGATGCTGCATCTGCTCGGGCCTGGCCTAAAATGCACACAGTCA
ATGGTTATGTAAACAGGTCTCTGCCAGGTCTGATTGGATGCCACAGGAAATCAGTCTATTGGCATGTGATTGGAAT
GGGCACCACTCCTGAAGTGCACTCAATATTCCTCGAAGGTCACACATTTCTTGTGAGGAACCATCGCCAGGCGTCC
TTGGAAATCTCGCCAATAACTTTCCTTACTGCTCAAACACTCTTGATGGACCTTGGACAGTTTCTACTGTTTTGTCAT
ATCTCTTCCCACCAACATGATGGCATGGAAGCTTATGTCAAAGTAGACAGCTGTCCAGAGGAACCCCAACTACGAA
TGAAAAATAATGAAGAAGCGGAAGACTATGATGATGATCTTACTGATTCTGAAATGGATGTGGTCAGGTTTGATG
ATGACAACTCTCCTTCCTTTATCCAAATTCGCTCAGTTGCCAAGAAGCATCCTAAAACTTGGGTACATTACATTGCT
GCTGAAGAGGAGGACTGGGACTATGCTCCCTTAGTCCTCGCCCCCGATGACAGAAGTTATAAAAGTCAATATTTG
AACAATGGCCCTCAGCGGATTGGTAGGAAGTACAAAAAAGTCCGATTTATGGCATACACAGATGAAACCTTTAAG
ACGCGTGAAGCTATTCAGCATGAATCAGGAATCTTGGGACCTTTACTTTATGGGGAAGTTGGAGACACACTGTTG
ATTATATTTAAGAATCAAGCAAGCAGACCATATAACATCTACCCTCACGGAATCACTGATGTCCGTCCTTTGTATTC
AAGGAGATTACCAAAAGGTGTAAAACATTTGAAGGATTTTCCAATTCTGCCAGGAGAAATATTCAAATATAAATGG
ACAGTGACTGTAGAAGATGGGCCAACTAAATCAGATCCGCGGTGCCTGACCCGCTATTACTCTAGTTTCGTTAATA

TGGAGAGAGATCTAGCTTCAGGACTCATTGGCCCTCTCCTCATCTGCTACAAAGAATCTGTAGATCAAAGAGGAAA
CCAGATAATGTCAGACAAGAGGAATGTCATCCTGTTTTCTGTATTTGATGAGAACCGAAGCTGGTACCTCACAGAG
AATATACAACGCTTTCTCCCCAATCCAGCTGGAGTGCAGCTTGAGGATCCAGAGTTCCAAGCCTCCAACATCATGC
ACAGCATCAATGGCTATGTTTTTGATAGTTTGCAGTTGTCAGTTTGTTTGCATGAGGTGGCATACTGGTACATTCTA
AGCATTGGAGCACAGACTGACTTCCTTTCTGTCTTCTTCTCTGGATATACCTTCAAACACAAAATGGTCTATGAAGA
CACACTCACCCTATTCCCATTCTCAGGAGAAACTGTCTTCATGTCGATGGAAAACCCAGGTCTATGGATTCTGGGGT
GCCACAACTCAGACTTTCGGAACAGAGGCATGACCGCCTTACTGAAGGTTTCTAGTTGTGACAAGAACACTGGTG
ATTATTACGAGGACAGTTATGAAGATATTTCAGCATACTTGCTGAGTAAAAACAATGCCATTGAACCTAGGAGCTT
CTCTCAGAATCCACCAGTCTTGAAACGCCATCAACGGGAAATAACTCGTACTACTCTTCAGTCAGATCAAGAGGAA
ATTGACTATGATGATACCATATCAGTTGAAATGAAGAAGGAAGATTTTGACATTTATGATGAGGATGAAAATCAG
AGCCCCCGCAGCTTTCAAAAGAAAACACGACACTATTTTATTGCTGCAGTGGAGAGGCTCTGGGATTATGGGATG
AGTAGCTCCCCACATGTTCTAAGAAACAGGGCTCAGAGTGGCAGTGTCCCTCAGTTCAAGAAAGTTGTTTTCCAGG
AATTTACTGATGGCTCCTTTACTCAGCCCTTATACCGTGGAGAACTAAATGAACATTTGGGACTCCTGGGGCCATAT
ATAAGAGCAGAAGTTGAAGATAATATCATGGTAACTTTCAGAAATCAGGCCTCTCGTCCCTATTCCTTCTATTCTAG
CCTTATTTCTTATGAGGAAGATCAGAGGCAAGGAGCAGAACCTAGAAAAAACTTTGTCAAGCCTAATGAAACCAA
AACTTACTTTTGGAAAGTGCAACATCATATGGCACCCACTAAAGATGAGTTTGACTGCAAAGCCTGGGCTTATTTCT
CTGATGTTGACCTGGAAAAAGATGTGCACTCAGGCCTGATTGGACCCCTTCTGGTCTGCCACACTAACACACTGAA
CCCTGCTCATGGGAGACAAGTGACAGTACAGGAATTTGCTCTGTTTTTCACCATCTTTGATGAGACCAAAAGCTGG
TACTTCACTGAAAATATGGAAAGAAACTGCAGGGCTCCCTGCAATATCCAGATGGAAGATCCCACTTTTAAAGAGA
ATTATCGCTTCCATGCAATCAATGGCTACATAATGGATACACTACCTGGCTTAGTAATGGCTCAGGATCAAAGGAT
TCGATGGTATCTGCTCAGCATGGGCAGCAATGAAAACATCCATTCTATTCATTTCAGTGGACATGTGTTCACTGTAC
GAAAAAAAGAGGAGTATAAAATGGCACTGTACAATCTCTATCCAGGTGTTTTTGAGACAGTGGAAATGTTACCATC
CAAAGCTGGAATTTGGCGGGTGGAATGCCTTATTGGCGAGCATCTACATGCTGGGATGAGCACACTTTTTCTGGT
GTACAGCAATAAGTGTCAGACTCCCCTGGGAATGGCTTCTGGACACATTAGAGATTTTCAGATTACAGCTTCAGGA
CAATATGGACAGTGGGCCCCAAAGCTGGCCAGACTTCATTATTCCGGATCAATCAATGCCTGGAGCACCAAGGAG
CCCTTTTCTTGGATCAAGGTGGATCTGTTGGCACCAATGATTATTCACGGCATCAAGACCCAGGGTGCCCGTCAGA
AGTTCTCCAGCCTCTACATCTCTCAGTTTATCATCATGTATAGTCTTGATGGGAAGAAGTGGCAGACTTATCGAGGA
AATTCCACTGGAACCTTAATGGTCTTCTTTGGCAATGTGGATTCATCTGGGATAAAACACAATATTTTTAACCCTCC
AATTATTGCTCGATACATCCGTTTGCACCCAACTCATTATAGCATTCGCAGCACTCTTCGCATGGAGTTGATGGGCT
GTGATTTAAATAGTTGCAGCATGCCATTGGGAATGGAGAGTAAAGCAATATCAGATGCACAGATTACTGCTTCATC
CTACTTTACCAATATGTTTGCCACCTGGTCTCCTTCAAAAGCTCGACTTCACCTCCAAGGGAGGAGTAATGCCTGGA
GACCTCAGGTGAATAATCCAAAAGAGTGGCTGCAAGTGGACTTCCAGAAGACAATGAAAGTCACAGGAGTAACT
ACTCAGGGAGTAAAATCTCTGCTTACCAGCATGTATGTGAAGGAGTTCCTCATCTCCAGCAGTCAAGATGGCCATC
AGTGGACTCTCTTTTTTCAGAATGGCAAAGTAAAGGTTTTTCAGGGAAATCAAGACTCCTTCACACCTGTGGTGAA
CTCTCTAGACCCACCGTTACTGACTCGCTACCTTCGAATTCACCCCCAGAGTTGGGTGCACCAGATTGCCCTGAGGA
TGGAGGTTCTGGGCTGCGAGGCACAGGACCTCTACTGA

HSQ-MCO
ATGCAGATTGAGCTCAGCACCTGCTTCTTTCTGTGCCTGCTCAGGTTCTGCTTTTCAGCCACAAGGAGATACTATCT
GGGAGCTGTGGAACTGTCATGGGATTACATGCAGAGTGACCTGGGAGAGCTCCCTGTGGATGCTAGGTTCCCCCC
AAGGGTCCCAAAGTCTTTCCCTTTTAATACCAGTGTGGTCTATAAGAAAACACTCTTTGTGGAATTTACTGATCACC
TGTTCAACATTGCAAAGCCAAGGCCTCCCTGGATGGGACTGCTGGGACCTACCATCCAGGCTGAGGTGTATGACA
CTGTGGTCATCACACTGAAAAACATGGCATCTCACCCTGTCAGCCTGCATGCAGTGGGAGTCAGCTACTGGAAGG
CTTCAGAAGGGGCAGAGTATGATGATCAGACAAGCCAGAGAGAAAAAGAGGATGATAAGGTGTTCCCAGGAGG
GAGCCATACTTATGTGTGGCAGGTCCTGAAGGAGAATGGCCCAATGGCCAGTGACCCACTGTGCCTCACCTACTC
ATATCTGAGTCATGTGGACCTGGTCAAGGATCTCAACTCAGGCCTGATTGGGGCACTGCTGGTGTGCAGGGAAGG
CTCACTGGCCAAGGAGAAAACCCAGACACTGCATAAGTTCATCCTGCTCTTTGCTGTGTTTGATGAAGGGAAATCT
TGGCACAGTGAGACCAAGAACAGTCTGATGCAGGACAGGGATGCTGCTTCTGCCAGAGCTTGGCCCAAGATGCA

CACAGTGAATGGATATGTCAATAGGTCCCTGCCAGGACTCATTGGCTGCCACAGAAAGTCAGTGTATTGGCATGTC
ATTGGAATGGGCACCACACCAGAAGTGCACAGCATCTTCCTGGAGGGGCATACCTTTCTGGTCAGGAACCACAGG
CAGGCCAGCCTGGAGATCAGCCCAATCACCTTCCTGACAGCCCAGACTCTGCTCATGGATCTGGGGCAGTTCCTGC
TCTTTTGCCACATCAGCTCCCACCAGCATGATGGAATGGAGGCATATGTGAAAGTGGACTCCTGCCCAGAGGAAC
CACAGCTGAGGATGAAGAACAATGAGGAAGCTGAAGACTATGATGATGACCTGACAGACTCAGAGATGGATGTG
GTCAGGTTTGATGATGATAACAGCCCCTCCTTTATCCAGATCAGAAGTGTGGCCAAGAAACACCCAAAGACATGG
GTCCATTACATTGCAGCTGAGGAAGAGGACTGGGATTATGCACCTCTGGTGCTGGCCCCAGATGATAGATCCTAC
AAATCACAGTATCTGAACAATGGACCCCAGAGGATTGGCAGAAAGTACAAGAAAGTGAGGTTCATGGCCTATACT
GATGAAACATTTAAGACTAGAGAAGCTATCCAGCATGAGTCAGGCATCCTGGGACCACTGCTCTATGGAGAAGTG
GGGGACACCCTGCTCATCATCTTCAAGAACCAGGCTTCCAGGCCATACAATATCTATCCTCATGGCATCACAGATG
TGAGACCACTCTACTCAAGGAGACTGCCTAAGGGAGTCAAACACCTCAAGGACTTCCCTATCCTGCCAGGGGAAA
TCTTTAAGTATAAATGGACTGTGACAGTGGAGGATGGGCCCACTAAGAGTGACCCAAGGTGCCTGACCAGATACT
ATTCAAGTTTTGTGAATATGGAAAGGGATCTGGCATCAGGACTGATTGGACCTCTGCTCATCTGCTACAAAGAGAG
TGTGGATCAGAGGGGCAACCAGATCATGTCAGACAAGAGGAATGTGATCCTGTTCAGTGTCTTTGATGAAAACAG
GTCTTGGTATCTGACAGAGAACATCCAGAGATTCCTGCCAAATCCTGCAGGGGTGCAGCTGGAAGATCCAGAGTT
TCAGGCCTCAAACATCATGCATAGTATCAATGGATATGTGTTTGACAGTCTGCAGCTCTCTGTGTGCCTGCATGAA
GTGGCCTACTGGTATATCCTGTCCATTGGAGCTCAGACAGATTTCCTGAGTGTGTTCTTTTCAGGCTACACTTTTAA
GCATAAAATGGTCTATGAGGACACACTGACTCTCTTCCCTTTTAGTGGGGAAACAGTGTTTATGAGCATGGAGAAT
CCAGGGCTGTGGATTCTGGGATGCCACAACAGTGATTTCAGGAATAGAGGCATGACTGCTCTGCTCAAAGTGTCT
AGCTGTGACAAGAACACAGGGGACTACTATGAAGATTCTTATGAGGACATCAGTGCTTATCTGCTCTCCAAAAACA
ATGCAATTGAACCCAGATCATTCAGTCAGAATCCACCTGTGCTGAAGAGGCACCAGAGAGAGATCACTAGGACTA
CCCTGCAGTCAGATCAGGAAGAGATTGACTATGATGATACCATCTCAGTGGAAATGAAGAAAGAGGACTTTGATA
TCTATGATGAAGATGAGAACCAGAGTCCAAGGTCTTTCCAGAAGAAAACCAGACATTACTTTATTGCTGCAGTGGA
GAGGCTGTGGGATTATGGAATGTCCTCAAGTCCACATGTGCTGAGGAATAGGGCACAGTCTGGCAGTGTCCCTCA
GTTCAAGAAAGTGGTCTTCCAGGAGTTTACAGATGGCAGCTTCACTCAGCCTCTGTACAGGGGAGAACTCAATGA
GCACCTGGGGCTGCTGGGACCCTATATCAGAGCTGAAGTGGAGGATAACATCATGGTCACCTTCAGGAATCAGGC
TTCAAGACCCTACAGTTTTTATTCTAGCCTGATCAGCTATGAAGAGGACCAGAGGCAGGGAGCTGAACCTAGGAA
AAACTTTGTGAAGCCAAATGAGACCAAAACATACTTTTGGAAGGTCCAGCACCACATGGCACCAACCAAAGATGA
GTTTGATTGCAAGGCATGGGCCTATTTTTCAGATGTGGATCTGGAGAAGGATGTCCACAGTGGCCTCATTGGGCCT
CTGCTGGTGTGCCATACTAACACCCTGAATCCAGCTCATGGCAGGCAGGTGACAGTCCAGGAGTTTGCACTGTTCT
TTACCATCTTTGATGAGACAAAGTCCTGGTACTTCACTGAAAACATGGAGAGGAATTGCAGAGCTCCTTGCAACAT
CCAGATGGAAGACCCCACCTTCAAGGAGAACTACAGATTTCATGCAATCAATGGGTATATCATGGATACACTGCCA
GGACTGGTGATGGCCCAGGACCAGAGGATCAGATGGTATCTGCTCAGCATGGGGTCCAATGAGAATATCCACTCT
ATCCATTTCAGTGGACATGTGTTTACAGTCAGAAAGAAAGAAGAGTATAAAATGGCCCTGTACAACCTCTATCCAG
GAGTGTTTGAAACAGTGGAGATGCTGCCAAGCAAGGCTGGGATCTGGAGGGTGGAATGCCTCATTGGGGAGCAC
CTGCATGCAGGAATGTCAACCCTGTTTCTGGTCTACAGTAATAAGTGCCAGACACCTCTGGGAATGGCAAGTGGA
CATATCAGGGATTTCCAGATCACTGCTAGTGGACAGTATGGACAGTGGGCACCAAAGCTGGCTAGACTCCACTATT
CAGGCTCAATCAATGCTTGGTCCACCAAAGAGCCATTCTCATGGATCAAGGTGGACCTGCTGGCTCCTATGATCAT
CCATGGCATCAAAACACAGGGGGCAAGGCAGAAGTTCTCCTCACTGTACATCTCTCAGTTTATCATCATGTATAGC
CTGGATGGCAAGAAATGGCAGACCTACAGGGGCAATAGCACAGGGACTCTGATGGTGTTCTTTGGCAATGTGGA
CAGCAGTGGGATCAAGCACAACATCTTCAATCCCCCAATCATTGCAAGGTACATCAGACTGCACCCCACCCATTATT
CAATCAGGAGTACACTCAGGATGGAACTGATGGGGTGTGATCTCAACAGTTGCTCTATGCCACTGGGAATGGAGT
CCAAGGCAATCTCAGATGCCCAGATCACTGCTAGCTCCTACTTCACTAATATGTTTGCTACCTGGAGCCCCTCCAAA
GCAAGGCTGCACCTCCAGGGAAGGAGCAATGCATGGAGGCCTCAGGTGAACAATCCCAAGGAATGGCTGCAGGT
GGATTTCCAGAAAACTATGAAGGTGACTGGAGTCACAACTCAGGGAGTGAAAAGTCTGCTCACTTCTATGTATGTC
AAGGAGTTCCTGATCTCAAGTTCTCAGGATGGCCACCAGTGGACCCTGTTCTTTCAGAATGGAAAGGTGAAAGTCT
TCCAGGGCAATCAGGATTCCTTTACACCAGTGGTCAACTCACTGGACCCTCCCCTGCTCACTAGATATCTGAGAATC

CACCCTCAGAGCTGGGTGCATCAGATTGCTCTCAGAATGGAAGTCCTGGGCTGTGAGGCACAGGACCTGTATTGA
G

FIX sequences

<u>FIX-LCO</u>
ATGCAGAGGGTGAACATGATCATGGCTGAGTCTCCTGGACTGATCACCATCTGCCTGCTGGGCTATCTGCTGTCTG
CTGAGTGTACAGTGTTCCTGGACCATGAAAATGCTAATAAAATCCTGAACAGGCCAAAGAGGTACAATTCTGGGA
AACTGGAGGAATTTGTGCAGGGAAACCTGGAGAGGGAATGCATGGAGGAAAAGTGTAGCTTTGAGGAAGCCAG
GGAGGTGTTTGAAAATACAGAGAGGACCACAGAGTTCTGGAAACAGTATGTGGATGGGGATCAGTGTGAGTCCA
ACCCCTGTCTGAATGGAGGGTCTTGCAAGGATGATATCAACTCCTATGAGTGCTGGTGTCCTTTTGGATTTGAAGG
CAAGAATTGTGAGCTGGATGTGACCTGTAACATCAAAAATGGGAGGTGTGAGCAGTTCTGTAAGAACTCTGCTGA
TAATAAAGTGGTCTGCAGCTGTACAGAAGGCTACAGGCTGGCTGAGAACCAGAAGAGCTGTGAACCAGCTGTGC
CCTTCCCTTGTGGGAGGGTGTCTGTCAGCCAGACCAGCAAGCTGACCAGAGCTGAGACAGTGTTTCCTGATGTGG
ATTATGTCAACTCTACAGAGGCTGAAACCATCCTGGACAACATCACCCAGTCTACCCAGTCCTTCAATGACTTTACC
AGGGTGGTGGGAGGGGAGGATGCTAAGCCAGGACAGTTCCCCTGGCAGGTGGTCCTGAATGGCAAAGTGGATG
CTTTTTGTGGGGGCTCCATTGTGAATGAGAAGTGGATTGTCACAGCTGCTCACTGTGTGGAAACTGGGGTCAAGA
TCACAGTGGTGGCTGGAGAGCACAACATTGAGGAAACTGAACATACAGAGCAGAAAAGGAATGTGATCAGAATC
ATCCCCCACCATAACTACAATGCTGCTATCAACAAGTATAATCATGACATTGCCCTGCTGGAACTGGATGAGCCTCT
GGTGCTGAACAGCTATGTCACCCCAATCTGCATTGCTGACAAGGAGTATACCAATATCTTCCTGAAATTTGGGTCT
GGATATGTGTCTGGGTGGGGAAGGGTCTTCCACAAGGGAAGGTCTGCTCTGGTGCTGCAGTATCTGAGGGTGCC
CCTGGTGGACAGAGCTACCTGCCTGCTGAGCACCAAGTTCACCATCTACAACAATATGTTCTGTGCTGGATTTCAT
GAGGGAGGGAGGGACTCCTGTCAGGGAGATTCTGGAGGCCCTCATGTGACAGAGGTGGAAGGCACCAGCTTCCT
GACTGGCATCATCTCTTGGGGGGAGGAATGTGCTATGAAGGGGAAATATGGAATCTATACCAAGGTGTCCAGATA
TGTCAACTGGATCAAGGAGAAAACCAAGCTGACCTGA

<u>FIX-HCO</u>
ATGCAGAGGGTGAATATGATTATGGCTGAGTCCCCTGGGCTGATTACCATTTGCCTGCTGGGATACCTGCTGTCTG
CTGAGTGTACAGTGTTCCTGGACCATGAGAATGCAAATAAGATCCTGAACAGGCCCAAAAGATATAATAGTGGAA
AGCTGGAGGAATTTGTGCAGGGCAACCTGGAGAGAGAATGCATGGAGGAAAAGTGTAGCTTTGAGGAAGCCAG
GGAGGTGTTTGAAAATACAGAGAGAACCACAGAATTCTGGAAGCAGTATGTGGATGGAGATCAGTGTGAGAGCA
ACCCCTGTCTGAATGGAGGGAGTTGCAAAGATGATATCAACTCATATGAATGCTGGTGTCCTTTTGGATTTGAAGG
CAAAAATTGTGAGCTGGATGTGACCTGTAACATTAAGAATGGGAGGTGTGAGCAGTTTTGTAAAAACTCTGCTGA
TAATAAGGTGGTCTGCAGTTGTACAGAAGGGTATAGACTGGCTGAGAACCAGAAGTCCTGTGAACCAGCTGTGCC
CTTCCCTTGTGGAAGGGTGTCTGTCTCCCAGACTTCAAAACTGACCAGAGCTGAGACTGTGTTTCCTGATGTGGAT
TATGTCAACAGCACAGAGGCTGAAACTATCCTGGACAACATTACTCAGTCTACCCAGAGTTTCAATGACTTTACCA
GAGTGGTGGGAGGAGAGGATGCTAAACCAGGCCAGTTCCCCTGGCAGGTGGTCCTGAATGGGAAGGTGGATGC
ATTTTGTGGGGGATCTATTGTGAATGAGAAATGGATTGTCACAGCTGCTCACTGTGTGGAAACTGGGGTCAAGAT
CACAGTGGTGGCTGGAGAGCACAACATTGAGGAAACAGAACATACTGAGCAGAAGAGGAATGTGATCAGAATCA
TTCCTCACCATAACTACAATGCAGCCATCAACAAATATAATCATGACATTGCCCTGCTGGAACTGGATGAGCCTCTG
GTGCTGAACAGCTATGTCACACCAATCTGCATTGCTGACAAGGAGTACACTAACATCTTCCTGAAGTTTGGGTCAG
GATATGTGTCTGGATGGGGAAGAGTCTTCCACAAGGGCAGGTCTGCACTGGTGCTGCAGTATCTGAGAGTGCCTC
TGGTGGATAGGGCCACTTGTCTGCTGTCTACCAAGTTCACCATCTACAACAATATGTTCTGTGCTGGATTTCATGAG
GGAGGGAGAGACTCCTGTCAGGGAGATTCTGGAGGCCCACATGTGACAGAGGTGGAAGGCACCAGCTTCCTGAC
AGGCATCATTTCCTGGGGGGAGGAATGTGCAATGAAGGGGAAATATGGAATCTACACCAAAGTGAGCAGGTATG
TGAACTGGATCAAGGAAAAGACCAAACTGACATGA

6x tRNA sequence

6x-tRNA
GTCGACTACGTAGAATCCGGAGCCGTCTTTGTCTTCCAGCTCCATCTTTTCCACCTTTTGCTTAGGCAGTCCCCCGA
GTCGTGTCAAGGCTGAGGAGTAGAAATGGAACAGCACTAATATTAATGGCAAAACCGTTGTGAAATAGGGTTACT
TTCTGTTTAAGCAAGGAAAATAAAGTAAAGCAATGGGAAAAAAATTAAAAGCAAAAGGAATGGAGGTGCCGGGG
ATTGAACCCGGGGCCTCGTGCATGCTAAGCACGCGCTCTACCACTGAGCTACACCCCCGTACTGAAACGGTTCTCT
CGAGAGTATATTCAAGATCAGAATCTGACCCTTTTGCTAGGTTTCAGAACCATTAGTTGTAATCAGCCAAGGTCTAT
TTTATTTAGTTATTTCTGATATCTCAAATTTAGGTTTTGCGTCCCTCTTTGCTGACAGCTGAGCAAACCGCATTCTAC
ACCGAAGGCCCTCTATTGATGGCCCTGATTTAAATCCCTTCCGCCGCCTGCCGCAGGTGGCTAGGGTCTGAGCACA
CTTGAACCTCACACCCGCCCCAGGGGTAGCTCCTTGGTTCCCTTCAGCCCCGATGTGTCCCTCGTGCTTTGAAATGG
AATTACAGTTTTGGTTAAAAACATGCCTTTTCCGAGTTAGGAAGAATCTAAATCGACTGAACGCCAGTCTAAAATTT
CGGCGTTCCCACACCGGGAGTCGAACCCGGGCCGCCTGGGTGAAAACCAGGAATCCTAACCGCTAGACCATGTG
GGAGACGGCAATAGCGACTCCAAGCCTAGACAAATTGAGTCTTCTCGGTCGGCTTCCGCCCACTCCATCGCGTTCA
TCCGTAGGCGTCAAACCTGCTCCTGCGCCTGCGCGGAGTCTGCAGCGGTTTAAACCGTTCAGGTTCGCATTCTACT
GTTTCTCTCCTTGCAGGGGCCCTTGAATCTTTCTTCAATCTACTCTCGCGTGCCCGGGCGACTGGGCATAACCCTAC
AGGTTCATGTGGGGTGGGTGGCGCGCGCTAGCGGTGAAGGTCACTCACAATTGCGCGCTGGGCAGACGACGGCA
GCCATTACTTTTACCTCGATCGCTGTTTTCCTGGATCCGCACGGGTCCAACCCGACTCATCCCAACCAACCTGAGGT
ATGAAAACCAGGAAAGAGAGCTAGCACCGGAGCGTTGGTGGTATAGTGGTAAGCATAGCTGCCTTCCAAGCAGT
TGACCCGGGTTCGATTCCCGGCCAACGCAAGTCGTTTTGGGTGTTTTTTCCCCCCCCCGCCTTTTCCTTTTCGTGTTT
TCTGGGCCCCAGCATCGTTGAGGGTTTTCGTGAGGTTTTCCTGAGGAAACTTCCGCTCCGAAAGGACCCACTTTCC
GCTACACCCGCGACCACGGCTGGACCACCGCGCTCCTGACGGATGCGCCCTGCAAGCCCCTCCAGGCGAGAGCAG
GCCGGCCTGTGCTCAGTTTTGTAGCATCAAAACTAGGATTTCTCTTGTTACCCCCAGTCACTCCATTCAGTTTTCGTG
TCTTTCCCAGCTGCATCCATCCTTTCCTCATTTTCGTATGCAGCCGACTTTTTGTGACATCTTTGTATTCATTCTCTGC
AATTCAGCTGACCTGGCCAAGGAAACAAGATCCTAAGCGTCTTTCCGGCGGCGCCGTGGCTTAGTTGGTTAAAGC
GCCTGTCTAGTAAACAGGAGATCCTGGGTTCGAATCCCAGCGGTGCCTCCGTGTTTCCCCCACGCTTTTGCCAACAT
TAAACATTGTGAGGACAGTTGCAGAAACTCATAACTTCCATCCTACATGGTTTACTCACGTACCCATCTATCCTCTCC
CGGTGCATCTGCCACACGCTGTTGGGTTTTTGCTCTTCGTGCACATGGTACTTGCGCCTCGACCTGCAGTTACACCA
GTCGCATCATCTGTACAGCGCTAAAACCTAGCTGGGCGTGGTGGTCCTGCAGTCCCAGCTACTCGGGAGGCTGAG
GCAGGAGAATGGCTTGAATCCAGGAGGCGGAGGTGGCAGTGAGCCGAGATTGCGCCACTGCACTCCAGCCTGGT
GACAGAGCGAGACTCCATCTCAAAAAAAAAAAAAAAAAAAAAAGTCAGAATTAGGTACTAAAAGCCATATGACATG
CCTGAACAGGGACTTGAACCCTGGACCCTCAGATTAAAAGTCTGATGCTCTACCAACTGAGCTATCCAGGCTTCTT
CCCTGCTAGTTTATTTTAATGCAGTAATAAATAACAGCACTTTGTTAAAAATAATAAAGGTATAATCTGTGACACAT
CCAAAGTGGACAAGATGAAGAGATAATAGGTATACACCAGGATTCCCCTGGGCAAACTGGGACCTCTTGGTACCC
TATATATTAAGAGTCTCGGGTTTTGTTTTCACTTAAGCAAATGGTTAACGAATTAGCAGGTTAAGAAAAACTGTTTC
CCCGTAAGAAGCAGGGTTCTTTGGTGTTCAATGTGGAGCTCCGCCACTCCCAGCCCGGGTGAAGGAAAACTGGGA
AACAGAATGAATGTGATTATCTATTCGAAGATAAATTTCCACAAAGCATGCCGTTTGATAGTAGCTTATAATGTGG
AAGTAAGGCATCCTGTCATCCGGCCGGTTAGCTCAGTTGGTTAGAGCGTGGTGCTAATAACGCCAAGGTCGCGGG
TTCGATCCCCGTACTGGCCAAGTATTCTCTGTGGCTTTTATCACCAGAATGGATAGTAACCCAGACATCGATCTAAA
CGTGTACCTGTGTGTTTCTCCAGGCTTAACTTTGCCCCGAGAAAACGGATCTGTGAATTTGGTGCGCCCTCGCTTAC
TCGACAGCGGTTAATTTGAACGGGGACGTTTCTTTCCGCTGCCTCCAAGGCATACCCACATCCTACCACGATGGTG
GCGGCCGCAA

Supplementary Figure 2

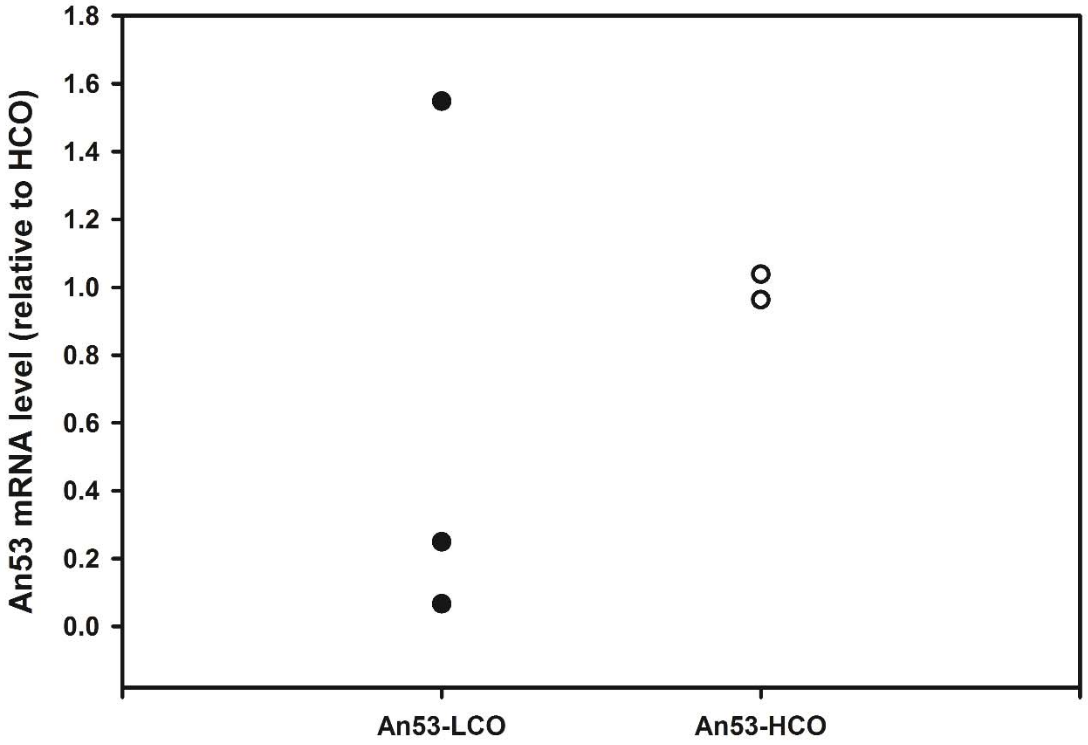

***In vivo* HCO and LCO An53 mRNA expression:** mRNA was extracted from the livers of mice dosed with AAV-HLP-An53-LCO and AAV-HLP-An53-HCO and relative levels of An53 mRNA was determined by quantitative PCR.
