# Supplementary material for: Target-Cell-Directed Bioengineering Approaches for Gene Therapy of Hemophilia A
Source: Mol Ther Methods Clin Dev. 2018 Jan 31;9:57–69. doi: 10.1016/j.omtm.2018.01.004 (PMC5852392; doi:10.1016/j.omtm.2018.01.004)
Supplement: Document S2. Article plus Supplemental Information [file mmc2.pdf]

Molecular Therapy
Methods & Clinical Development
Original Article

AMERICAN SOCIETY of GENE & CELL THERAPY

# Target-Cell-Directed Bioengineering Approaches for Gene Therapy of Hemophilia A

Harrison C. Brown,[1] Philip M. Zakas,[1] Stephan N. George,[2] Ernest T. Parker,[2] H. Trent Spencer,[2] and Christopher B. Doering[2]

[1]Graduate Program in Molecular and Systems Pharmacology, Laney Graduate School, Emory University, Atlanta, GA, USA; [2]Department of Pediatrics and Aflac Cancer and Blood Disorders Center, Emory University School of Medicine, Atlanta, GA, USA

**Potency is a key optimization parameter for hemophilia A gene therapy product candidates. Optimization strategies include promoter engineering to increase transcription, codon optimization of mRNA to improve translation, and amino-acid substitution to promote secretion. Herein, we describe both rational and empirical design approaches to the development of a minimally sized, highly potent AAV-fVIII vector that incorporates three unique elements: a liver-directed 146-nt transcription regulatory module, a target-cell-specific codon optimization algorithm, and a high-expression bioengineered fVIII variant. The minimal synthetic promoter allows for the smallest AAV-fVIII vector genome known at 4,832 nt, while the tissue-directed codon optimization strategy facilitates increased fVIII transgene product expression in target cell types, e.g., hepatocytes, over traditional genome-level codon optimization strategies. As a tertiary approach, we incorporated ancient and orthologous fVIII sequence elements previously shown to facilitate improved biosynthesis through post-translational mechanisms. Together, these technologies contribute to an AAV-fVIII vector that confers sustained, curative levels of fVIII at a minimal dose in hemophilia A mice. Moreover, the first two technologies should be generalizable to all liver-directed gene therapy vector designs.**

## INTRODUCTION

From a pharmacological perspective, gene-transfer-based therapies possess a unique set of challenges. Instead of focusing on absorption, distribution, metabolism, and excretion, typical preclinical and clinical gene therapy safety and efficacy studies assess (1) transgene product levels in plasma or cells, (2) immune responses to both the vector and transgene product, (3) risk of insertional mutagenesis, and (4) the potential for vertical gene transmission. Conceptually, gene addition strategies for monogenic diseases tend to be straightforward, with a goal of providing therapeutic levels of a functional version of the missing or defective disease-causing protein. Since the biology of the transgene product often is well understood from molecular genetic and biochemical studies, current gene therapy research and development efforts tend to be directed toward maximizing product potency and manufacturability. These critical aspects help define practical dose ranges that often are limited by both vector-related toxicity/immune responses as well as vector supply.

Hemophilia A is an X-linked bleeding disorder caused by a deficiency in coagulation factor VIII (fVIII). A growing body of clinical experience supports the use of liver-directed recombinant adeno-associated virus (rAAV) as a gene therapy vector for the treatment of hemophilia B and, more recently, hemophilia A.[1–5] Two primary obstacles have slowed AAV-fVIII vector development for hemophilia A compared to similar AAV-factor IX (fIX) vectors designed for hemophilia B. These obstacles include the limited DNA packaging capacity of the AAV for the large fVIII transgene size[6–8] and the inefficient biosynthesis of human fVIII transgene products in heterologous target cells.[9] Clinical experience with liver-directed rAAV serotype 8 (rAAV8)-fIX has shown that, at vector doses of approximately $2 \times 10^{12}$ vector genomes (vg) per kilogram (kg), capsid-mediated cellular immunity directed toward rAAV8-modified hepatocytes results in transient acute liver toxicity and decreases in fIX levels without steroid intervention.[2,4,10]

While the precise packaging capacity of rAAV is debated, it is accepted that vg sizes of 4.7–4.9 kb result in efficient transgene packaging and expression.[6–8] Due to the large transgene size of B-domain-deleted (BDD) human fVIII (hfVIII) at 4.4 kb and the requirement for additional non-coding viral and gene expression regulatory control elements, rAAV-fVIII vectors routinely exceed this ideal vg length, resulting in suboptimal transgene packaging and delivery.[6,11,12] An rAAV-fVIII genome minimally contains a promoter, the fVIII transgene, a poly(A) polyadenylation signal, and rAAV inverted terminal repeats (ITRs) flanking both sides of the cassette. Combined, the immutable elements, the ITRs, and the fVIII transgene, occupy 4,664 bp of the 4,900 bp available, leaving only 246 bp for the promoter, poly(A) signal, and any intervening sequences. Currently utilized liver-specific promoters, such as the hybrid liver promoter (HLP) and hepatic control region (HCR)-human α-1 antitrypsin (hAAT) promoter, range in size from 250 nt to over 700 nt in length, which alone exceeds the remaining capacity of the rAAV vector.[3,12] To alleviate this limitation, we utilized a combinatorial process of

Received 3 November 2017; accepted 9 January 2018;
https://doi.org/10.1016/j.omtm.2018.01.004.

**Correspondence:** Christopher B. Doering, Department of Pediatrics, Emory University School of Medicine, 2015 Uppergate Dr. NE, Room 450, Atlanta, GA 30322, USA.
**E-mail:** cdoerin@emory.edu


This is an open access article under the CC BY license (http://creativecommons.org/licenses/by/4.0/).

assembling and testing promoter and enhancer fragments to identify minimal synthetic promoters that drive strong gene expression in hepatocytes.

An additional factor that limits the efficacy of rAAV-fVIII vectors is the low biosynthetic efficiency of the fVIII protein itself. fVIII is a large glycoprotein that is secreted at levels 2–3 orders of magnitude less efficiently than similarly sized glycoproteins.[9] We have previously described a chimeric human/porcine fVIII molecule designated ET3 (originally designated HP47) that demonstrates 10-fold or greater increased secretion efficiency compared to that of BDD hfVIII (designated HSQ).[13–18] More recently, ancestral sequence reconstruction of ancient mammalian fVIII molecules was performed. One construct, designated An53, was identified that, despite sharing 95% identity with HSQ, expresses at high levels similar to or exceeding those of ET3.[19]

All rAAV-fIX and rAAV-fVIII product candidates currently in clinical testing incorporate codon-optimized transgenes. Traditional codon optimization strategies utilize the entire genomic cDNA of a host species to derive the codon usage bias (CUB) of the organism as a whole, which is assumed to be reflective of the individual tRNA concentrations within its cells, originally described as the "tRNA adaptation theory."[20–23] However, recent work, additionally, has shown that individual tRNA concentrations vary between tissues and cell types.[24] Therefore, depending on the target tissue or cell type desired for transgene expression, the assumed prevalence of individual tRNA species derived from the host organism's genomic cDNA may not be reflective of the tissue/cell tRNA concentrations. Herein, we examined tissue/cell-type-specific CUB tables for codon optimization in the liver-directed AAV gene therapy setting. Specifically, we sought to further improve the expression of ET3, HSQ, and An53 using this novel codon optimization strategy. By combining minimal synthetic target cell-directed promoters and target cell-directed codon optimization, a 4.88-kb AAV8-BDDhfVIII vector and a slightly larger AAV8-ET3 vector were created and tested in cell lines and a murine model of hemophilia A.

## RESULTS

### Synthetic Promoter Construction

Ryffel et al. previously reported that a 41-bp fragment from the *Xenopus laevis* albumin 5′ UTR is, by itself, sufficient to drive gene transcription *in vitro*.[25] This 41-bp region, designated SynO, contains the HP1 hepatocyte-specific transcription factor (TF) binding site as well as a TATA box. Similarly, Godbout et al. reported a 61-bp fragment immediately 5′ of the murine α-fetoprotein gene (AFP) as also sufficient to drive transcription.[26] When inserted into a plasmid cassette encoding a GFP transgene, these elements alone failed to generate detectable GFP fluorescence in transfected HepG2 cells (data not shown). To increase the transcriptional power of these minimal promoters, an iterative process of modification was utilized, wherein a 5′ flanking fragment of the human α-microglobulin/Bikunin precursor gene enriched with liver-specific TF binding sites, designated Abp, was fused to the 5′ ends of both SynO and AFP promoters. The AFP promoter, additionally, was augmented with an HP1 TF binding site. Two Abp fragment variants were designed, one of which included 94 bp of native genomic sequence (AbpNat), and the other was a 61-bp variant wherein non-TF binding regions, as identified by Rouet et al., were deleted and replaced by 3-bp segments of randomly generated DNA sequence (designated AbpShort).[27] These promoters were further modified with the inclusion of non-native transcription factor binding sites and a consensus transcription start site (TSS) (Figure 1A).

The hybrid promoters were inserted into a cassette driving the expression of a bioengineered fVIII variant ET3, which previously has been shown to possess increased secretion efficiency.[13–16,18,19,28] The relative strength of these promoters was compared to that of the previously described 252-bp HLP, which represents one of the shortest yet strongest liver-directed promoters described to date.[12] When transfected into HepG2 cells, the AbpNat element fused to both SynO and AFP promoters drove detectable levels of fVIII expression, while the reduction of AbpNat to AbpShort did not impair gene expression. Inclusion of the TSS dramatically increased fVIII expression to levels significantly higher than that of the HLP, while addition of the non-native TF binding sites HNF1 or SP1 in the context of the AbpNat enhancer diminished fVIII expression to undetectable levels (Figure 1B).

Based on these data, five synthetic promoters were chosen for *in vivo* testing by hydrodynamic injection into hemophilia A mice (Figure 1C). Both the AbpNat-AFP and AbpNat-SynO designs drove detectable fVIII expression yet did not achieve the strength of the HLP promoter. In agreement with the *in vitro* data, inclusion of the TSS motif to AbpNat-HP1-AFP dramatically increased fVIII expression, achieving fVIII activity levels 4-fold higher than that of the HLP. AbpShort coupled with either HP1-AFP-TSS or HNF1-SynO-TSS permitted the highest *in vivo* fVIII expression of all plasmids tested. Due to its small size of only 146 bp, and strong *in vivo* activity 14-fold that of HLP, HNF1-AbpShort-SynO-TSS was chosen as the lead candidate promoter design for our final AAV-fVIII vector. For convenience, this synthetic promoter was renamed the hepatic combinatorial bundle (HCB). The sequences of all promoters described herein are given in the Supplemental Information.

### Tissue-Specific Codon Optimization Index

Previous work by Dittmar et al. revealed that the CUB of genes highly and specifically expressed in human liver differ from the CUB of the human genome coding DNA sequence (CDS).[24] Furthermore, liver CUB was uniquely correlated with liver tRNA content. Using a list of the top 43 most highly and specifically expressed liver mRNA, a CUB table was constructed. Building off these data, we utilized the liver CUB table to synthesize liver codon-optimized (LCO) variants of HSQ, ET3, and the recently described ancestral fVIII protein, An53.[19] In order to test the specificity of LCO, a similar strategy was utilized to construct a CUB table based on mRNAs highly and specifically expressed in myeloid cells of the blood compartment. This myeloid codon optimization (MCO) strategy was applied to

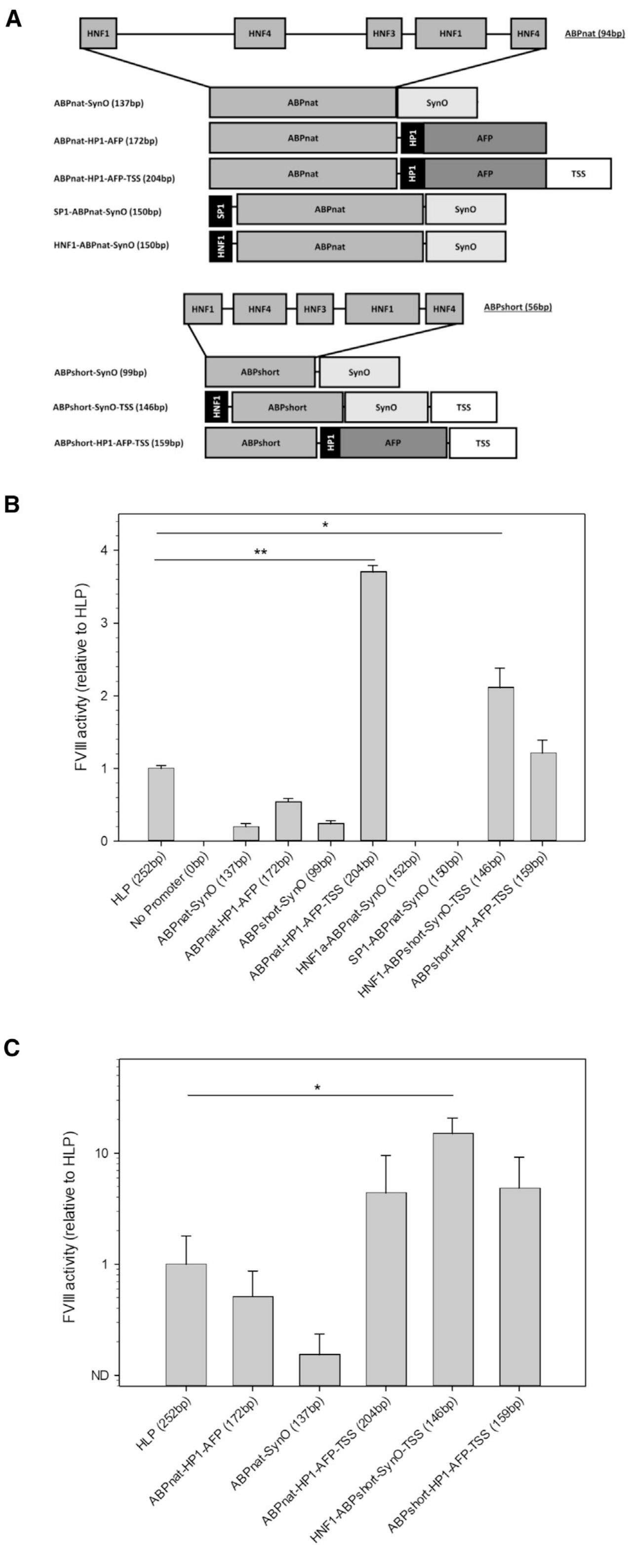


**Figure 1. Promoter Design and Testing**

(A) Novel, liver-directed synthetic promoters were constructed by the assembly of discrete units designed for high transcriptional activity in the context of the liver. Straight lines represent DNA of no known function. HNF, hepatocyte nuclear factor; AbpNat, α-microglobulin/bikunin precursor native sequence; AbpShort, human α-microglobulin/bikunin precursor shortened sequence; AFP, α-fetoprotein; TSS, transcription start site. (B) To determine the relative strength of promoter designs, promoters driving the expression of ET3 were transfected into HepG2 cells, and fVIII activity was measured 48 hr post-transfection. Activity is displayed relative to HLP (*p < 0.001 by one-way ANOVA and the Holm-Sidak method). (C) To determine the relative strength of the promoters *in vivo*, plasmid DNA was delivered by hydrodynamic injection to hemophilia A mice, and plasma fVIII activity was measured by fVIII chromogenic assay (n = 3–4 per group; *p < 0.001 by one-way ANOVA and the Holm-Sidak method). Data are presented as the mean ± sample SD.

both HSQ and ET3, while standard human codon optimization (HCO) was also applied to An53. *In silico* codon optimization was performed utilizing a commercial algorithm with the custom liver and myeloid CUB tables utilized in place of standard species-specific, genome-based CUB tables. Importantly, all other optimization parameters, including removal of *cis*-acting motifs, destabilizing RNA structures, GC content, etc., were performed equally across all optimizations. Additionally, non-codon-optimized (NoCo) versions of HSQ and ET3, which contain native human and hybrid human/porcine *F8* cDNA sequences, respectively, were used as additional controls.

We first sought to quantify the deviation of the LCO and MCO CUB tables from the standard human CUB table. The CUB of an individual codon species within a coding sequence is given by formula 1:

$$CUB_i = \frac{C_i}{C_{aa}}, \text{ where } \sum_{i=1}^{n} CUB_i = 1$$

and $n$ is the number of the synonymous codons for an individual amino acid.

The CUB of an individual codon species $(CUB_i)$ is given by the ratio of the number of times a single codon $(C_i)$ is utilized to the total number of times all codons $(C_{aa})$ coding for the corresponding amino acid (identical + synonymous) are utilized within a coding sequence. The deviation from human CUB is given in formula 2:

$$FD = \frac{CUBir}{CUBih}.$$

$CUB_{ir}$ is the CUB of a single codon within the reference CUB table, and $CUB_{ih}$ is the CUB of the respective individual codon in the human CUB table. FD is the fold difference observed for each $CUB_{ir}$ relative to the equivalent $CUB_{ih}$.

For comparison, the deviation of the murine CUB table against the human CUB table was quantified. With respect to the human CUB table, the average $CUB_i$ FD of the liver and myeloid CUB tables was substantially greater than that of the murine CUB table (Figure 2A). On average, murine FDs deviated from the human bias by just 1.04-fold per codon, whereas the liver and myeloid FDs showed

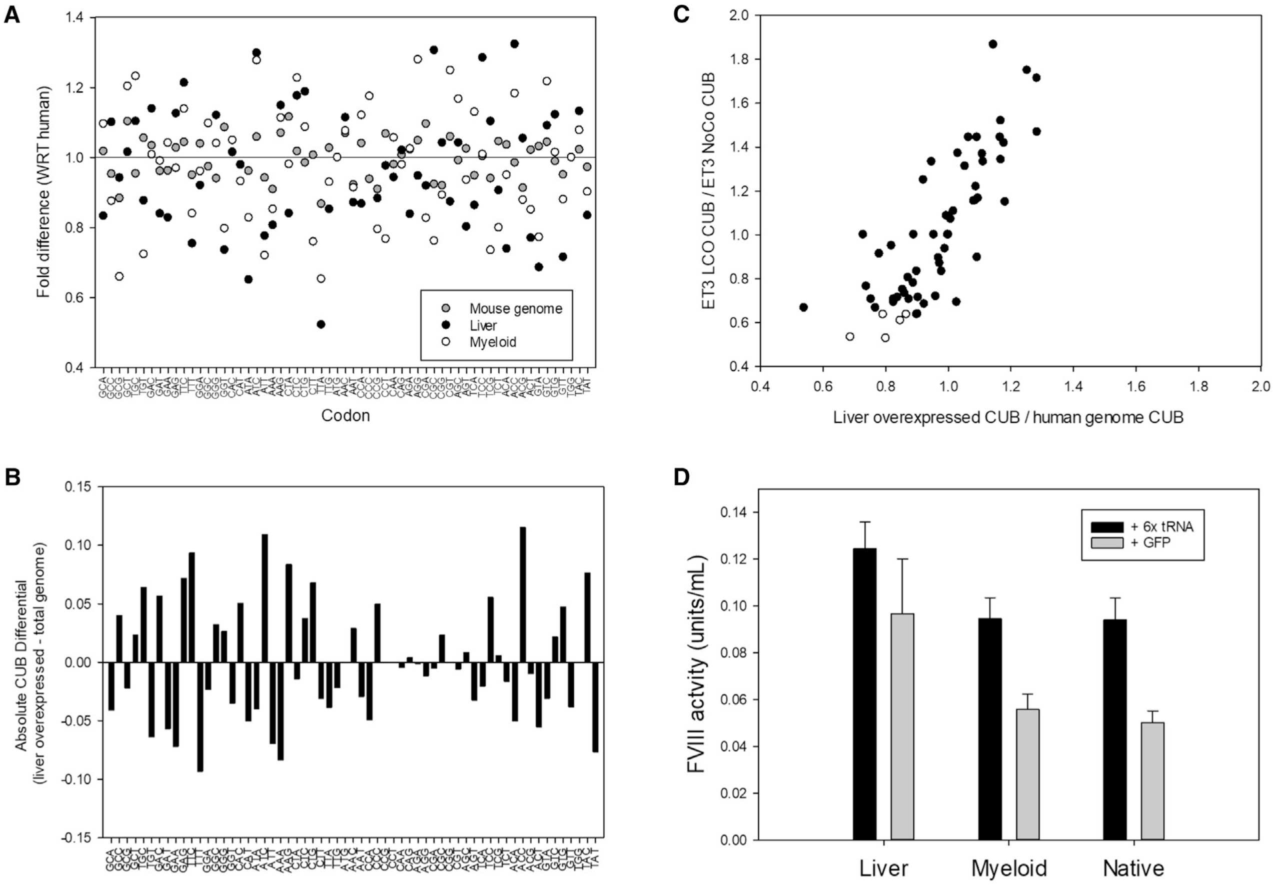


**Figure 2. Liver-Directed Codon Optimization**

(A) The fold difference in CUB of each codon in the liver CUB table with respect to the same codon in the human CUB table, as defined in formula 2. (B) The absolute difference codon usage bias of each codon in the liver CUB table versus the respective codon in the human CUB table, as defined by formula 3. (C) The correlation of the ratio of CUB for the set of liver genes to that of the entire human genomic coding sequence against the correlation of the ratio of CUB for ET3-LCO to that of ET3-NoCo. Points represent individual codon species. Open circles denote the 6 codon species with the lowest ratio of ET3-LCO to ET3-NoCo. Due to overlapping positions, two open circles appear as a single point. (D) Plasmid DNA encoding codon-optimized variants of ET3 were co-transfected into HepG2 cells with either GFP expression plasmid or a plasmid encoding the six tRNAs predicted to be most limiting of the expression of ET3-NoCo (*p < 0.05 by two-tailed t test).

greater than 3 times more deviation, with an average of 1.15- and 1.14-fold difference per codon, respectively. While this analysis demonstrates the relative deviations contained within the human liver, human myeloid, and murine genome CDS CUB to human CDS CUB in relative terms, the absolute difference (AD) in magnitude of CUB can also be considered and is presented as formula 3.

Formula 3: $AD_h = \left| CUB_{ip} - CUB_{ih} \right|$.

$AD_h$ is the absolute difference in magnitude of the $CUB_i$ of a single codon species from an individual protein ($CUB_{ip}$) from that of the respective codon in the human CUB table ($CUB_{ih}$). The $AD_h$ of each codon species was calculated for the list of 43 hepatocyte-specific genes/proteins, as well as for each individual gene/protein in the human CDS.

The mean $AD_h$ of $CUB_{ip}$ calculated for each of the 43 proteins from which the LCO CUB table deviated by as much as 0.14 (ATC: liver, 0.61; human, 0.47), with the mean of the $AD_h$ of each codon species of the proteins from which the LCO CUB was generated being significantly greater than the mean $AD_h$ of the human CDS in 22 codons (Figure 2B). Together, these data support the concept that codon usage within genes highly and uniquely expressed by specific tissue(s) differs from that of the human CDS as a whole and that a higher degree of variation may exist between tissues of a single organism than between orthologous species.

We next analyzed how closely the process of *in silico* liver codon optimization of ET3 mimicked the naturally occurring codon biasing of the genes endogenously overexpressed within the liver (Figure 2C). Mapping the correlation of the ratio of CUB for the set of highly expressed liver genes to that of the entire human CDS against the ratios of CUB of ET3 LCO to that of non-codon optimized (NoCo) ET3, which utilizes native human and porcine cDNA sequences, revealed a correlation across all codons (r = 0.81). Effectively, this shows

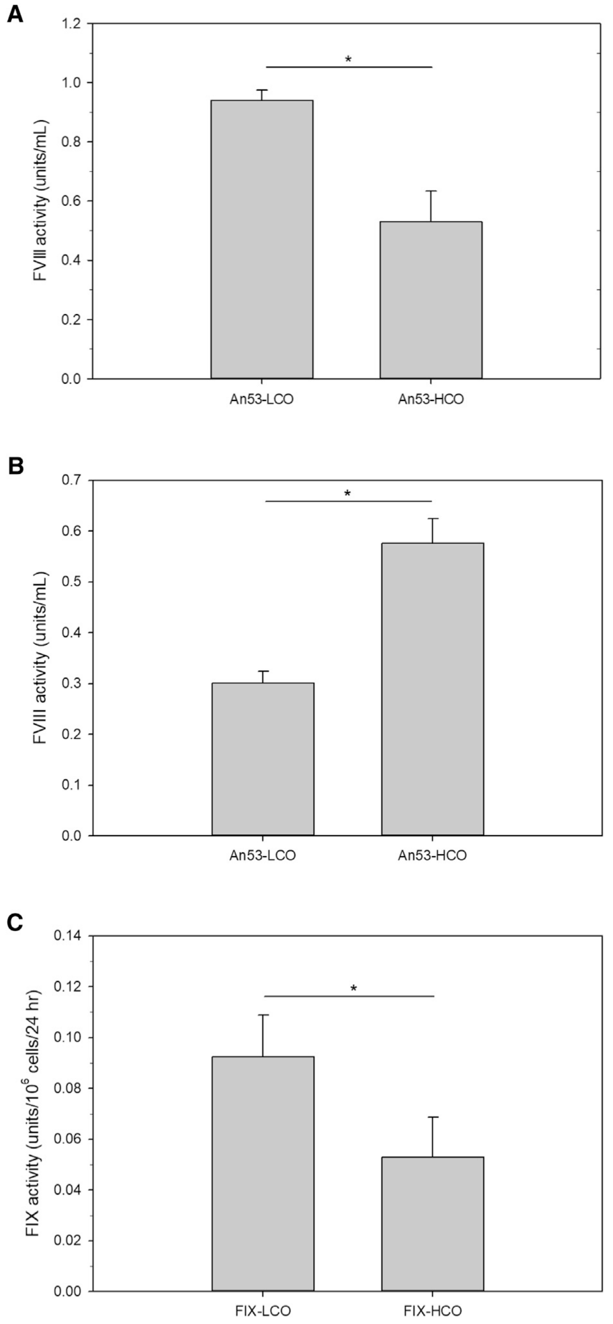


**Figure 3. Expression and Tissue Specificity of HCO versus LCO**

(A and B) An53-HCO and An53-LCO were transfected into (A) HepG2 cells and (B) BHK cells. fVIII activity from conditioned media was measured 48 hr after transfection (*$p < 0.05$ by two-tailed t test). (C) fIX-LCO and fIX-HCO plasmids were transfected into HepG2 cells supplemented with vitamin K. fIX activity was measured 48 hr after transfection (*$p < 0.05$ by two-tailed t test). Data are presented as the mean ± sample SD.

that the codons over-represented in the naturally liver-optimized genes are, as designed, over-represented in the synthetically optimized ET3-LCO and, likewise, that the codons under-represented natively also are under-represented in ET3-LCO.

Dittmar et al. demonstrated that liver CUB correlated with the cognate liver tRNA pool, whereby codons that were were under-represented in the 43 highly and specifically liver-expressed mRNAs had lower levels of each cognate tRNA isoacceptor within liver tissue, and, conversely, codons used preferentially predicted high levels of tRNA isoacceptor.[24] The central assumption of codon optimization is that these tRNA levels are the rate-limiting factor of protein translation wherein cDNAs that frequently utilize codons with low-level cognate tRNAs suffer from inefficient translation. To test this hypothesis, we constructed a plasmid to overexpress the 6 tRNAs that the ET3-LCO sequence predicts to be most limiting for ET3-NoCo (indicated with open circles in Figure 2C). Plasmids encoding ET3-LCO, ET3-MCO, and ET3-NoCo were cotransfected into HepG2 cells with either the 6x tRNA plasmid or a GFP control (Figure 2D). When cotransfected with GFP, ET3-LCO demonstrated 2-fold higher levels of fVIII production than ET3-MCO or ET3-NoCo. However when cotransfected with the 6x tRNA, ET3-LCO showed a slight but insignificant increase in fVIII production, while production levels of ET3-MCO and ET3-NoCo were elevated to that of non-tRNA supplemented ET3-LCO.

### *In Vitro* Testing of Tissue-Specific Codon Optimization

Using a cell-culture model of liver-directed expression, LCO was compared to HCO. Specifically, plasmids encoding An53-LCO and An53-HCO under control of the HLP promoter were transfected into HepG2 cells. As the sequence of An53 was predicted at the amino-acid level, the native (NoCo) sequence was not available. In this system, An53-LCO was expressed at levels nearly 2-fold greater than that of An53-HCO (Figure 3A). To test whether this effect was specific to liver-derived cells, the same plasmids were then transfected into baby hamster kidney (BHK) cells (Figure 3B). In this setting, An53-LCO was expressed at levels 2-fold lower than that of An53-HCO, supporting the conclusion that LCO provides benefit specifically in the context of liver-directed expression.

To ensure that the benefit of LCO was not specific to fVIII, we next applied LCO and HCO optimization strategies to human coagulation fIX cDNAs containing the gain-of-function Padua (R388L) mutation.[29] This naturally occurring mutation has been previously shown to increase the specific activity of fIX by approximately 8-fold and is being tested in clinical liver-directed AAV gene therapies.[30] LCO and HCO fIX cDNAs were inserted into an AAV plasmid cassette driven by the liver-directed transthyretin promoter and transfected into HepG2 cells (Figure 3C). In this system, fIX-LCO showed approximately 2-fold greater fIX activity than fIX-HCO, suggesting that the benefit of LCO beyond fVIII.

To further validate the tissue-directed nature of these findings, transfection studies were preformed using ET3-LCO, MCO, and NoCo, as

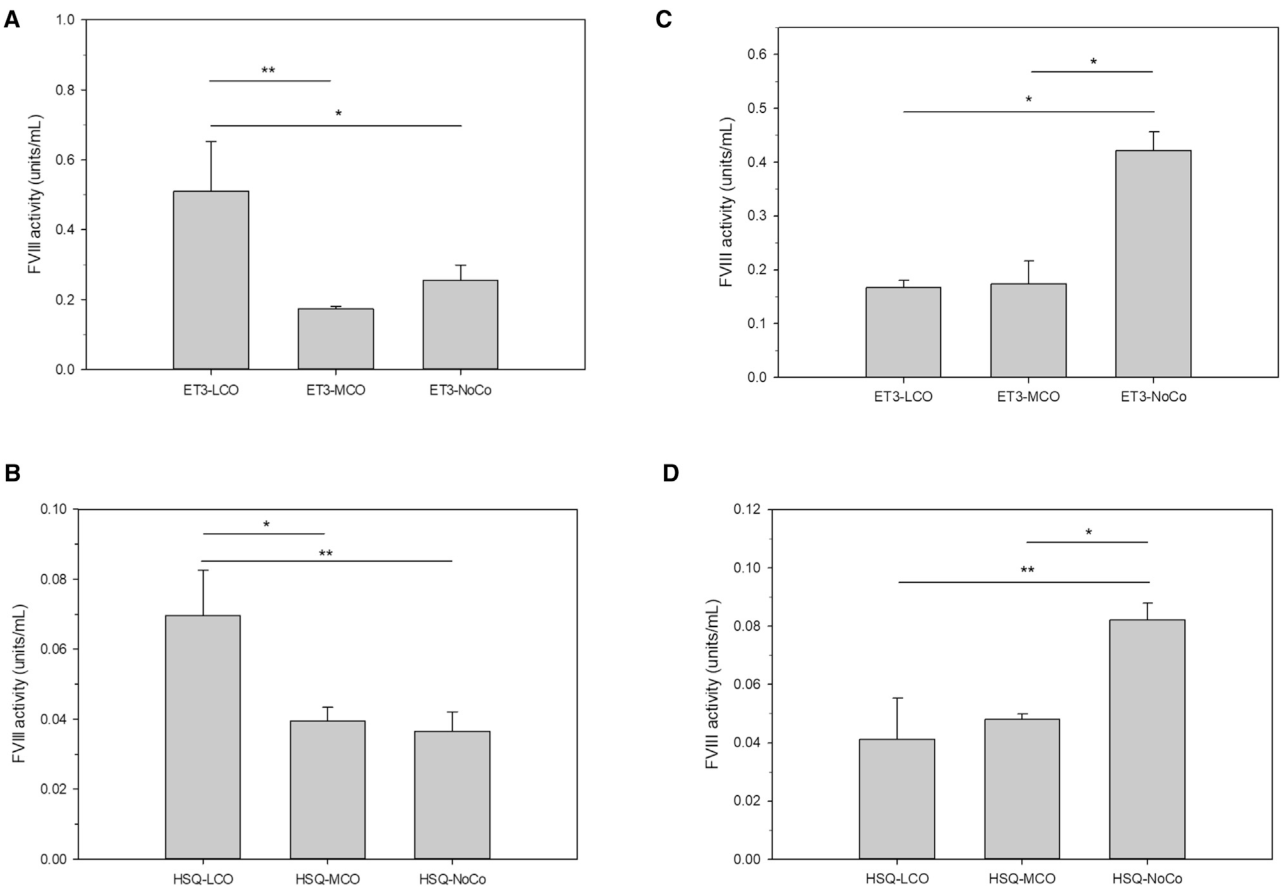


**Figure 4. Expression and Tissue Specificity of LCO, MCO, and NoCo Designs**

(A and B) HepG2 cells were transfected with (A) ET3-LCO-, ET3-MCO-, and ET3-MCO-encoding plasmids and (B) HSQ-LCO-, HSQ-MCO-, and HSQ-NoCo-encoding plasmids. fVIII activity in the conditioned media was measured 48 hr after transfection. In (A), *p = 0.011 and **p = 0.006, and in (B), *p = 0.006 and **p = 0.005 by one-way ANOVA and the Holm-Sidak method. (C and D) BHK cells were transfected with (C) ET3-LCO-, ET3-MCO-, and ET3-MCO-encoding plasmids and (D) HSQ-LCO-, HSQ-MCO-, or HSQ-NoCo-encoding plasmids. In (C), *p < 0.001, and in (D), *p = 0.007 and **p = 0.004 by one-way ANOVA and the Holm-Sidak method. Data are presented as the mean ± sample SD.

well as HSQ-LCO, MCO, and NoCo. When transfected into HepG2 cells, ET3-LCO and HSQ-LCO both showed significantly higher production than either the MCO or NoCo cognates (Figures 4A and 4B, respectively). In contrast, when transfected into the BHK cells, both MCO and LCO optimization decreased fVIII production relative to the non-optimized variants (Figures 4C and 4D). Together, these data show that LCO specifically provides benefit in the matched tissue and that this optimization is not an artifact of optimization against a small subset of highly expressed genes. Further, they support the conclusion that removal of deleterious *cis*-acting RNA motifs, a process that was applied equally to the MCO, LCO, and HCO designs, was not the dominant contributor to increased transgene productivity.

### Effect of Tissue-Specific Codon Optimization *In Vivo*

Expression plasmids encoding An53-HCO and An53-LCO were delivered by hydrodynamic injection to hemophilia A mice. In this system, An53-LCO provided a 7-fold increase in fVIII expression compared to An53-HCO (Figure 5A). Next, ET3- and HSQ-LCO, ET3-MCO, and ET3-NoCo were delivered utilizing the same system (Figure 5B). The benefit of LCO over MCO and NoCo again was observed with both ET3 and HSQ, with ET3-LCO providing a 3- to 4-fold increase in expression over both ET3-MCO and ET3-NoCo (Figure 5B). HSQ-LCO raised fVIII to detectable levels compared to HSQ-NoCo, representing a minimum of 6-fold improvement, trending toward a 4-fold increase in expression over MCO (Figure 5C). To determine whether the benefits were not due to altered pharmacokinetics, a time course experiment was performed using ET3 and HSQ-LCO and NoCo. Our previous experience has shown that delivery of linearized plasmid DNA provides prolonged gene expression compared to that of circular plasmid; however, maximum expression is not achieved for several days (data not shown). Plasmid DNA containing the transgene cassettes was delivered hydrodynamically to hemophilia A mice, and expression was monitored over 6 weeks (Figure 5D). Over the course of the experiment, the benefit of LCO over NoCo was replicated, with ET3-LCO providing a sustained 7-fold increase in fVIII

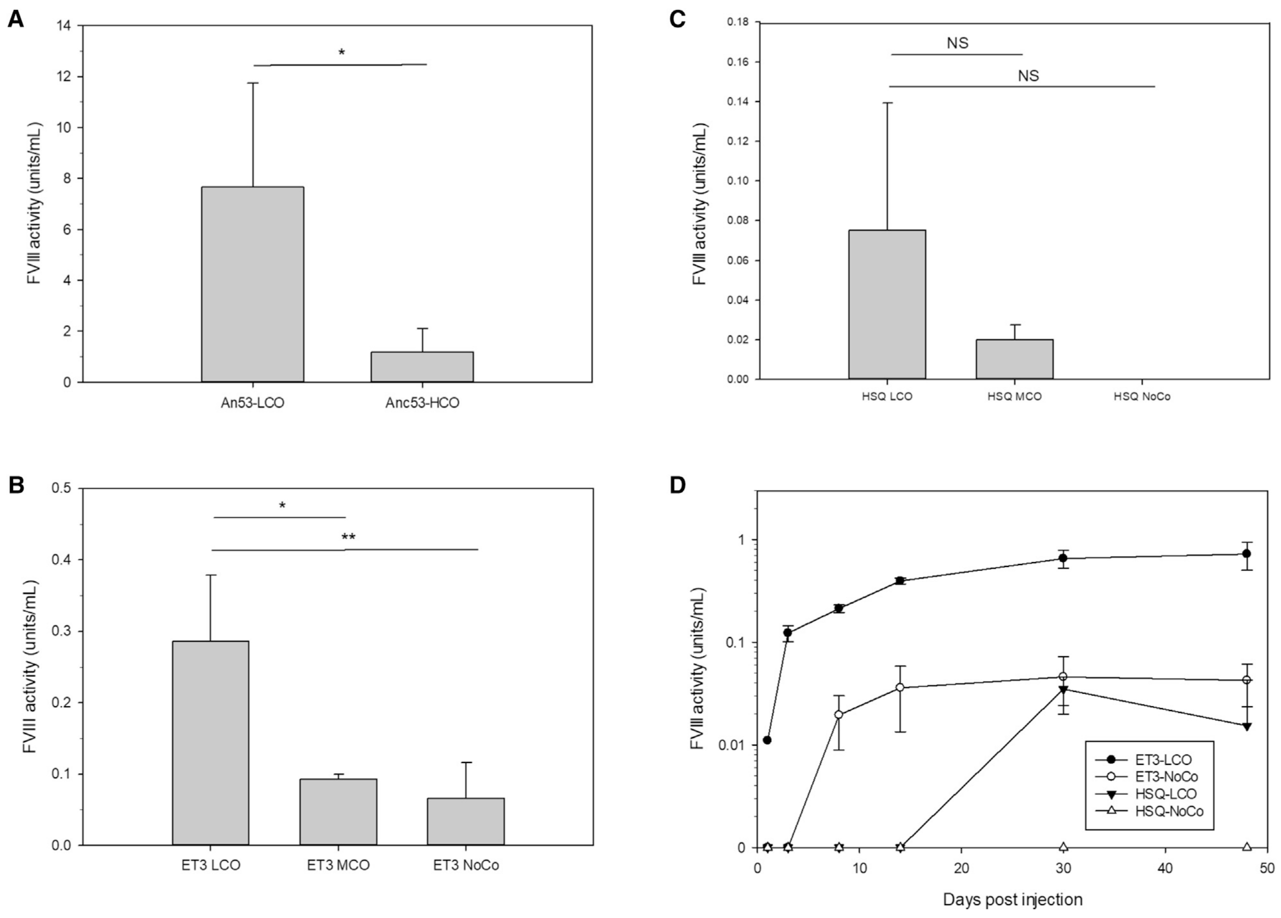


**Figure 5. In Vivo Expression of Codon-Optimized Designs**

(A–C) Shown here: (A) An53-HCO and An53-LCO; (B) ET3-LCO, ET3-MCO, and ET3-NoCo; and (C) HSQ-LCO, HSQ-MCO, and HSQ-NoCo plasmids were hydrodynamically injected into hemophilia A mice (n = 3–4 for all groups). Plasma levels of fVIII activity were measured 24 hr after transfection. In (A), *$p < 0.05$ by two-tailed t test, and in (B), *$p = 0.05$ and **$p = 0.004$ by one-way ANOVA and the Holm-Sidak method. NS, not significant. (D) Linearized ET3 and HSQ-LCO and NoCo were hydrodynamically injected into hemophilia A mice. Plasma levels of fVIII activity were measured for 6 weeks post-injection. Data are presented as the mean ± sample SD.

expression. HSQ-LCO also improved fVIII production to detectable levels, which was not achieved with HSQ-NoCo.

### AAV-Mediated Delivery of Optimized Constructs

AAV2 ITR expression cassettes containing fVIII cDNAs and the novel synthetic promoters were constructed and packaged into AAV8 capsids (Figure 6A). Initially, the benefit of LCO versus HCO was determined in the context of *in vivo* liver-directed gene therapy. To evaluate this, AAV2/8 An53-LCO and An53-HCO cDNAs driven by the HLP promoter were synthesized. Single doses of $1 \times 10^{11}$ vg/kg of AAV2/8-HLP-An53-LCO and AAV2/8-HLP-An53-HCO were delivered intravenously to hemophilia A mice. In this system, the benefit of LCO over HCO was again recapitulated, with An53-LCO demonstrating persistent 4-5-fold greater fVIII activity over An53-HCO (Figure 6B).

Our previously described vector, the non-optimized AAV-HCR-ET3-NoCo, suffered from substantial decline in fVIII expression over the duration of a 48-week experiment.[6] To test the durability of our optimized vector, mice were dosed at $1 \times 10^{11}$ vg/kg or $6.2 \times 10^{9}$ vg/kg, with modified AAV-HCB-ET3-LCO containing an minute virus of mince (MVM) intron (Figures 6A and 6C). In contrast to the previous HCR-based design, this new vector demonstrated sustained expression throughout the duration of the experiment; however, the inclusion of the MVM intron, which was unique to this experiment, cannot be disregarded as a contributor to the stable expression conferred by this design. Further interrogation did not observe any benefit or negative impact from the MVM intron on the fVIII transgene constructs studied (data not shown). Therefore, to maintain compatibility with the limited packaging capacity of AAV, we did not include the MVM intron in subsequent studies.

The minimally sized constructs lacking the MVM intron were packaged into AAV8 capsids. These transgene constructs consisted of LCO fVIII designs driven by the HCB promoter, terminated by a minimal synthetic beta-globin polyadenylation signal, and flanked by

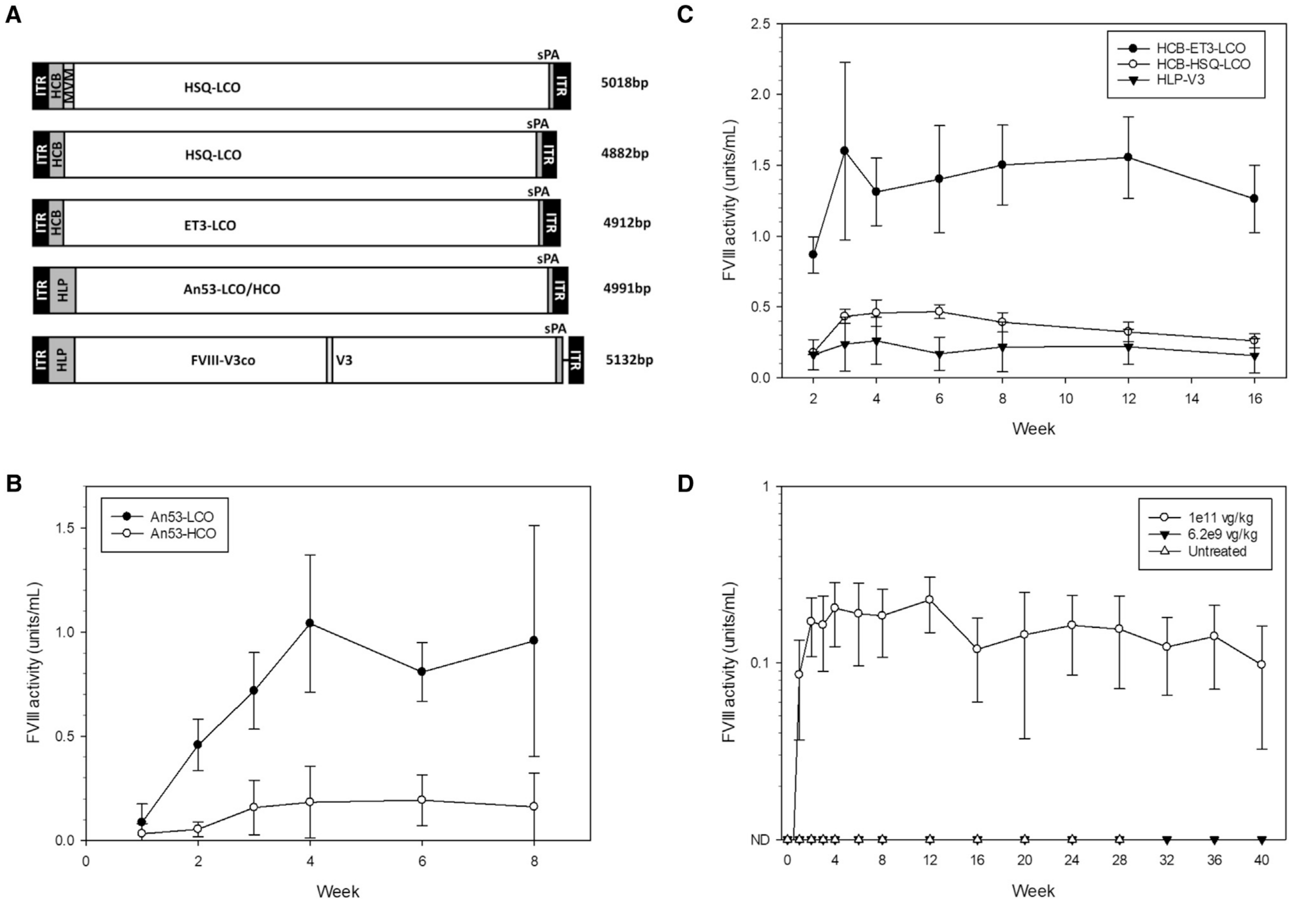


**Figure 6. AAV Vector Delivery of Tissue Optimized Transgene Cassettes**

(A) Schematics of AAV2/8 transgene designs. Straight lines represent DNA sequence of no known function. (B) AAV2/8-HLP-An53-LCO and AAV2/8-HLP-An53-HCO were delivered intravenously to hemophilia A mice at a dose of 1 × $10^{11}$ vg/kg (n = 4 per group). (C) AAV2/8 HCB-HSQ-LCO, AAV2/8-HCB-ET3-LCO, and AAV2/8-HLP-V3co were delivered intravenously to hemophilia A mice at a dose of 1 × $10^{11}$ vg/kg (n = 3–4 per group). (D) A long-term, dose-finding experiment was performed with varying does of AAV2/8-HCB-MVM-ET3-LCO (n = 3 for all other doses). Data are presented as the mean ± sample SD.

AAV2 ITRs (Figure 6A). At sizes of 4,882 bp (HSQ) and 4,912 bp (ET3), to our knowledge, these vectors are the shortest AAV-fVIII vectors described to date. As a comparator, AAV2/8-HLP-fVIII-V3co also was packaged into AAV8 capsids. fVIII-V3co is a previously described high-expression, codon-optimized human fVIII variant that contains a novel 17-amino-acid (aa) peptide (V3) in which 6 glycosylation sites from the fVIII B domain are juxtaposed and then fused to the linker sequence joining the fVIII A3 and C1 domains. V3 was shown previously to increase fVIII expression 2-fold, and AAV-HLP-fVIII-V3co represents one of the most potent AAV-fVIII vectors described to date.[12] When delivered to hemophilia A mice at a vector dose of 1 × $10^{11}$ vg/kg, AAV-HCB-HSQ-LCO achieved persistent fVIII activity of approximately 0.4 IU/mL, which was greater than that achieved with AAV-HLP-V3co (Figure 6D). From the optimization described herein, these vectors provide curative levels of fVIII expression at doses that are 20-fold lower than the maximum clinical dose of AAV8 utilized at 2 × $10^{12}$ vp/kg (Figure 6D).

## DISCUSSION

Efforts to develop clinical AAV-fVIII vector candidates for hemophilia A have been hampered by the large size of the BDD fVIII cDNA, the poor biosynthetic efficiency of human fVIII, and vector dose-limiting toxicity. Ideally, a clinical AAV-fVIII vector would be of sufficient potency to confer clinically beneficial (>1% of normal) or preferably curative (>40% of normal) circulating fVIII activity at a vector dose compatible with manufacturing, safety, and efficacy. Recently, a phase 1/2 clinical trial of an AAV5-HLP-BDD-cohfVIII gene therapy vector for hemophilia A was conducted, and interim results have been presented at the 2017 International Society of Thrombosis and Haemostasis Congress and the American Society of Hematology Annual Meeting. This vector utilizes the HLP promoter driving a presumably human CUB-optimized BDD human fVIII packaged in AAV5. Under this clinical protocol, patients were administered low (6 × $10^{12}$ vg/kg), middle (2 × $10^{13}$ vg/kg), middle-high (4 × $10^{13}$ vg/kg), and high (6 × $10^{13}$ vg/kg) doses of vector. For the low and middle vector doses, single subjects in each cohort

demonstrated peak plasma fVIII activity levels at <1% and 2%, respectively. At the medium-high and high doses, patients showed dose-dependent benefit, ranging from partial to complete phenotypic correction. However, at these doses, most patients predictably suffered from transaminitis that was controlled through a course of corticosteroids. While these clinical trial data clearly represent a breakthrough in gene therapy for hemophilia A that previously has failed to deliver any signs of efficacy, there remains a cogent need to continue the development of AAV-fVIII vectors with improved potency and safety.

Current good manufacturing practice (cGMP) production of recombinant AAV typically results in yields less than $10^{15}$ vg per full-scale manufacturing run, which typically is on the order of 100 L of raw harvest material.[31] At the dose recommended for phase 3 testing in the aforementioned AAV5-HLP-BDD-hfVIII trial (highest dose), this would equate to $6 \times 10^{13}$ vg/kg $\times$ 70 kg (average human male body weight) or $4.2 \times 10^{15}$ vg per patient. Therefore, even if manufacturing yields are improved many fold or the scale is increased several-fold, only a handful of patients could be treated with a single batch of vector product. Therefore, this target product profile appears suboptimal for global utilization. The goal of the present study was to develop a vector with sufficient pharmacodynamic performance and potency to confer curative levels of fVIII expression at clinically safe vector doses in a liver-directed manner. By reducing the dose requirement, the immunogenicity risk should also be lowered and the manufacturing bottleneck relieved.[32]

Recently, we described the pre-clinical testing of a 5.86-kb AAV2/8 vector encoding the bioengineered ET3 transgene.[6] While this vector was of sufficient potency to partially or completely correct the hemophilia A phenotype at vector doses of $5 \times 10^{11}$ to $2 \times 10^{13}$ vg/kg, due to the large size of the transgene, this vector suffers from substantial intraparticle heterogeneity and low-yield vector manufacture. To reduce the size of this vector, and thus improve the potency and manufacture, we initiated studies to generate a completely synthetic AAV cassette, wherein unnecessary viral DNA remnants that were present in early-generation AAV vector designs were removed, leaving only the minimal AAV elements and enzyme restriction sites to allow simple substitution of discrete elements. This procedure alone removed 210 bp from our first-generation vector design. We further substituted a 49-bp synthetic poly(A) polyadenylation signal for our original 260-bp bovine growth hormone poly(A), removing an additional 211 bp. While these modifications reduced the size of the vector transgene to 5.45 kb, it remained substantially oversized (Figure S1).

To further reduce the size of the vector, we utilized an iterative "design, build, test" process to generate a minimally sized yet highly active promoter. In a process similar to, but distinguishable from, those used to generate the previously described "super core promoter" and "HSCRM8" transcriptional enhancer, we utilized combinatorial assembly of previously described elements to iteratively and empirically generate a promoter with the necessary size and strength required for a clinically oriented AAV-fVIII vector.[33,34] Beginning with the SynO and AFP core elements, the impact of additional transcriptionally oriented modules proved to not be discretely additive but rather highly dependent on the interaction between specific modules present in a single design. We attribute this to the exquisite complexity of the interaction and contextually dependent function of TFs, a process that remains difficult to define, and methods for detecting these interactions in real time are only recently being realized.[35,36]

Through this iterative process, we arrived at an enhancer consisting of the HNF1 TF binding site proximal to the shortened Abp sequence. This enhancer is fused to the SynO core promoter, which is modified with a defined TSS, a combination that we designated as the HCB. At a total size of 146 bp, this minimal promoter was shown to drive ET3 transcription at levels 2-fold higher than that of the 252-bp HLP *in vitro* and 14-fold higher than that of the HLP *in vivo*. At this size, when substituted into our synthetic AAV transgene cassette, the HCB promoter, in conjunction with HSQ, results in a vector transgene of only 4.88 kb in length, which is expected to be compatible with complete genome packaging and improved homogeneity and yield of vector product.

The second significant barrier to AAV gene therapy for hemophilia A is the poor biosynthetic efficiency of the mature fVIII protein itself, which is secreted at levels 2–3 orders of magnitude lower than similarly sized glycoproteins.[9] This poor secretory efficiency has been spatially and temporally linked between inefficient trafficking from the endoplasmic reticulum (ER) to the Golgi due to induction of the unfolded protein response.[13,15,37] Our group and others have made efforts to improve the efficiency of secretion by bioengineering the protein itself. These approaches have included substitution of the fVIII A1/A3 domains with porcine fVIII sequences (ET3), inclusion of additional glycosylation sites within the truncated B-domain linker (V3), and reconstruction of ancestral fVIII protein sequences (An53). In the case of ET3 and An53, bioengineering the fVIII molecule results in 10-fold to 100-fold more efficient protein expression.

Codon optimization has been routinely utilized in AAV vector designs, both in pre-clinical and now clinical studies. While this approach has been shown to confer benefit in some expression systems, recent studies quantifying the physical pools of tRNA within different tissues have shown that the assumptions made by codon optimization may not be reflective of an optimal strategy in certain tissues, including the liver. A commonly accepted causative factor for drift of CUB across evolution is the tolerance of the protein product of particular cDNA for missense mutations,[38–41] a mechanism that would allow for coadaptation of synonymous codon mutations and the tRNA milieu within a particular tissue to allow for optimal gene expression. Investigation of tissue-specific bias of the human liver and myeloid lineage cells revealed that the drift in CUB of genes highly expressed in these tissues compared to the entire human CDS is, in fact, greater than the drift between the human and murine CDS CUBs. Therefore, we reasoned that codon optimizing to the target tissue of expression may offer benefit greater than that offered by

optimization to the entire genome. Building on the work of Dittmar et al., we synthesized variants of HSQ, ET3, and An53 codon optimized for expression in the liver based on the CUB of genes highly expressed within the liver, as well as controls optimized using the standard human codon index and a myeloid index. A similar but distinguishable approach has been previously reported by Nathwani and colleagues[3] in the context of liver-directed fIX expression; however, the details remain poorly described in the scientific literature. Herein, we completely describe the approaches used and provide the strategy and tools required to use a similar approach to other relevant transgenes and optimized vectors. Previously, we generated a human codon-optimized version of ET3 (ET3-HCO). ET3-HCO demonstrated no significant difference in ET3 expression relative to ET3-NoCo; therefore, within the context of the experiments described herein, ET3 and HSQ NoCo were utilized as controls for tissue-specific codon optimization.

*In vitro* studies show that liver codon optimization specifically increases fVIII expression of all fVIII variants within HepG2 cells while diminishing fVIII expression in the non-hepatic BHK cell line, relative to either human codon optimized or myeloid optimized variants. Further, these findings were extended to fIX expression as well, demonstrating that the benefit of tissue-specific codon optimization is not limited to fVIII. Importantly, these optimized constructs all were generated using the same algorithm but utilizing different codon usage indexes. While adjustment of the codon usage index and optimal codon distribution are central aspects of codon optimization, modern codon optimization algorithms also attempt to remove deleterious non-coding RNA motifs, such as destabilizing and structural features. By utilizing the same algorithm, only adjusted by the codon usage index, these reciprocal experiments strongly suggest that the benefit of liver codon optimization is, in fact, due to the unique tRNA pool within the liver and not simply the alteration of RNA motifs. However, future studies interrogating steady-state mRNA levels will be required to fully elucidate the mechanism underlying the enhanced *in vitro* expression conferred by LCO.

The effect of tissue-directed codon optimization was shown to be dependent on only a small number of individual tRNA species. Conversely, one can conceive that, by identifying the tRNAs most limiting to native sequence expression, synonymous codon substitution may be used to dampen off-target transgene expression without affecting expression in the desired tissue. Furthermore, transgenes could be designed in bi- or polycistronic constructs to include the therapeutic gene of interest as well as the limiting tRNA(s) for enhanced production.

When these codon-optimized variants were delivered to the livers of hemophilia A mice by hydrodynamic injection, the benefit of liver codon optimization became more pronounced. When compared to the human codon-optimized variant, An53-LCO showed a 7-fold increase in fVIII expression. This benefit of liver optimization was, again, recapitulated by AAV-mediated delivery of An53, where LCO exhibited 4-5-fold benefit over HCO for the duration of the experiment. Likewise, when compared to the myeloid optimized variants, HSQ and ET3 both showed 4-fold improvement. Therefore, the HepG2 cell-culture model appears to have underestimated the benefit of liver codon optimization. However, future studies will be required to further define the underlying mechanism behind the enhanced fVIII expression conferred by LCO in the context of murine liver.

While we and others have shown codon optimization to increase expression of functional fVIII and fIX, such optimization may not come without risk. The possible deleterious effects of codon optimization, which may presumably be heightened by tissue-specific codon optimization, has been reviewed by Mauro and Chappell.[42] A specific concern raised by the authors is that evolution may have selected for rare codons to slow translation at critical regions to allow time for proper protein folding. Future investigation will need to be performed to interrogate the folding of codon-optimized proteins as well as potential engagement of unfolded protein response (UPR) pathways when expressing codon-optimized proteins.

tRNA dysregulation is emerging as an important factor driving the increased protein translation and proliferation in both primary cancer tissues and cancer-derived cell lines.[43,44] In cancer-derived cell lines, global tRNA levels have been shown to be elevated 3- to 5-fold over those in non-cancer-derived cell lines, while in primary breast cancer tissue, tRNA levels were found to be elevated 10-fold over those in normal breast tissue.[43] This expected difference in tRNA levels between the human liver carcinoma-derived HepG2 cell line and those in the normal hepatocytes of hemophilia A mice may explain the attenuated benefit of LCO *in vitro* compared to the benefits observed *in vivo*, although this postulation will require further investigations.

After completion of early optimization studies, our lead candidate vectors, AAV-HCB-HSQ-LCO and AAV-HCB-ET3-LCO, were packaged into AAV8 capsids and delivered to hemophilia A mice at a dose of 1e11 vg/kg. At this low vector dose, the HSQ-vector-infused mice expressed fVIII at 0.3–0.4 IU/mL. When linearly scaled, the potency of this virus is comparable to that shown by the previously described AAV-HLP-V3co design. However, much of the benefit of the AAV-HLP-V3co design was attributed to the substitution of the V3 linker, a novel 17-aa linker that contains 6 glycosylation sites. Lacking this V3 linker, fVIII expression from the fully human cDNA in the same cassette declined 3-fold. We have now achieved comparable levels of fVIII expression from the fully human AAV-HCB-HSQ-LCO design, representing a significant step forward toward a clinically viable, highly potent, AAV-fVIII vector.

## MATERIALS AND METHODS

### Codon Usage Indexes and Codon Optimization

The liver codon optimization table was previously described, utilizing sequences of highly and specifically expressed RNAs within hepatocytes.[24] The myeloid optimization table was generated by examining previously published Affymetrix mRNA array data using the GEO: GSE16836 dataset.[45] The myeloid CUB was determined from

cDNA sequences from highly and specifically expressed RNAs utilizing the publicly available Sequence Manipulation Suite.[46] Individual CUBs of all human genes were determined using gene sequences retrieved from the Consensus CDS Sequence Project annotation of the GRCh38 reference genome.[47] HSQ and ET3-LCO and -MCO cDNA sequences were depleted of all instances of cytosine located 5′ to a guanosine (CpG) by substitution of the synonymous codon with the highest CUB, which did not result in the introduction of another CpG motif. HSQ- and ET3-NoCo were left as native human and human/porcine sequences, respectively, containing 175 and 174 CpG instances, respectively. The CpG content of An53-LCO and An53-HCO was not adjusted, containing 115 and 156 instances of CpG, respectively. All cDNA sequences described herein are given in the Supplemental Information.

#### DNA Synthesis

The synthetic AAV2 expression cassette was constructed by Genscript Biotech and inserted into the pUC57 backbone. Promoter DNA sequences were generated as individual gBlocks by Integrated DNA Technologies. gBlocks were cloned into the AAV vector using *AgeI*/SalI sites included in the gBlock design using the SalI-compatible *XhoI* site within the AAV vector. Complete fVIII DNA sequences were synthesized by Genscript. Codon optimization was performed using the Genscript Optimum Gene algorithm using the standard human CUB index for human codon optimization table or custom liver and myeloid CUB indexes. fVIII sequences were cloned into the AAV backbone using XhoI/NotI restriction sites. The sequence of An53 was determined by ancestral sequence reconstruction as previously described and optimized using human and liver CUB tables using the OptimumGene algorithm.[19] The AAV-HLP-V3 transgene sequence was generated from a previously published sequence.[12]

#### tRNA Expression Plasmid

The 6x tRNA expression sequence was synthesized by Genscript and inserted into the pUC57 backbone. tRNA sequences and chromosomal locations were retrieved from the genomic tRNA database.[48] Genomic sequences of 200 bp, both upstream and downstream of the tRNA sequence itself, were included to preserve the local genomic context as well as to capture local transcriptional regulators and proximal sequence elements. The sequence of the tRNA expression plasmid is given in the Supplemental Information.

#### Transfection Studies

Low-passage HepG2 cells were plated at a density of 200,000 cells per well in 500 μL DMEM/F12 supplemented with 10% fetal bovine serum and 1% penicillin/streptomycin in 6-well plates. Twenty-four hours after seeding, cells were transfected using 1.5 μL Mirus Bio TransIT-X2 Transfection Reagent and 500 ng plasmid according to the manufacturer's instructions. For tRNA cotransfection, 250 ng fVIII expression plasmid was co-transfected with either 250 ng of 6x tRNA or GFP control plasmid. To control for transfection efficiency, all transfections were performed from a common master mix of transfection reagent and applied to cells that were plated simultaneously from a common pool of cells. For all transfection experiments, all plasmid components were identical, including plasmid backbone, ITRs, coding regions, and regulatory control regions, apart from the component undergoing investigation. All plasmid DNA was quantified by spectrophotometric method on the day of transfection. Media were changed the following day, and supernatants were assayed for fVIII activity after 24 hr. For fIX transfections, media were supplemented with 5 μg/mL vitamin K 24 hr post-transfection.

#### fVIII and fIX Activity Measurements

fVIII activity in conditioned supernatant was performed by activated partial thromboplastin (aPTT) reagent-based one-stage coagulation assay, as previously described.[28] Briefly, 5 μL conditioned supernatant was diluted in 50 μL fVIII-deficient plasma. 50 μL aPTT was then added, and samples were incubated at 37°C for 4 min. Following incubation, 50 μL of 20 mM calcium was added. Time to clot formation was determined by automated viscometric measurement. fVIII activity was determined by linear regression analysis of the clotting time versus the logarithm of the reciprocal plasma dilution. fVIII activity in plasma samples was measured using the Diapharma Coatest $SP_4$ fVIII Kit, according to the manufacturer's instructions. fVIII activity was determined by comparison to a pooled normal plasma (FACT, George King Biomedical) standard curve. fIX activity measurement and calculation were conducted identically, with the exception of the incubation time after addition of aPTT, which was reduced to 3 min, and fVIII-deficient plasma was substituted for fIX-deficient plasma.

#### Hydrodynamic Injections

250 ng (ET3 and An53) or 500 ng (HSQ) expression plasmids were diluted into Mirus Bio TransIT-EE hydrodynamic injection solution in a volume equivalent to $1/10^{th}$ the recipient mouse's weight. Prior to delivery, HSQ and ET3 plasmid DNA were linearized using *XbaI* and *HpaI* restriction sites located immediately outside of the ITR sequences. An53-HCO plasmid was linearized using *SapI* and *SacI*, and An53-LCO plasmid was linearized using *MluI* and *NsiI*, also located immediately outside of the ITR sequences. Prior to injection, an aliquot of digested plasmid was run on a 0.8% agarose gel to ensure complete digestion and to ensure that comparable DNA quantity and quality were achieved (data not shown). Plasmid was delivered as a single bolus into the tail vein over 5–10 s using 3-mL syringes and 26 5/8G needles. Mice were allowed to recover on heating pads for 30 min. Blood was drawn by retro-orbital method into citrated tubes beginning at 24 hr after injections.

#### AAV Production and Titration

AAV8 production was performed by ViGene Biosciences. Vector titers were determined in house by the PCR method. Vector particles were DNase treated for 30 min, and then DNase was inactivated by adding EGTA and heat inactivating at 65°C for 10 min. Packaged DNA was liberated by the addition of Proteinase K and heated for 1 hr at 65°C. Proteinase K was then inactivated by heating at 95°C for 20 min. DNA concentration was determined by qPCR using a standard curve generated from the appropriate AAV expression plasmid using primers directed toward the center of the viral transgene.

### RNA Quantification

RNA from AAV-treated mice was extracted from saline-perfused livers of sacrificed mice using the QIAGEN RNeasy RNA Isolation Kit. qRT-PCR was performed as previously described.[37] RNA was quantified by comparison to individual standard curves generated from the appropriate AAV-fVIII expression plasmid.

### Animal Studies

All animal studies were performed under the guidelines set by the Emory University Institutional Animal Care and Use Committee. Exon-16-disrupted mice backcrossed onto a C57BL/6 background were used as a model of hemophilia A.[49] Hydrodynamic injections were performed in male and female 8- to 12-week-old mice. AAV studies were performed in 8- to 12-week-old male mice. Plasma sampling was performed under anesthesia by retro-orbital method into citrated capillary tubes.

### Statistical Methods

Experimental datasets were analyzed either by two-tailed Student's t test for single comparisons or by one-way ANOVA for data involving multiple comparisons. For the latter, post hoc analysis was performed using the Holm-Sidak method to determine individual p values. All statistical analysis was performed using SigmaPlot software.

## SUPPLEMENTAL INFORMATION

Supplemental Information includes Supplemental Materials and methods and two figures and can be found with this article online at https://doi.org/10.1016/j.omtm.2018.01.004.

## AUTHOR CONTRIBUTIONS

H.C.B., H.T.S., and C.B.D. conceived the project and designed the experiments. H.C.B., P.M.Z., S.N.G., and E.T.P. performed experiments and analyzed the data. H.C.B. and C.B.D. drafted the manuscript. H.C.B., P.M.Z., H.T.S., and C.B.D. edited the manuscript.

## CONFLICTS OF INTEREST

H.C.B., H.T.S., and C.B.D. are inventors on a patent application describing the liver-directed fVIII codon-optimization and promoter technology filed by Emory University and Children's Healthcare of Atlanta. C.B.D., H.T.S., and P.M.Z. are inventors on a patent application describing ancestral fVIII technology filed by Emory University/Children's Healthcare of Atlanta and Georgia Institute of Technology. C.B.D. and H.T.S. are co-founders of Expression Therapeutics and own equity in the company. Expression Therapeutics owns the intellectual property associated with ET3 and has obtained licenses for the ancestral fVIII, liver-codon-optimized fVIII, and synthetic liver-directed promoter intellectual property. The terms of this arrangement have been reviewed and approved by Emory University in accordance with its conflict of interest policies.

## ACKNOWLEDGMENTS

This work was supported by funding from the NIH/National Heart, Lung and Blood Institute (HL112309, HL131059, and HL137128 to H.T.S. and C.B.D.).

## REFERENCES

1. Kay, M.A., Manno, C.S., Ragni, M.V., Larson, P.J., Couto, L.B., McClelland, A., Glader, B., Chew, A.J., Tai, S.J., Herzog, R.W., et al. (2000). Evidence for gene transfer and expression of factor IX in haemophilia B patients treated with an AAV vector. Nat. Genet. *24*, 257–261.
2. Manno, C.S., Pierce, G.F., Arruda, V.R., Glader, B., Ragni, M., Rasko, J.J., Ozelo, M.C., Hoots, K., Blatt, P., Konkle, B., et al. (2006). Successful transduction of liver in hemophilia by AAV-Factor IX and limitations imposed by the host immune response. Nat. Med. *12*, 342–347.
3. Nathwani, A.C., Gray, J.T., Ng, C.Y., Zhou, J., Spence, Y., Waddington, S.N., Tuddenham, E.G., Kemball-Cook, G., McIntosh, J., Boon-Spijker, M., et al. (2006). Self-complementary adeno-associated virus vectors containing a novel liver-specific human factor IX expression cassette enable highly efficient transduction of murine and nonhuman primate liver. Blood *107*, 2653–2661.
4. Nathwani, A.C., Reiss, U.M., Tuddenham, E.G., Rosales, C., Chowdary, P., McIntosh, J., Della Peruta, M., Lheriteau, E., Patel, N., Raj, D., et al. (2014). Long-term safety and efficacy of factor IX gene therapy in hemophilia B. N. Engl. J. Med. *371*, 1994–2004.
5. Nathwani, A.C., Tuddenham, E.G., Rangarajan, S., Rosales, C., McIntosh, J., Linch, D.C., Chowdary, P., Riddell, A., Pie, A.J., Harrington, C., et al. (2011). Adenovirus-associated virus vector-mediated gene transfer in hemophilia B. N. Engl. J. Med. *365*, 2357–2365.
6. Brown, H.C., Wright, J.F., Zhou, S., Lytle, A.M., Shields, J.E., Spencer, H.T., and Doering, C.B. (2014). Bioengineered coagulation factor VIII enables long-term correction of murine hemophilia A following liver-directed adeno-associated viral vector delivery. Mol. Ther. Methods Clin. Dev. *1*, 14036.
7. Hirsch, M.L., Wolf, S.J., and Samulski, R.J. (2016). Delivering transgenic DNA exceeding the carrying capacity of AAV vectors. Methods Mol. Biol. *1382*, 21–39.
8. Wu, Z., Yang, H., and Colosi, P. (2010). Effect of genome size on AAV vector packaging. Mol. Ther. *18*, 80–86.
9. Kaufman, R.J., Pipe, S.W., Tagliavacca, L., Swaroop, M., and Moussalli, M. (1997). Biosynthesis, assembly and secretion of coagulation factor VIII. Blood Coagul. Fibrinolysis *8* (*Suppl 2*), S3–S14.
10. Nathwani, A.C., Rosales, C., McIntosh, J., Rastegarlari, G., Nathwani, D., Raj, D., Nawathe, S., Waddington, S.N., Bronson, R., Jackson, S., et al. (2011). Long-term safety and efficacy following systemic administration of a self-complementary AAV vector encoding human FIX pseudotyped with serotype 5 and 8 capsid proteins. Mol. Ther. *19*, 876–885.
11. Lu, H., Chen, L., Wang, J., Huack, B., Sarkar, R., Zhou, S., Xu, R., Ding, Q., Wang, X., Wang, H., and Xiao, W. (2008). Complete correction of hemophilia A with adeno-associated viral vectors containing a full-size expression cassette. Hum. Gene Ther. *19*, 648–654.
12. McIntosh, J., Lenting, P.J., Rosales, C., Lee, D., Rabbanian, S., Raj, D., Patel, N., Tuddenham, E.G., Christophe, O.D., McVey, J.H., et al. (2013). Therapeutic levels of FVIII following a single peripheral vein administration of rAAV vector encoding a novel human factor VIII variant. Blood *121*, 3335–3344.
13. Brown, H.C., Gangadharan, B., and Doering, C.B. (2011). Enhanced biosynthesis of coagulation factor VIII through diminished engagement of the unfolded protein response. J. Biol. Chem. *286*, 24451–24457.
14. Doering, C.B., Denning, G., Dooriss, K., Gangadharan, B., Johnston, J.M., Kerstann, K.W., McCarty, D.A., and Spencer, H.T. (2009). Directed engineering of a high-expression chimeric transgene as a strategy for gene therapy of hemophilia A. Mol. Ther. *17*, 1145–1154.
15. Doering, C.B., Healey, J.F., Parker, E.T., Barrow, R.T., and Lollar, P. (2004). Identification of porcine coagulation factor VIII domains responsible for high level expression via enhanced secretion. J. Biol. Chem. *279*, 6546–6552.
16. Johnston, J.M., Denning, G., Doering, C.B., and Spencer, H.T. (2013). Generation of an optimized lentiviral vector encoding a high-expression factor VIII transgene for gene therapy of hemophilia A. Gene Ther. *20*, 607–615.
17. Lytle, A.M., Brown, H.C., Paik, N.Y., Knight, K.A., Wright, J.F., Spencer, H.T., and Doering, C.B. (2016). Effects of FVIII immunity on hepatocyte and hematopoietic stem cell-directed gene therapy of murine hemophilia A. Mol. Ther. Methods Clin. Dev. *3*, 15056.

18. Spencer, H.T., Denning, G., Gautney, R.E., Dropulic, B., Roy, A.J., Baranyi, L., Gangadharan, B., Parker, E.T., Lollar, P., and Doering, C.B. (2011). Lentiviral vector platform for production of bioengineered recombinant coagulation factor VIII. Mol. Ther. *19*, 302–309.

19. Zakas, P.M., Brown, H.C., Knight, K., Meeks, S.L., Spencer, H.T., Gaucher, E.A., and Doering, C.B. (2017). Enhancing the pharmaceutical properties of protein drugs by ancestral sequence reconstruction. Nat. Biotechnol. *35*, 35–37.

20. Dong, H., Nilsson, L., and Kurland, C.G. (1996). Co-variation of tRNA abundance and codon usage in *Escherichia coli* at different growth rates. J. Mol. Biol. *260*, 649–663.

21. Garel, J. (1976). Quantitative adaptation of isoacceptor tRNAs to mRNA codons of alanine, glycine and serine. Nature *260*, 805–806.

22. Garel, J.P., Mandel, P., Chavancy, G., and Daillie, J. (1970). Functional adaptation of tRNAs to fibroin biosynthesis in the silkgland of Bombyx mori L. FEBS Lett. *7*, 327–329.

23. Gouy, M., and Gautier, C. (1982). Codon usage in bacteria: correlation with gene expressivity. Nucleic Acids Res. *10*, 7055–7074.

24. Dittmar, K.A., Goodenbour, J.M., and Pan, T. (2006). Tissue-specific differences in human transfer RNA expression. PLoS Genet. *2*, e221.

25. Ryffel, G.U., Kugler, W., Wagner, U., and Kaling, M. (1989). Liver cell specific gene transcription in vitro: the promoter elements HP1 and TATA box are necessary and sufficient to generate a liver-specific promoter. Nucleic Acids Res. *17*, 939–953.

26. Godbout, R., Ingram, R., and Tilghman, S.M. (1986). Multiple regulatory elements in the intergenic region between the alpha-fetoprotein and albumin genes. Mol. Cell. Biol. *6*, 477–487.

27. Rouet, P., Raguenez, G., Tronche, F., Yaniv, M., N'Guyen, C., and Salier, J.P. (1992). A potent enhancer made of clustered liver-specific elements in the transcription control sequences of human alpha 1-microglobulin/bikunin gene. J. Biol. Chem. *267*, 20765–20773.

28. Johnston, J.M., Denning, G., Moot, R., Whitehead, D., Shields, J., Le Doux, J.M., Doering, C.B., and Spencer, H.T. (2014). High-throughput screening identifies compounds that enhance lentiviral transduction. Gene Ther. *21*, 1008–1020.

29. Simioni, P., Tormene, D., Tognin, G., Gavasso, S., Bulato, C., Iacobelli, N.P., Finn, J.D., Spiezia, L., Radu, C., and Arruda, V.R. (2009). X-linked thrombophilia with a mutant factor IX (factor IX Padua). N. Engl. J. Med. *361*, 1671–1675.

30. Cantore, A., Nair, N., Della Valle, P., Di Matteo, M., Màtrai, J., Sanvito, F., Brombin, C., Di Serio, C., D'Angelo, A., Chuah, M., et al. (2012). Hyperfunctional coagulation factor IX improves the efficacy of gene therapy in hemophilic mice. Blood *120*, 4517–4520.

31. Clément, N., and Grieger, J.C. (2016). Manufacturing of recombinant adeno-associated viral vectors for clinical trials. Mol. Ther. Methods Clin. Dev. *3*, 16002.

32. Ayuso, E. (2016). Manufacturing of recombinant adeno-associated viral vectors: new technologies are welcome. Mol. Ther. Methods Clin. Dev. *3*, 15049.

33. Juven-Gershon, T., Cheng, S., and Kadonaga, J.T. (2006). Rational design of a super core promoter that enhances gene expression. Nat. Methods *3*, 917–922.

34. Nair, N., Rincon, M.Y., Evens, H., Sarcar, S., Dastidar, S., Samara-Kuko, E., Ghandeharian, O., Man Viecelli, H., Thöny, B., De Bleser, P., et al. (2014). Computationally designed liver-specific transcriptional modules and hyperactive factor IX improve hepatic gene therapy. Blood *123*, 3195–3199.

35. Fuda, N.J., Ardehali, M.B., and Lis, J.T. (2009). Defining mechanisms that regulate RNA polymerase II transcription in vivo. Nature *461*, 186–192.

36. Gebhardt, J.C., Suter, D.M., Roy, R., Zhao, Z.W., Chapman, A.R., Basu, S., Maniatis, T., and Xie, X.S. (2013). Single-molecule imaging of transcription factor binding to DNA in live mammalian cells. Nat. Methods *10*, 421–426.

37. Doering, C.B., Healey, J.F., Parker, E.T., Barrow, R.T., and Lollar, P. (2002). High level expression of recombinant porcine coagulation factor VIII. J. Biol. Chem. *277*, 38345–38349.

38. Akashi, H. (1994). Synonymous codon usage in Drosophila melanogaster: natural selection and translational accuracy. Genetics *136*, 927–935.

39. Drummond, D.A., and Wilke, C.O. (2009). The evolutionary consequences of erroneous protein synthesis. Nat. Rev. Genet. *10*, 715–724.

40. Shah, P., and Gilchrist, M.A. (2010). Effect of correlated tRNA abundances on translation errors and evolution of codon usage bias. PLoS Genet. *6*, e1001128.

41. Stoletzki, N., and Eyre-Walker, A. (2007). Synonymous codon usage in *Escherichia coli*: selection for translational accuracy. Mol. Biol. Evol. *24*, 374–381.

42. Mauro, V.P., and Chappell, S.A. (2014). A critical analysis of codon optimization in human therapeutics. Trends Mol. Med. *20*, 604–613.

43. Pavon-Eternod, M., Gomes, S., Geslain, R., Dai, Q., Rosner, M.R., and Pan, T. (2009). tRNA over-expression in breast cancer and functional consequences. Nucleic Acids Res. *37*, 7268–7280.

44. Winter, A.G., Sourvinos, G., Allison, S.J., Tosh, K., Scott, P.H., Spandidos, D.A., and White, R.J. (2000). RNA polymerase III transcription factor TFIIIC2 is overexpressed in ovarian tumors. Proc. Natl. Acad. Sci. USA *97*, 12619–12624.

45. Ancuta, P., Liu, K.Y., Misra, V., Wacleche, V.S., Gosselin, A., Zhou, X., and Gabuzda, D. (2009). Transcriptional profiling reveals developmental relationship and distinct biological functions of CD16+ and CD16- monocyte subsets. BMC Genomics *10*, 403.

46. Stothard, P. (2000). The sequence manipulation suite: JavaScript programs for analyzing and formatting protein and DNA sequences. Biotechniques *28*, 1102, 1104.

47. Harte, R.A., Farrell, C.M., Loveland, J.E., Suner, M.M., Wilming, L., Aken, B., Barrell, D., Frankish, A., Wallin, C., Searle, S., et al. (2012). Tracking and coordinating an international curation effort for the CCDS Project. Database (Oxford) *2012*, bas008.

48. Chan, P.P., and Lowe, T.M. (2009). GtRNAdb: a database of transfer RNA genes detected in genomic sequence. Nucleic Acids Res. *37*, D93–D97.

49. Bi, L., Lawler, A.M., Antonarakis, S.E., High, K.A., Gearhart, J.D., and Kazazian, H.H., Jr. (1995). Targeted disruption of the mouse factor VIII gene produces a model of haemophilia A. Nat. Genet. *10*, 119–121.

**OMTM, Volume *9***

## Supplemental Information

# Target-Cell-Directed Bioengineering Approaches for Gene Therapy of Hemophilia A

**Harrison C. Brown, Philip M. Zakas, Stephan N. George, Ernest T. Parker, H. Trent Spencer, and Christopher B. Doering**

**Supplementary Figure 1**

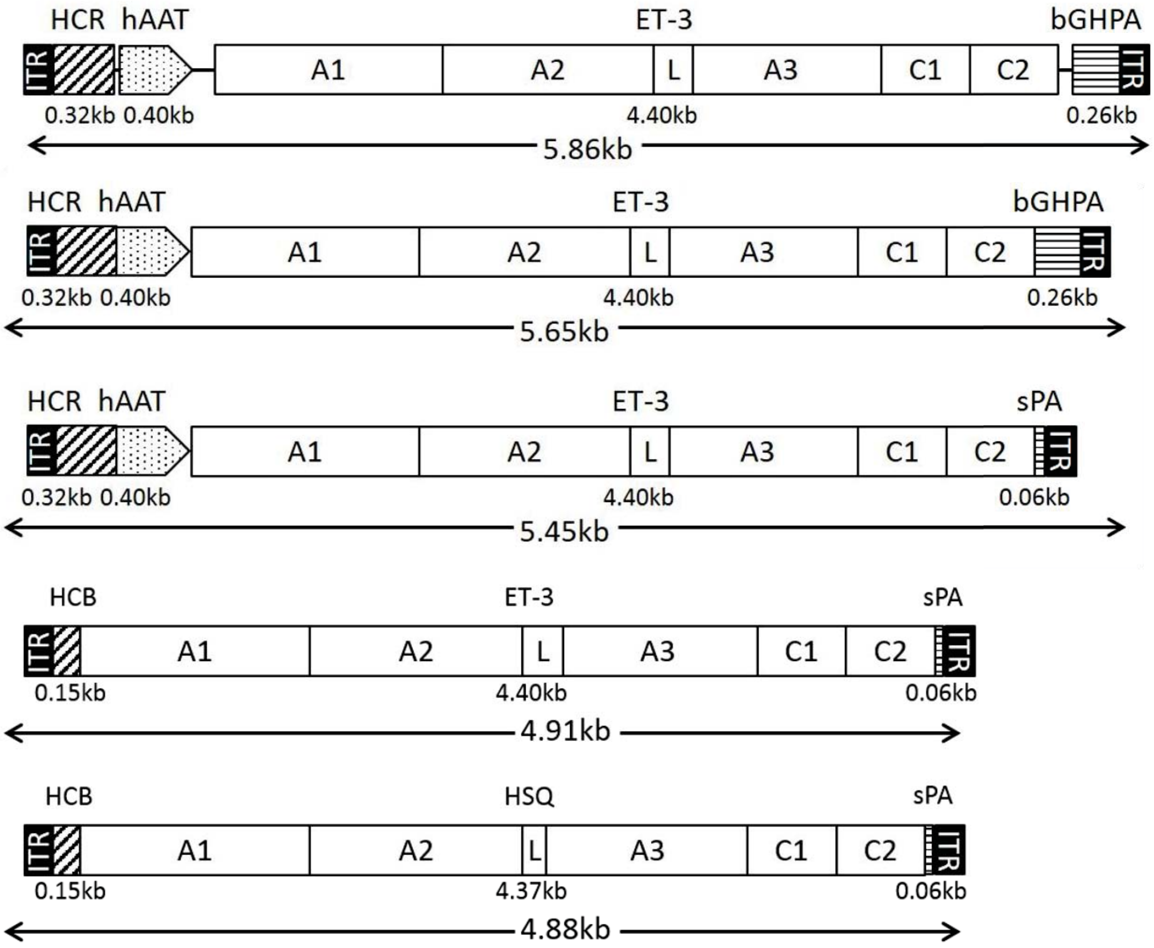


**AAV transgene designs:** To-scale vector schematics show the discrete elements and size of the resulting AAV transgene designs.

## Promoter Sequences

The promoters were synthesized *de novo* and cloned into an expression plasmid driving the expression of coagulation factor VIII. Factor VIII activity was measured 48 hours after transfection by one-stage clot assay. As a comparator, the hybrid liver promoter (HLP) is used in this and other experiments. HLP represents one of the shortest yet most powerful liver-directed promoters described to date.

*HLP reference:* Therapeutic levels of FVIII following a single peripheral vein administration of rAAV vector encoding a novel human factor VIII variant. McIntosh J. Blood. 2013 Apr 25;121(17):3335-44. doi: 10.1182/blood-2012-10-462200. Epub 2013 Feb 20.

### Native Sequence Elements

ABP element
5'GTTAATTTTTAAAAAGCAGTCAAAAGTCCAAGTGGCCCTTGCGAGCATTTACTCTCTCTGTTTGCTCTGGTTAATAATCTCAGGAGCACAAACA 3'

GTTA HNF-1-1 transcription factor binding site
HNF-4 transcription factor binding site
HNF-3a transcription factor binding site
HNF1-2 transcription factor binding site
HNF-3-2 transcription factor binding site

*Reference: A potent enhancer made of clustered liver-specific elements in the transcription control sequences of human alpha 1-microglobulin/bikunin gene. Rouet P. J Biol Chem. 1992 Oct 15;267(29):20765-73.*

HP1 element
5'GTTAATAATTTTC3'

*Reference: Hepatocyte-specific promoter element HP1 of the Xenopus albumin gene interacts with transcriptional factors of mammalian hepatocytes. Schorpp M. J Mol Biol. 1988 Jul 20;202(2):307-20.*

AFP element
5'AGTCATATGTTTGCTCACTGAAGGTTACTAGTTAACAGGCATCCCTTAAACAGGA3'

*Reference: Multiple regulatory elements in the intergenic region between the alpha-fetoprotein and albumin genes. Godbout R, Mol Cell Biol. 1986 Feb;6(2):477-87.*

SynO element
5'GAGGTTAATAATTTTCCAGATCTCTCTGAGCAATAGTATAA3'

HP1 binding element

*Reference: Liver cell specific gene transcription in vitro: the promoter elements HP1 and TATA box are necessary and sufficient to generate a liver-specific promoter. G U Ryffel Nucleic Acids Res. 1989 Feb 11; 17(3): 939–953.*

HNF1a transcription factor binding site (liver-directed transcription factor)
5'GTTAATCATTAA3'

SP1 binding site (liver-directed transcription factor)
5'TGGGCGGAGT3'

**Modifications to native sequences**

Transcription Start Site
5'GCCAGCAGCAGCCTGACCACATCTCATCCTC3'

CATC GC rich spacer
Transcription start site

Transcription start site (contains a 23 contains a GC rich spacer immediately after the TATA box for optimal spacing and a transcription start motif immediately after the spacer)

ABPshort
GTTAATTTTTGTGGCCCTTGCGATGTTTGCTCTGGTTAATAATCTCAGGACAAACA

GTTA HNF-1-1 transcription factor binding site
HNF-4 transcription factor binding site
HNF-3a transcription factor binding site
HNF1-2 transcription factor binding site
HNF-3-2 transcription factor binding site

**Complete Promoter Sequences**

HNF1-shortABP-SynO-TSS (designated the Hepatic Combinitoral Bundle, or HCB)
5'GTTAATCATTAAGTCGTTAATTTTTGTGGCCCTTGCGATGTTTGCTCTGGTTAATAATCTCAGGACAAACAGAGGT TAATAATTTTCCAGATCTCTCTGAGCAATAGTATAAAAGGCCAGCAGCAGCCTGACCACATCTCATCCTC3'

HNF1a
shortABP
SynO
CCTC TSS

ABPshort-HP1-AFP-TSS
5'GTTAATTTTTGTGGCCCTTGCGATGTTTGCTCTGGTTAATAATCTCAGGACAAACATACATTTTCAGTCATATGTTT GCTCACTGAAGGTTACTAGTTAACAGGCATCCCTTAAACAGGATATAAAAGGCCAGCAGCAGCCTGACCACATCTC ATCCTC3'

shortABP
HP1

AFP
TSS

ABPnat-HP1-AFP-TSS
5'GTTAATTTTTAAAAAGCAGTCAAAAGTCCAAGTGGCCCTTGCGAGCATTTACTCTCTCTGTTTGCTCTGGTTAATA
ATCTCAGGAGCACAAACAGAGGTTAATAATTTTCAGTCATATGTTTGCTCACTGAAGGTTACTAGTTAACAGGCAT
CCCTTAAACAGGATATAAAAGGCCAGCAGCAGCCTGACCACATCTCATCCTC3'

ABP enhancer
HP1
AFP
Transcription start site

HNF1-ABP-SynO
5'GTTAATCATTAAGTCGTTAATTTTTAAAAAGCAGTCAAAAGTCCAAGTGGCCCTTGCGAGCATTTACTCTCTCTGT
TTGCTCTGGTTAATAATCTCAGGAGCACAAACAGAGGTTAATAATTTTCCAGATCTCTCTGAGCAATAGTATAAAA3

HNF1 transcription factor binding site
ABP enhancer
SynO

SP1-ABP-SynO
5'TGGGCGGAGTGTCGTTAATTTTTAAAAAGCAGTCAAAAGTCCAAGTGGCCCTTGCGAGCATTTACTCTCTCTGTT
TGCTCTGGTTAATAATCTCAGGAGCACAAACAGAGGTTAATAATTTTCCAGATCTCTCTGAGCAATAGTATAAAA3'

HNF1 transcription factor binding site
ABP enhancer
SynO

ABP-SynO
5'GTTAATTTTTAAAAAGCAGTCAAAAGTCCAAGTGGCCCTTGCGAGCATTTACTCTCTCTGTTTGCTCTGGTTAATA
ATCTCAGGAGCACAAACAGAGGTTAATAATTTTCCAGATCTCTCTGAGCAATAGTATAAAA3'

**ABP element**
**SynO element**

ABP-HP1-AFP
5'GTTAATTTTTAAAAAGCAGTCAAAAGTCCAAGTGGCCCTTGCGAGCATTTACTCTCTCTGTTTGCTCTGGTTAATA
ATCTCAGGAGCACAAACAGAGGTTAATAATTTTCAGTCATATGTTTGCTCACTGAAGGTTACTAGTTAACAGGCAT
CCCTTAAACAGGATATAAAA3'

**ABP element**
**HP1 element**
**AFP element**

## FVIII sequences

An53-HCO

ATGCAGATTGAGCTGTCCACTTGCTTTTTCCTGTGCCTGCTGCAGTTTTCATTTTCCGCCACTAGAAGATACTACCTG
GGGGCTGTCGAACTGTCCTGGGATTACATGCAGTCCGACCTGCTGTCTGAGCTGCATGTGGACACCCGATTTCCAC
CTCGCGTCCCACGAAGCTTCCCCTTTAATACATCCGTGATGTACAAGAAAACTGTGTTCGTCGAGTTCACCGATCAC
CTGTTCAACATCGCAAAGCCCCGGCCACCCTGGATGGGACTGCTGGGCCCTACCATCAGAGCCGAGGTGTACGAC
ACCGTGGTCATTACACTGAAAAACATGGCAAGTCACCCCGTGTCACTGCATGCCGTGGGAGTCTCCTACTGGAAG
GCATCTGAAGGCGCCGAGTATGACGATCAGACTAGTCAGAGAGAAAAAGAGGACGATAAGGTGTTTCCCGGAGA
ATCTCATACCTATGTGTGGCAGGTCCTGAAGGAGAATGGCCCTATGGCCAGCGACCCTCCATGCCTGACCTACTCC
TATCTGTCTCACGTGGACCTGGTCAAAGATCTGAACTCCGGGCTGATCGGAGCCCTGCTGGTGTGTCGGGAAGGA
TCTCTGGCTAAGGAGAGAACCCAGACACTGCATCAGTTCGTGCTGCTGTTCGCTGTCTTTGACGAAGGCAAAAGTT
GGCACTCAGAGACAAAGGATTCCCTGACTCAGGCAATGGACTCTGCCAGTGCTAGGGCATGGCCAAAAATGCACA
CCGTGAACGGCTACGTCAATAGAAGCCTGCCAGGACTGATCGGATGCCACAGGAAGTCCGTGTATTGGCATGTCA
TCGGCATGGGGACCACACCAGAAGTCCACTCTATTTTCCTGGAGGGACATACATTTCTGGTGCGGAATCACAGACA
GGCTAGCCTGGAGATCTCCCCCATTACCTTCCTGACAGCACAGACTCTGCTGATGGATCTGGGCCAGTTCCTGCTG
TTTTGCCACATCAGCTCCCACCAGCATGATGGGATGGAGGCCTACGTGAAAGTCGACAGCTGTCCAGAGGAACCC
CAGCTGAGGATGAAGAACAATGAGGAAGAGGAAGACTACGACGATGACCTGTATGACAGCGAGATGGATGTGG
TCCGATTCGATGACGATAACTCACCCCCTTTTATCCAGATTAGAAGCGTCGCCAAGAAACACCCTAAGACTTGGGT
GCATTACATCGCCGCTGAGGAAGAGGACTGGGATTATGCTCCTTCCGTGCTGACCCCAGACGATCGCAGCTACAA
ATCCCAGTATCTGAACAATGGCCCTCAGAGGATTGGGCGCAAGTACAAGAAAGTGAGGTTCATGGCTTATACCGA
TGAAACCTTCAAGACTCGCGAAGCAATCCAGTACGAGTCCGGAATTCTGGGCCCACTGCTGTATGGGGAAGTGGG
AGACACCCTGCTGATCATTTTCAAGAACCAGGCCTCTAGGCCCTACAATATCTATCCTCATGGCATTACAGATGTGT
CTCCCCTGCACAGTGGACGCCTGCCTAAGGGCGTGAAACACCTGAAGGACCTGCCTATCCTGCCAGGGGAAATTT
TTAAGTACAAATGGACTGTGACCGTCGAGGATGGACCAACTAAGAGCGACCCCAGGTGCCTGACCCGCTACTATT
CTAGTTTCATCAATCTGGAAAGAGATCTGGCAAGCGGACTGATCGGACCACTGCTGATTTGTTACAAAGAGTCCGT
GGATCAGCGAGGCAACCAGATGATGTCTGACAAGCGGAATGTGATCCTGTTCTCAGTCTTTGACGAAAACCGCAG
CTGGTATCTGACCGAGAACATGCAGCGATTCCTGCCAAATGCAGCAGGAGTGCAGCCACAGGATCCTGAGTTTCA
GGCTAGTAACATCATGCATTCAATTAATGGCTACGTGTTCGACTCACTGCAGCTGAGCGTGTGTCTGCACGAGGTC
GCTTACTGGTATATCCTGAGCGTCGGAGCACAGACAGATTTCCTGTCCGTGTTCTTTTCTGGCTACACTTTTAAGCA
TAAAATGGTGTATGAGGACACACTGACTCTGTTCCCTTTTTCCGGCGAAACCGTCTTTATGTCTATGGAGAATCCAG
GGCTGTGGGTGCTGGGATGCCACAACTCCGATTTCCGGAATAGAGGAATGACTGCCCTGCTGAAAGTGTCAAGCT
GTGACCGGAACACCGGCGACTACTATGAAGATACATACGAGGACATCCCAACTTATCTGCTGTCTGAAAACAATGT
GATTGAGCCCAGAAGCTTCAGCCAGAATCCACCCGTGCTGAAGCGACACCAGCGGGAAATCACCCTGACTACCCT
GCAGTCAGAGCAGGAAGAGATTGATTACGACGATACCATCAGCATTGAAACAAAAAGGGAGGACTTCGATATCTA
TGGGGAAGACGAGAACCAGGGACCTCGCTCCTTCCAGAAGAGGACACGCCATTACTTTATTGCTGCAGTGGAGAG
GCTGTGGGATTATGGGATGTCCCGCTCTCCCCACGTCCTGCGAAATCGGGCCCAGAGTGGATCAGTGCCTCAGTTC
AAGAAAGTGGTCTTCCAGGAGTTTACTGACGGGAGCTTTACCCAGCCTCTGTACCGGGGAGAACTGAACGAGCAC
CTGGGACTGCTGGGCCCATATATCAGAGCAGAAGTGGAGGATAACATTATGGTCACCTTCAAGAATCAGGCCAGT
CGGCCCTACTCATTTTATTCCTCTCTGATCAGCTACGAAGAGGACCAGCGCCAGGGCGCAGAACCACGAAAAAACT
TCGTGAAGCCCAATGAGACCAAAACATACTTTTGGAAGGTGCAGCACCATATGGCTCCTACAAAAGACGAATTCG
ATTGCAAGGCCTGGGCTTATTTTAGTGACGTGGATCTGGAGAAGGACATGCACTCAGGGCTGATCGGACCTCTGC
TGATTTGTCATACTAACACCCTGAATCCAGCACACGGACGACAGGTGACAGTCCAGGAATTCGCTCTGTTCTTTAC
AATCTTCGATGAGACTAAGAGCTGGTACTTCACTGAAAACATGGAGAGAAATTGCAGGGCCCCTTGTAATATCCA
GATGGAAGACCCAACATTCAAGGAGAACTACAGATTTCATGCTATTAATGGCTATGTGATGGATACTCTGCCAGG

GCTGGTCATGGCACAGGACCAGAGAATCAGGTGGTACCTGCTGTCTATGGGGAGTAACGAGAATATCCACAGCAT
TCATTTCTCCGGACACGTGTTTACTGTCAGGAAGAAAGAAGAGTATAAAATGGCCGTGTACAACCTGTATCCAGGC
GTGTTCGAAACCGTCGAGATGCTGCCAAGCAAGGCAGGAATCTGGCGAGTGGAATGCCTGATTGGCGAGCACCT
GCATGCTGGGATGAGTACCCTGTTTCTGGTGTACTCAAAACAGTGTCAGACACCTCTGGGAATGGCATCTGGCCAT
ATCCGGGATTTCCAGATTACCGCAAGTGGACAGTACGGACAGTGGGCTCCAAAGCTGGCAAGACTGCACTATAGC
GGCTCCATCAACGCCTGGTCTACAAAAGAGCCCTTTAGTTGGATTAAGGTGGACCTGCTGGCCCCTATGATCATTC
ATGGCATCAAAACTCAGGGGGCTAGGCAGAAGTTCAGTTCACTGTACATCAGCCAGTTTATCATCATGTACTCCCT
GGATGGGAAGAAATGGCAGACCTACCGCGGGAATAGCACAGGAACTCTGATGGTGTTCTTTGGAAACGTCGACA
GCTCCGGCATCAAGCACAACATTTTCAATCCTCCAATCATTGCCCGCTACATCCGACTGCACCCCACCCATTATTCAA
TTCGAAGCACACTGCGGATGGAACTGATGGGCTGCGATCTGAACTCTTGTAGTATGCCTCTGGGGATGGAGTCTA
AGGCCATCAGTGACGCTCAGATTACCGCATCTAGTTACTTCACCAATATGTTTGCCACATGGTCACCAAGCCAGGC
TAGGCTGCACCTGCAGGGAAGAACAAACGCCTGGAGGCCTCAGGTGAACAATCCAAAGGAGTGGCTGCAGGTGG
ATTTCCAGAAAACTATGAAGGTCACCGGAATCACAACTCAGGGCGTGAAATCACTGCTGACCAGCATGTATGTGA
AGGAGTTTCTGATTTCAAGCTCCCAGGACGGCCACCATTGGACACTGTTCCTGCAGAACGGGAAGGTGAAAGTCT
TCCAGGGAAATCAGGATTCTTTTACACCAGTGGTCAACAGTCTGGACCCCCCTCTGCTGACTCGGTACCTGAGAAT
CCACCCCCAGAGCTGGGTCCATCAGATTGCACTGCGACTGGAAGTGCTGGGATGCGAGGCACAGCAGCTGTATTG
A

An53-LCO
ATGCAGATTGAGCTGAGCACCTGCTTCTTCCTGTGCCTGCTGCAGTTCTCATTCTCTGCCACCAGGAGATACTACCT
GGGCGCCGTGGAGCTGAGCTGGGACTACATGCAGTCTGACCTGCTGTCTGAGCTGCATGTGGACACCAGGTTCCC
CCCCAGAGTGCCCCGAAGCTTCCCCTTCAACACCAGCGTGATGTACAAGAAGACCGTGTTCGTGGAGTTCACTGAC
CACCTGTTCAACATCGCCAAGCCCAGGCCCCCCTGGATGGGCCTGCTGGGCCCCACCATCAGAGCCGAGGTGTAC
GACACCGTGGTCATCACCCTGAAGAACATGGCCAGCCACCCCGTCTCCCTGCACGCCGTGGGGGTGAGCTACTGG
AAGGCCTCTGAGGGCGCCGAGTACGACGACCAGACCAGCCAGAGGGAGAAGGAGGACGACAAGGTGTTCCCTG
GGGAAAGCCACACCTACGTGTGGCAGGTCCTGAAGGAGAACGGCCCCATGGCCTCTGACCCCCCATGCCTGACCT
ACAGCTACCTGAGCCACGTGGACCTGGTGAAGGACCTGAACTCTGGCCTGATTGGGGCCCTGCTGGTGTGCAGGG
AGGGCAGCCTGGCCAAGGAGAGAACCCAGACCCTGCACCAGTTCGTGCTGCTGTTCGCCGTGTTCGACGAGGGC
AAGAGCTGGCACTCTGAAACCAAGGATAGCCTGACTCAGGCCATGGACTCTGCCTCTGCCAGGGCCTGGCCCAAG
ATGCACACCGTCAACGGCTACGTCAACAGGAGCCTGCCTGGCCTGATTGGCTGCCACAGGAAGAGCGTGTACTGG
CATGTGATCGGCATGGGCACCACCCCTGAGGTGCACAGCATCTTCCTGGAGGGCCACACCTTCCTGGTCAGGAAC
CACAGGCAGGCCAGCCTGGAGATCAGCCCCATCACCTTCCTGACCGCCCAGACCCTGCTGATGGACCTGGGCCAG
TTCCTGCTGTTCTGCCACATCTCCAGCCACCAGCACGACGGCATGGAGGCCTACGTGAAAGTGGACAGCTGCCCTG
AGGAGCCCCAGCTGAGGATGAAGAACAACGAGGAGGAGGAGGACTATGATGACGACCTGTATGACAGCGAGAT
GGACGTGGTCAGGTTCGACGACGACAACAGCCCCCCTTTCATCCAGATCAGGAGCGTGGCCAAGAAGCACCCCAA
GACCTGGGTGCACTACATCGCTGCTGAGGAGGAGGACTGGGACTATGCCCCCTCCGTGCTGACCCCTGATGACAG
GAGCTACAAGAGCCAGTACCTGAACAATGGCCCCCAGAGGATTGGCAGGAAGTACAAGAAAGTCAGGTTCATGG
CCTACACTGATGAAACCTTCAAGACCAGGGAGGCCATCCAGTACGAGTCTGGCATCCTGGGCCCCCTGCTGTACG
GGGAGGTGGGGGACACCCTGCTGATCATCTTCAAGAACCAGGCCAGCAGGCCCTACAACATCTACCCCCATGGCA
TCACCGACGTGAGCCCCCTGCACAGCGGAAGGCTGCCTAAGGGGGTGAAGCACCTGAAAGACCTGCCCATCCTGC
CTGGGGAGATCTTCAAGTACAAGTGGACTGTGACTGTGGAGGACGGCCCCACCAAGAGCGACCCCAGGTGCCTG
ACCAGATACTACAGCAGCTTCATCAACCTGGAGAGGGACCTGGCCTCTGGCCTGATTGGCCCCCTGCTGATCTGCT
ACAAGGAGTCTGTGGACCAGAGGGGCAACCAGATGATGAGCGACAAGAGGAACGTGATCCTGTTCTCTGTCTTC
GACGAGAACAGGAGCTGGTACCTGACCGAGAACATGCAGAGGTTCCTGCCCAACGCAGCTGGGGTGCAGCCACA
GGACCCCGAGTTCCAGGCCAGCAACATCATGCACAGCATCAATGGCTACGTGTTCGACAGCCTGCAGCTGAGCGT
GTGCCTGCACGAGGTGGCCTACTGGTACATCCTGAGCGTCGGCGCCCAGACCGACTTCCTGAGCGTGTTCTTCTCT
GGCTACACCTTCAAGCACAAGATGGTGTATGAGGACACCCTGACCCTGTTCCCCTTCAGCGGGGAGACTGTCTTCA
TGAGCATGGAGAACCCTGGCCTGTGGGTGCTGGGCTGCCACAACAGCGACTTCAGGAACAGGGGCATGACTGCC

CTGCTGAAAGTCTCCAGCTGTGACCGGAACACCGGGGACTACTACGAGGACACATACGAGGACATCCCAACTTAC
CTGCTGAGCGAAAACAATGTGATCGAGCCCAGGAGCTTCTCTCAGAACCCCCCAGTGCTGAAGAGGCACCAGAGG
GAGATCACCCTGACCACCCTGCAGTCTGAGCAGGAGGAGATCGACTATGATGACACCATCAGCATTGAGACAAAG
AGGGAGGACTTCGACATCTACGGGGAGGACGAGAACCAGGGACCCAGGAGCTTCCAGAAGAGGACCAGGCACT
ACTTCATTGCTGCTGTGGAGAGGCTGTGGGACTATGGCATGTCCCGCAGCCCCCATGTGCTGAGGAACAGGGCCC
AGTCTGGCAGCGTGCCCCAGTTCAAGAAAGTCGTGTTCCAGGAGTTCACCGACGGCAGCTTCACCCAGCCCCTGTA
CAGAGGGGAGCTGAACGAGCACCTGGGCCTGCTGGGCCCCTACATCAGGGCCGAGGTGGAGGACAACATCATG
GTGACCTTCAAGAACCAGGCCAGCAGGCCCTACAGCTTCTACAGCAGCCTGATCAGCTACGAGGAGGACCAGAGG
CAGGGGGCTGAGCCCAGGAAGAACTTTGTGAAGCCCAATGAAACCAAGACCTACTTCTGGAAGGTGCAGCACCA
CATGGCCCCCACCAAGGACGAGTTCGACTGCAAGGCCTGGGCCTACTTCTCTGACGTGGACCTGGAGAAGGACAT
GCACTCTGGCCTGATTGGCCCCCTGCTGATTTGCCACACCAACACCCTGAACCCTGCCCATGGCAGGCAGGTGACT
GTGCAGGAGTTCGCCCTGTTCTTCACCATCTTCGATGAAACCAAGAGCTGGTACTTCACTGAGAACATGGAGAGG
AACTGCAGGGCCCCCTGCAACATCCAGATGGAGGACCCCACCTTCAAGGAGAACTACAGGTTCCATGCCATCAAT
GGCTACGTGATGGACACCCTGCCTGGCCTGGTCATGGCCCAGGACCAGAGGATCAGGTGGTATCTGCTGAGCATG
GGCAGCAACGAGAACATCCACAGCATCCACTTCTCTGGCCACGTGTTCACTGTGAGGAAGAAGGAGGAGTACAAG
ATGGCCGTGTACAACCTGTACCCTGGGGTGTTCGAAACCGTGGAGATGCTGCCCAGCAAGGCCGGCATCTGGAG
GGTGGAGTGCCTGATTGGGGAGCACCTGCACGCCGGCATGAGCACCCTGTTCCTGGTGTACAGCAAACAGTGCCA
GACCCCCCTGGGCATGGCCTCTGGCCACATCAGGGACTTCCAGATCACTGCCTCTGGCCAGTACGGCCAGTGGGC
CCCCAAGCTGGCCAGGCTGCACTACTCCGGAAGCATCAATGCCTGGAGCACCAAGGAGCCCTTCAGCTGGATCAA
AGTGGACCTGCTGGCCCCCATGATCATCCACGGCATCAAGACCCAGGGGGCCAGGCAGAAGTTCTCCAGCCTGTA
CATCAGCCAGTTCATCATCATGTACAGCCTGGACGGCAAGAAGTGGCAGACCTACAGGGGCAACAGCACCGGCAC
CCTGATGGTGTTCTTCGGCAACGTGGACAGCAGCGGCATCAAGCACAACATCTTCAACCCCCCCATCATCGCCAGA
TACATCAGGCTGCACCCCACCCACTACAGCATCAGGAGCACCCTGAGGATGGAGCTGATGGGCTGTGACCTGAAC
AGCTGCAGCATGCCCCTGGGCATGGAGAGCAAGGCCATCTCTGACGCCCAGATCACTGCCTCCAGCTACTTCACCA
ACATGTTTGCCACCTGGAGCCCCAGCCAGGCCAGGCTGCACCTGCAGGGCAGGACAAATGCCTGGAGGCCCCAG
GTCAACAACCCCAAGGAGTGGCTGCAGGTGGACTTCCAGAAGACCATGAAGGTGACTGGGATCACCACCCAGGG
GGTGAAGAGCCTGCTGACCAGCATGTACGTGAAGGAGTTCCTGATCTCCAGCAGCCAGGACGGCCACCATTGGAC
CCTGTTCCTGCAGAATGGCAAGGTGAAGGTGTTCCAGGGCAACCAGGACAGCTTCACCCCTGTGGTCAACAGCCT
GGACCCCCCCCTGCTGACCAGATACCTGAGGATCCACCCCCAGAGCTGGGTGCACCAGATCGCCCTGAGGCTGGA
GGTGCTGGGCTGTGAGGCCCAGCAGCTGTACTGA

<u>ET3-LCO with CpGs</u>
ATGCAGCTGGAACTGTCTACCTGTGTGTTTCTGTGTCTGCTGCCTCTGGGGTTTTCTGCT
ATCCGCCGCTACTATCTGGGAGCCGTGGAGCTGTCCTGGGACTACAGGCAGAGCGAGCTG
CTGAGAGAACTGCACGTGGATACCAGATTCCCAGCTACCGCTCCAGGAGCTCTGCCTCTG
GGCCCATCCGTGCTGTACAAGAAAACCGTCTTCGTGGAGTTTACCGACCAGCTGTTCAGC
GTGGCCAGGCCAAGACCACCTTGGATGGGACTGCTGGGACCAACCATCCAGGCTGAGGTG
TACGATACCGTGGTCGTGACCCTGAAAAACATGGCCTCCCATCCCGTGAGCCTGCACGCT
GTCGGGGTGTCCTTCTGGAAGTCCAGCGAGGGAGCCGAGTACGAAGACCATACCTCCCAG
CGCGAGAAAGAAGACGATAAGGTGCTGCCTGGCAAAAGCCAGACCTATGTCTGGCAGGTG
CTGAAGGAGAACGGACCAACCGCTAGCGACCCACCATGCCTGACCTACTCTTATCTGTCC
CACGTCGATCTGGTGAAGGACCTGAATTCCGGACTGATCGGAGCTCTGCTGGTGTGTAGA
GAGGGAAGCCTGACCAGAGAAAGAACCCAGAACCTGCATGAGTTCGTCCTGCTGTTCGCC
GTGTTTGACGAAGGGAAGAGCTGGCACTCTGCCCGCAATGACTCCTGGACCAGAGCTATG
GATCCAGCTCCTGCTAGAGCTCAGCCTGCTATGCACACCGTCAACGGCTACGTGAATCGG
TCTCTGCCAGGACTGATCGGCTGCCATAAGAAAAGCGTCTATTGGCACGTGATCGGAATG
GGCACCAGCCCCGAGGTGCATTCTATCTTCCTGGAAGGCCACACCTTTCTGGTCAGGCAC
CATAGACAGGCCTCTCTGGAGATCTCCCCTCTGACCTTCCTGACCGCTCAGACCTTTCTG

ATGGACCTGGGGCAGTTCCTGCTGTTTTGCCATATCTCTTCCCACCATCACGGAGGAATG
GAGGCTCACGTCAGGGTGGAATCCTGTGCTGAGGAACCACAGCTGAGAAGAAAGGCTGAT
GAGGAAGAGGACTACGACGATAACCTGTATGACAGCGATATGGACGTCGTGCGCCTGGAC
GGCGACGATGTCAGCCCTTTCATCCAGATCCGGTCTGTGGCCAAGAAACATCCAAAGACC
TGGGTCCACTACATCGCCGCTGAAGAGGAAGATTGGGACTATGCCCCCCTGGTGCTGGCT
CCTGACGATAGATCCTACAAAAGCCAGTATCTGAACAATGGGCCCCAGCGCATCGGACGG
AAGTACAAGAAAGTGAGGTTCATGGCCTATACCGACGAGACCTTTAAGACCAGAGAGGCT
ATCCAGCACGAATCCGGGATCCTGGGACCTCTGCTGTACGGCGAAGTGGGGGATACCCTG
CTGATCATCTTCAAGAACCAGGCCTCCAGGCCATACAATATCTATCCCCATGGCATCACC
GACGTGAGACCACTGTACAGCAGGAGACTGCCCAAGGGGGTCAAACACCTGAAGGATTTC
CCCATCCTGCCTGGAGAGATCTTTAAGTATAAATGGACCGTCACCGTGGAAGACGGGCCT
ACCAAGTCCGATCCACGCTGCCTGACCCGGTACTATAGCTCTTTCGTGAACATGGAGAGA
GACCTGGCTAGCGGACTGATCGGACCCCTGCTGATCTGTTACAAAGAGAGCGTGGACCAG
AGGGGCAACCAGATCATGTCTGATAAGAGAAATGTCATCCTGTTCTCCGTGTTTGACGAG
AACCGCAGCTGGTACCTGACCGAGAACATCCAGCGGTTCCTGCCAAATCCAGCTGGAGTG
CAGCTGGAGGACCCAGAATTTCAGGCTTCCAACATCATGCATAGCATCAATGGCTACGTG
TTCGATAGCCTGCAGCTGTCTGTCTGCCTGCACGAGGTGGCCTACTGGTATATCCTGTCC
ATCGGCGCTCAGACCGACTTCCTGTCCGTGTTCTTTAGCGGGTACACCTTTAAGCATAAA
ATGGTGTATGAGGATACCCTGACCCTGTTCCCCTTTTCTGGCGAGACCGTGTTCATGTCC
ATGGAAAACCCTGGCCTGTGGATCCTGGGGTGCCACAACAGCGACTTCAGGAATAGAGGA
ATGACCGCCCTGCTGAAAGTGTCCAGCTGTGATAAGAATACCGGCGATTACTATGAGGAC
TCTTACGAAGATATCTCCGCTTATCTGCTGAGCAAGAACAATGCCATCGAGCCCAGGTCT
TTCGCTCAGAACTCCAGACCTCCAAGCGCTTCTGCTCCTAAGCCACCTGTGCTGAGAAGA
CATCAGAGGGACATCTCCCTGCCTACCTTCCAGCCAGAGGAAGATAAAATGGACTACGAC
GATATCTTCAGCACCGAGACCAAGGGGGAAGATTTTGACATCTATGGAGAGGACGAAAAC
CAGGATCCAAGATCCTTCCAGAAGAGAACCAGACACTACTTTATCGCCGCTGTGGAGCAG
CTGTGGGACTATGGGATGTCCGAAAGCCCACGGGCCCTGAGGAACAGAGCTCAGAATGGA
GAGGTGCCCCGCTTCAAGAAAGTCGTGTTCCGGGAGTTTGCCGACGGCAGCTTTACCCAG
CCATCTTACAGGGGGGAGCTGAACAAGCATCTGGGGCTGCTGGGACCCTATATCAGAGCC
GAGGTCGAAGATAACATCATGGTGACCTTCAAGAATCAGGCTTCTCGCCCCTACTCCTTT
TATTCTTCCCTGATCTCCTACCCTGACGATCAGGAGCAGGGCGCCGAACCTAGGCACAAC
TTCGTGCAGCCAAATGAGACCAGAACCTACTTTTGGAAGGTGCAGCATCACATGGCTCCC
ACCGAGGATGAATTCGACTGCAAAGCTTGGGCCTATTTTTCCGATGTCGACCTGGAGAAG
GACGTGCATAGCGGCCTGATCGGGCCTCTGCTGATCTGTCGCGCCAACACCCTGAATGCT
GCTCACGGAAGACAGGTCACCGTGCAGGAGTTCGCTCTGTTCTTTACCATCTTTGACGAA
ACCAAGAGCTGGTACTTCACCGAGAACGTGGAAAGGAATTGCAGAGCCCCCTGTCATCTG
CAGATGGAGGACCCTACCCTGAAGGAAAACTACAGGTTCCACGCCATCAATGGATATGTC
ATGGATACCCTGCCCGGCCTGGTCATGGCTCAGAACCAGCGCATCCGGTGGTACCTGCTG
TCTATGGGATCCAACGAGAATATCCATAGCATCCACTTCTCTGGCCATGTCTTTTCCGTG
AGGAAGAAAGAGGAATACAAAATGGCCGTGTACAATCTGTATCCTGGGGTCTTCGAGACC
GTGGAAATGCTGCCAAGCAAAGTGGGAATCTGGAGAATCGAGTGCCTGATCGGCGAACAC
CTGCAGGCCGGGATGAGCACCACCTTCCTGGTGTACTCTAAGAAATGTCAGACCCCACTG
GGGATGGCCTCCGGACATATCCGCGACTTCCAGATCACCGCTAGCGGACAGTACGGACAG
TGGGCTCCAAAGCTGGCTAGACTGCACTATTCTGGCTCCATCAACGCCTGGTCTACCAAA
GAGCCATTCTCCTGGATCAAGGTGGACCTGCTGGCCCCCATGATCATCCACGGAATCAAA
ACCCAGGGCGCTAGGCAGAAGTTCAGCTCTCTGTACATCTCCCAGTTTATCATCATGTAT
AGCCTGGACGGGAAGAAATGGCAGACCTACAGAGGCAATTCCACCGGGACCCTGATGGTC
TTCTTTGGAAACGTGGATTCCAGCGGCATCAAGCACAACATCTTCAATCCACCCATCATC

GCCCGCTACATCCGGCTGCATCCTACCCACTATAGCATCAGGTCTACCCTGAGAATGGAG
CTGATGGGATGCGACCTGAACAGCTGTTCTATGCCACTGGGCATGGAGTCCAAGGCTATC
AGCGATGCCCAGATCACCGCTTCTTCCTACTTCACCAATATGTTTGCTACCTGGTCCCCA
AGCAAGGCTAGACTGCACCTGCAGGGAAGATCCAACGCTTGGAGACCCCAGGTGAACAAT
CCTAAGGAGTGGCTGCAGGTCGACTTCCAGAAAACCATGAAGGTCACCGGGGTGACCACC
CAGGGAGTGAAATCTCTGCTGACCTCCATGTACGTCAAGGAGTTCCTGATCAGCTCTTCC
CAGGACGGCCACCAGTGGACCCTGTTCTTTCAGAACGGCAAGGTCAAAGTGTTCCAGGGG
AATCAGGACTCTTTTACCCCCGTCGTGAACTCCCTGGATCCTCCACTGCTGACCAGGTAC
CTGAGAATCCATCCTCAGAGCTGGGTGCACCAGATCGCTCTGAGAATGGAGGTCCTGGGA
TGCGAAGCTCAGGACCTGTATTGA

<u>ET3-LCO CpGs removed</u>
ATGCAGCTGGAACTGTCTACCTGTGTGTTTCTGTGTCTGCTGCCTCTGGGGTTTTCTGCT
ATCAGGAGATACTATCTGGGAGCTGTGGAGCTGTCCTGGGACTACAGGCAGTCTGAGCTG
CTGAGAGAACTGCATGTGGATACCAGATTCCCAGCTACAGCTCCAGGAGCTCTGCCTCTG
GGCCCATCTGTGCTGTACAAGAAAACAGTCTTTGTGGAGTTTACAGACCAGCTGTTCTCT
GTGGCCAGGCCAAGACCACCTTGGATGGGACTGCTGGGACCAACCATCCAGGCTGAGGTG
TATGATACAGTGGTGGTGACCCTGAAAAACATGGCCTCCCATCCTGTGAGCCTGCATGCT
GTGGGGGTGTCCTTCTGGAAGTCCTCTGAGGGAGCTGAGTATGAAGACCATACCTCCCAG
AGGGAGAAAGAAGATGATAAGGTGCTGCCTGGCAAAAGCCAGACCTATGTCTGGCAGGTG
CTGAAGGAGAATGGACCAACTGCTTCTGACCCACCATGCCTGACCTACTCTTATCTGTCC
CATGTGGATCTGGTGAAGGACCTGAATTCTGGACTGATTGGAGCTCTGCTGGTGTGTAGA
GAGGGAAGCCTGACCAGAGAAAGAACCCAGAACCTGCATGAGTTTGTCCTGCTGTTTGCT
GTGTTTGATGAAGGGAAGAGCTGGCACTCTGCCAGGAATGACTCCTGGACCAGAGCTATG
GATCCAGCTCCTGCTAGAGCTCAGCCTGCTATGCACACAGTCAATGGCTATGTGAATAGG
TCTCTGCCAGGACTGATTGGCTGCCATAAGAAATCTGTCTATTGGCATGTGATTGGAATG
GGCACCAGCCCTGAGGTGCATTCTATCTTCCTGGAAGGCCACACCTTTCTGGTCAGGCAC
CATAGACAGGCCTCTCTGGAGATCTCCCCTCTGACCTTCCTGACAGCTCAGACCTTTCTG
ATGGACCTGGGGCAGTTCCTGCTGTTTTGCCATATCTCTTCCCACCATCATGGAGGAATG
GAGGCTCATGTCAGGGTGGAATCCTGTGCTGAGGAACCACAGCTGAGAAGAAAGGCTGAT
GAGGAAGAGGACTATGATGATAACCTGTATGACTCTGATATGGATGTGGTGAGGCTGGAT
GGGGATGATGTCAGCCCTTTCATCCAGATCAGGTCTGTGGCCAAGAAACATCCAAAGACC
TGGGTCCACTACATTGCTGCTGAAGAGGAAGATTGGGACTATGCCCCCCTGGTGCTGGCT
CCTGATGATAGATCCTACAAAAGCCAGTATCTGAACAATGGGCCCCAGAGGATTGGAAGG
AAGTACAAGAAAGTGAGGTTCATGGCCTATACAGATGAGACCTTTAAGACCAGAGAGGCT
ATCCAGCATGAATCTGGGATCCTGGGACCTCTGCTGTATGGAGAAGTGGGGGATACCCTG
CTGATCATCTTCAAGAACCAGGCCTCCAGGCCATACAATATCTATCCCCATGGCATCACA
GATGTGAGACCACTGTACAGCAGGAGACTGCCCAAGGGGGTCAAACACCTGAAGGATTTC
CCCATCCTGCCTGGAGAGATCTTTAAGTATAAATGGACAGTCACAGTGGAAGATGGGCCT
ACCAAGTCTGATCCAAGGTGCCTGACCAGATACTATAGCTCTTTTGTGAACATGGAGAGA
GACCTGGCTTCTGGACTGATTGGACCCCTGCTGATCTGTTACAAAGAGTCTGTGGACCAG
AGGGGCAACCAGATCATGTCTGATAAGAGAAATGTCATCCTGTTCTCTGTGTTTGATGAG
AACAGGAGCTGGTACCTGACAGAGAACATCCAGAGGTTCCTGCCAAATCCAGCTGGAGTG
CAGCTGGAGGACCCAGAATTTCAGGCTTCCAACATCATGCATAGCATCAATGGCTATGTG
TTTGATAGCCTGCAGCTGTCTGTCTGCCTGCATGAGGTGGCCTACTGGTATATCCTGTCC
ATTGGAGCTCAGACAGACTTCCTGTCTGTGTTCTTTAGTGGGTACACCTTTAAGCATAAA
ATGGTGTATGAGGATACCCTGACCCTGTTCCCCTTTTCTGGGGAGACAGTGTTCATGTCC

ATGGAAAACCCTGGCCTGTGGATCCTGGGGTGCCACAACTCTGACTTCAGGAATAGAGGA
ATGACAGCCCTGCTGAAAGTGTCCAGCTGTGATAAGAATACAGGGGATTACTATGAGGAC
TCTTATGAAGATATCTCTGCTTATCTGCTGAGCAAGAACAATGCCATTGAGCCCAGGTCT
TTTGCTCAGAACTCCAGACCTCCATCTGCTTCTGCTCCTAAGCCACCTGTGCTGAGAAGA
CATCAGAGGGACATCTCCCTGCCTACCTTCCAGCCAGAGGAAGATAAAATGGACTATGAT
GATATCTTCAGCACAGAGACCAAGGGGGAAGATTTTGACATCTATGGAGAGGATGAAAAC
CAGGATCCAAGATCCTTCCAGAAGAGAACCAGACACTACTTTATTGCTGCTGTGGAGCAG
CTGTGGGACTATGGGATGTCTGAAAGCCCAAGGGCCCTGAGGAACAGAGCTCAGAATGGA
GAGGTGCCCAGATTCAAGAAAGTGGTGTTCAGAGAGTTTGCTGATGGCAGCTTTACCCAG
CCATCTTACAGGGGGGAGCTGAACAAGCATCTGGGGCTGCTGGGACCCTATATCAGAGCT
GAGGTGGAAGATAACATCATGGTGACCTTCAAGAATCAGGCTTCTAGGCCCTACTCCTTT
TATTCTTCCCTGATCTCCTACCCTGATGATCAGGAGCAGGGAGCTGAACCTAGGCACAAC
TTTGTGCAGCCAAATGAGACCAGAACCTACTTTTGGAAGGTGCAGCATCACATGGCTCCC
ACAGAGGATGAATTTGACTGCAAAGCTTGGGCCTATTTTTCTGATGTGGACCTGGAGAAG
GATGTGCATTCTGGCCTGATTGGGCCTCTGCTGATCTGTAGGGCCAACACCCTGAATGCT
GCTCATGGAAGACAGGTCACAGTGCAGGAGTTTGCTCTGTTCTTTACCATCTTTGATGAA
ACCAAGAGCTGGTACTTCACAGAGAATGTGGAAAGGAATTGCAGAGCCCCCTGTCATCTG
CAGATGGAGGACCCTACCCTGAAGGAAAACTACAGGTTCCATGCCATCAATGGATATGTC
ATGGATACCCTGCCTGGCCTGGTCATGGCTCAGAACCAGAGGATCAGATGGTACCTGCTG
TCTATGGGATCCAATGAGAATATCCATAGCATCCACTTCTCTGGCCATGTCTTTTCTGTG
AGGAAGAAAGAGGAATACAAAATGGCTGTGTACAATCTGTATCCTGGGGTCTTTGAGACA
GTGGAAATGCTGCCAAGCAAAGTGGGAATCTGGAGAATTGAGTGCCTGATTGGGGAACAC
CTGCAGGCTGGGATGAGCACCACCTTCCTGGTGTACTCTAAGAAATGTCAGACCCCACTG
GGGATGGCCTCTGGACATATCAGGGACTTCCAGATCACAGCTTCTGGACAGTATGGACAG
TGGGCTCCAAAGCTGGCTAGACTGCACTATTCTGGCTCCATCAATGCCTGGTCTACCAAA
GAGCCATTCTCCTGGATCAAGGTGGACCTGCTGGCCCCCATGATCATCCATGGAATCAAA
ACCCAGGGAGCTAGGCAGAAGTTCAGCTCTCTGTACATCTCCCAGTTTATCATCATGTAT
AGCCTGGATGGGAAGAAATGGCAGACCTACAGAGGCAATTCCACTGGGACCCTGATGGTC
TTCTTTGGAAATGTGGATTCCTCTGGCATCAAGCACAACATCTTCAATCCACCCATCATT
GCCAGGTACATCAGGCTGCATCCTACCCACTATAGCATCAGGTCTACCCTGAGAATGGAG
CTGATGGGATGTGACCTGAACAGCTGTTCTATGCCACTGGGCATGGAGTCCAAGGCTATC
TCTGATGCCCAGATCACAGCTTCTTCCTACTTCACCAATATGTTTGCTACCTGGTCCCCA
AGCAAGGCTAGACTGCACCTGCAGGGAAGATCCAATGCTTGGAGACCCCAGGTGAACAAT
CCTAAGGAGTGGCTGCAGGTGGACTTCCAGAAAACCATGAAGGTCACAGGGGTGACCACC
CAGGGAGTGAAATCTCTGCTGACCTCCATGTATGTCAAGGAGTTCCTGATCAGCTCTTCC
CAGGATGGCCACCAGTGGACCCTGTTCTTTCAGAATGGCAAGGTCAAAGTGTTCCAGGGG
AATCAGGACTCTTTTACCCCAGTGGTGAACTCCCTGGATCCTCCACTGCTGACCAGGTAC
CTGAGAATCCATCCTCAGAGCTGGGTGCACCAGATTGCTCTGAGAATGGAGGTCCTGGGA
TGTGAAGCTCAGGACCTGTATTGA

ET3-NoCo
ATGCAGCTAGAGCTCTCCACCTGTGTCTTTCTGTGTCTCTTGCCACTCGGCTTTAGTGCCATCAGGAGATACTACCT
GGGCGCAGTGGAACTGTCCTGGGACTACCGGCAAAGTGAACTCCTCCGTGAGCTGCACGTGGACACCAGATTTCC
TGCTACAGCGCCAGGAGCTCTTCCGTTGGGCCCGTCAGTCCTGTACAAAAAGACTGTGTTCGTAGAGTTCACGGAT
CAACTTTTCAGCGTTGCCAGGCCCAGGCCACCATGGATGGGTCTGCTGGGTCCTACCATCCAGGCTGAGGTTTACG
ACACGGTGGTCGTTACCCTGAAGAACATGGCTTCTCATCCCGTTAGTCTTCACGCTGTCGGCGTCTCCTTCTGGAAA
TCTTCCGAAGGCGCTGAATATGAGGATCACACCAGCCAAAGGGAGAAGGAAGACGATAAAGTCCTTCCCGGTAA
AAGCCAAACCTACGTCTGGCAGGTCCTGAAAGAAAATGGTCCAACAGCCTCTGACCCACCATGTCTTACCTACTCA

TACCTGTCTCACGTGGACCTGGTGAAAGACCTGAATTCGGGCCTCATTGGAGCCCTGCTGGTTTGTAGAGAAGGG
AGTCTGACCAGAGAAAGGACCCAGAACCTGCACGAATTTGTACTACTTTTTGCTGTCTTTGATGAAGGGAAAAGTT
GGCACTCAGCAAGAAATGACTCCTGGACACGGGCCATGGATCCCGCACCTGCCAGGGCCCAGCCTGCAATGCACA
CAGTCAATGGCTATGTCAACAGGTCTCTGCCAGGTCTGATCGGATGTCATAAGAAATCAGTCTACTGGCACGTGAT
TGGAATGGGCACCAGCCCGGAAGTGCACTCCATTTTTCTTGAAGGCCACACGTTTCTCGTGAGGCACCATCGCCAG
GCTTCCTTGGAGATCTCGCCACTAACTTTCCTCACTGCTCAGACATTCCTGATGGACCTTGGCCAGTTCCTACTGTTT
TGTCATATCTCTTCCCACCACCATGGTGGCATGGAGGCTCACGTCAGAGTAGAAAGCTGCGCCGAGGAGCCCCAG
CTGCGGAGGAAAGCTGATGAAGAGGAAGATTATGATGACAATTTGTACGACTCGGACATGGACGTGGTCCGGCT
CGATGGTGACGACGTGTCTCCCTTTATCCAAATCCGCTCAGTTGCCAAGAAGCATCCTAAAACTTGGGTACATTAC
ATTGCTGCTGAAGAGGAGGACTGGGACTATGCTCCCTTAGTCCTCGCCCCCGATGACAGAAGTTATAAAAGTCAAT
ATTTGAACAATGGCCCTCAGCGGATTGGTAGGAAGTACAAAAAAGTCCGATTTATGGCATACACAGATGAAACCT
TTAAGACGCGTGAAGCTATTCAGCATGAATCAGGAATCTTGGGACCTTTACTTTATGGGGAAGTTGGAGACACACT
GTTGATTATATTTAAGAATCAAGCAAGCAGACCATATAACATCTACCCTCACGGAATCACTGATGTCCGTCCTTTGT
ATTCAAGGAGATTACCAAAAGGTGTAAAACATTTGAAGGATTTTCCAATTCTGCCAGGAGAAATATTCAAATATAA
ATGGACAGTGACTGTAGAAGATGGGCCAACTAAATCAGATCCGCGGTGCCTGACCCGCTATTACTCTAGTTTCGTT
AATATGGAGAGAGATCTAGCTTCAGGACTCATTGGCCCTCTCCTCATCTGCTACAAAGAATCTGTAGATCAAAGAG
GAAACCAGATAATGTCAGACAAGAGGAATGTCATCCTGTTTTCTGTATTTGATGAGAACCGAAGCTGGTACCTCAC
AGAGAATATACAACGCTTTCTCCCCAATCCAGCTGGAGTGCAGCTTGAGGATCCAGAGTTCCAAGCCTCCAACATC
ATGCACAGCATCAATGGCTATGTTTTTGATAGTTTGCAGTTGTCAGTTTGTTTGCATGAGGTGGCATACTGGTACAT
TCTAAGCATTGGAGCACAGACTGACTTCCTTTCTGTCTTCTTCTCTGGATATACCTTCAAACACAAAATGGTCTATGA
AGACACACTCACCCTATTCCCATTCTCAGGAGAAACTGTCTTCATGTCGATGGAAAACCCAGGTCTATGGATTCTG
GGGTGCCACAACTCAGACTTTCGGAACAGAGGCATGACCGCCTTACTGAAGGTTTCTAGTTGTGACAAGAACACT
GGTGATTATTACGAGGACAGTTATGAAGATATTTCAGCATACTTGCTGAGTAAAAACAATGCCATTGAACCTAGGA
GCTTTGCCCAGAATTCAAGACCCCCTAGTGCGAGCGCTCCAAAGCCTCCGGTCCTGCGACGGCATCAGAGGGACA
TAAGCCTTCCTACTTTTCAGCCGGAGGAAGACAAAATGGACTATGATGATATCTTCTCAACTGAAACGAAGGGAGA
AGATTTTGACATTTACGGTGAGGATGAAAATCAGGACCCTCGCAGCTTTCAGAAGAGAACCCGACACTATTTCATT
GCTGCGGTGGAGCAGCTCTGGGATTACGGGATGAGCGAATCCCCCCGGGCGCTAAGAAACAGGGCTCAGAACGG
AGAGGTGCCTCGGTTCAAGAAGGTGGTCTTCCGGGAATTTGCTGACGGCTCCTTCACGCAGCCGTCGTACCGCGG
GGAACTCAACAAACACTTGGGGCTCTTGGGACCCTACATCAGAGCGGAAGTTGAAGACAACATCATGGTAACTTT
CAAAAACCAGGCGTCTCGTCCCTATTCCTTCTACTCGAGCCTTATTTCTTATCCGGATGATCAGGAGCAAGGGGCA
GAACCTCGACACAACTTCGTCCAGCCAAATGAAACCAGAACTTACTTTTGGAAAGTGCAGCATCACATGGCACCCA
CAGAAGACGAGTTTGACTGCAAAGCCTGGGCCTACTTTTCTGATGTTGACCTGGAAAAAGATGTGCACTCAGGCTT
GATCGGCCCCCTTCTGATCTGCCGCGCCAACACCCTGAACGCTGCTCACGGTAGACAAGTGACCGTGCAAGAATTT
GCTCTGTTTTTCACTATTTTTGATGAGACAAAGAGCTGGTACTTCACTGAAAATGTGGAAAGGAACTGCCGGGCCC
CCTGCCATCTGCAGATGGAGGACCCCACTCTGAAAGAAAACTATCGCTTCCATGCAATCAATGGCTATGTGATGGA
TACACTCCCTGGCTTAGTAATGGCTCAGAATCAAAGGATCCGATGGTATCTGCTCAGCATGGGCAGCAATGAAAAT
ATCCATTCGATTCATTTTAGCGGACACGTGTTCAGTGTACGGAAAAAGGAGGAGTATAAAATGGCCGTGTACAAT
CTCTATCCGGGTGTCTTTGAGACAGTGGAAATGCTACCGTCCAAAGTTGGAATTTGGCGAATAGAATGCCTGATTG
GCGAGCACCTGCAAGCTGGGATGAGCACGACTTTCCTGGTGTACAGCAAGAAGTGTCAGACTCCCCTGGGAATGG
CTTCTGGACACATTAGAGATTTTCAGATTACAGCTTCAGGACAATATGGACAGTGGGCCCCAAAGCTGGCCAGACT
TCATTATTCCGGATCAATCAATGCCTGGAGCACCAAGGAGCCCTTTTCTTGGATCAAGGTGGATCTGTTGGCACCA
ATGATTATTCACGGCATCAAGACCCAGGGTGCCCGTCAGAAGTTCTCCAGCCTCTACATCTCTCAGTTTATCATCAT
GTATAGTCTTGATGGGAAGAAGTGGCAGACTTATCGAGGAAATTCCACTGGAACCTTAATGGTCTTCTTTGGCAAT
GTGGATTCATCTGGGATAAAACACAATATTTTTAACCCTCCAATTATTGCTCGATACATCCGTTTGCACCCAACTCAT
TATAGCATTCGCAGCACTCTTCGCATGGAGTTGATGGGCTGTGATTTAAATAGTTGCAGCATGCCATTGGGAATGG
AGAGTAAAGCAATATCAGATGCACAGATTACTGCTTCATCCTACTTTACCAATATGTTTGCCACCTGGTCTCCTTCA
AAAGCTCGACTTCACCTCCAAGGGAGGAGTAATGCCTGGAGACCTCAGGTGAATAATCCAAAAGAGTGGCTGCAA
GTGGACTTCCAGAAGACAATGAAAGTCACAGGAGTAACTACTCAGGGAGTAAAATCTCTGCTTACCAGCATGTAT

GTGAAGGAGTTCCTCATCTCCAGCAGTCAAGATGGCCATCAGTGGACTCTCTTTTTTCAGAATGGCAAAGTAAAGG
TTTTTCAGGGAAATCAAGACTCCTTCACACCTGTGGTGAACTCTCTAGACCCACCGTTACTGACTCGCTACCTTCGA
ATTCACCCCCAGAGTTGGGTGCACCAGATTGCCCTGAGGATGGAGGTTCTGGGCTGCGAGGCACAGGACCTCTAC
TGA

ET3-MCO
ATGCAGCTGGAGCTCTCAACCTGTGTGTTCCTCTGCCTGCTCCCCCTGGGATTTTCAGCTATCAGGAGATACTATCT
GGGAGCAGTGGAACTGTCCTGGGACTACAGGCAGTCAGAGCTGCTCAGAGAACTGCATGTGGATACTAGGTTCC
CTGCAACAGCTCCTGGAGCACTGCCACTGGGACCTTCAGTGCTGTACAAGAAAACTGTCTTTGTGGAGTTTACAGA
CCAGCTGTTCAGTGTGGCCAGGCCCAGGCCCCCCTGGATGGGGCTGCTGGGACCCACCATCCAGGCTGAAGTGTA
TGATACTGTGGTGGTGACCCTGAAAAACATGGCCTCTCATCCAGTCAGCCTGCATGCTGTGGGAGTGAGCTTCTGG
AAGAGCAGTGAGGGAGCTGAGTATGAAGACCATACCTCACAGAGGGAGAAAGAAGATGATAAGGTGCTGCCAG
GAAAAAGCCAGACCTATGTGTGGCAGGTGCTGAAGGAGAATGGCCCTACAGCTTCAGATCCTCCCTGCCTCACAT
ACTCTTATCTGAGCCATGTGGATCTGGTGAAGGACCTCAATAGTGGCCTGATTGGGGCACTGCTGGTGTGCAGAG
AGGGGTCCCTCACAAGGGAAAGAACTCAGAACCTGCATGAGTTTGTCCTGCTCTTTGCTGTGTTTGATGAGGGAA
AGTCCTGGCACTCAGCAAGGAATGACAGCTGGACCAGGGCTATGGACCCAGCACCAGCCAGAGCTCAGCCAGCT
ATGCACACTGTCAATGGCTATGTGAATAGGTCCCTGCCTGGACTCATTGGCTGCCATAAGAAATCAGTCTATTGGC
ATGTGATTGGAATGGGCACCAGCCCAGAGGTGCATTCCATCTTCCTGGAAGGCCACACATTTCTGGTCAGGCACCA
TAGACAGGCCAGCCTGGAGATCAGCCCACTGACTTTCCTCACAGCACAGACATTTCTGATGGACCTGGGGCAGTTC
CTGCTCTTTTGCCATATCTCAAGTCACCATCATGGAGGGATGGAGGCTCATGTCAGGGTGGAAAGCTGTGCAGAG
GAACCTCAGCTGAGGAGGAAGGCAGATGAGGAAGAGGACTATGATGATAACCTGTATGACTCAGATATGGATGT
GGTGAGGCTGGATGGAGATGATGTCAGCCCATTCATCCAGATCAGGTCAGTGGCTAAGAAACACCCTAAGACCTG
GGTCCACTACATTGCAGCTGAAGAGGAAGATTGGGACTATGCACCCCTGGTGCTGGCCCCAGATGATAGAAGTTA
CAAATCTCAGTATCTGAACAATGGGCCCCAGAGGATTGGAAGGAAGTACAAGAAAGTGAGGTTCATGGCTTATAC
TGATGAGACCTTTAAGACAAGAGAGGCAATCCAGCATGAAAGTGGCATCCTGGGACCACTGCTCTATGGAGAAGT
GGGGGATACCCTGCTCATCATCTTCAAGAACCAGGCCTCAAGGCCTTACAATATCTATCCCCATGGCATCACAGAT
GTGAGGCCTCTCTACAGCAGGAGACTGCCCAAGGGAGTCAAACACCTCAAGGATTTCCCCATCCTGCCAGGGGAA
ATCTTCAAGTATAAATGGACAGTCACTGTGGAAGATGGGCCAACTAAGTCAGATCCTAGGTGCCTGACCAGGTAC
TATTCTAGCTTTGTGAACATGGAGAGGGACCTGGCTTCAGGACTGATTGGACCTCTGCTCATCTGCTACAAAGAAT
CAGTGGACCAGAGGGGCAACCAGATCATGAGTGATAAGAGAAATGTCATCCTGTTCTCAGTGTTTGATGAGAATA
GGAGTTGGTATCTGACAGAAAACATCCAGAGGTTCCTGCCTAATCCTGCAGGAGTGCAGCTGGAGGACCCAGAAT
TTCAGGCTTCAAACATCATGCATAGTATCAATGGCTATGTGTTTGATAGTCTGCAGCTCTCTGTCTGCCTGCATGAG
GTGGCCTACTGGTATATCCTCAGCATTGGAGCTCAGACTGACTTCCTGAGTGTGTTCTTTTCAGGCTACACATTCAA
GCATAAGATGGTCTATGAAGATACCCTGACACTCTTCCCCTTTTCTGGGGAGACTGTGTTTATGAGCATGGAAAAC
CCAGGCCTGTGGATTCTGGGGTGCCACAACAGTGACTTCAGGAATAGAGGGATGACTGCTCTGCTCAAAGTGTCC
TCATGTGATAAGAATACTGGAGATTACTATGAGGACTCTTATGAAGATATCAGTGCATATCTGCTCTCCAAAAACA
ATGCCATTGAGCCCAGGTCATTTGCTCAGAACAGTAGACCACCTTCTGCAAGTGCACCAAAGCCTCCAGTGCTGAG
GAGACACCAGAGGGACATCAGCCTGCCAACCTTCCAGCCTGAGGAAGATAAAATGGACTATGATGATATCTTCTC
CACTGAGACCAAGGGGGAAGATTTTGACATCTATGGAGAGGATGAAAACCAGGACCCCAGGTCCTTCCAGAAGA
GGACCAGACACTACTTTATTGCAGCTGTGGAGCAGCTGTGGGACTATGGCATGTCTGAATCACCTAGAGCTCTGA
GGAACAGAGCACAGAATGGGGAGGTGCCCAGGTTCAAGAAAGTGGTGTTCAGAGAATTTGCAGATGGCTCTTTT
ACCCAGCCTAGCTACAGGGGGGAGCTCAACAAGCATCTGGGGCTGCTGGGACCCTATATCAGAGCAGAGGTGGA
AGATAACATCATGGTGACATTCAAGAATCAGGCCTCAAGACCCTACAGTTTTTATAGTTCTCTGATCAGCTACCCAG
ATGATCAGGAGCAGGGGGCTGAACCAAGGCACAACTTTGTGCAGCCTAATGAGACAAGAACTTACTTTTGGAAGG
TCCAGCATCACATGGCTCCCACAGAGGATGAGTTTGACTGCAAGGCCTGGGCATATTTTTCTGATGTGGACCTGGA
GAAGGATGTGCATAGTGGCCTCATTGGGCCACTGCTCATCTGCAGGGCAAACACACTGAATGCTGCACATGGCAG
GCAGGTCACTGTGCAGGAGTTTGCCCTGTTCTTTACAATCTTTGATGAAACTAAGTCCTGGTACTTCACAGAGAAT
GTGGAAAGGAATTGCAGAGCCCCCTGCCATCTCCAGATGGAGGACCCAACTCTGAAGGAAAACTACAGGTTCCAT

GCTATCAATGGATATGTCATGGATACACTGCCAGGCCTGGTGATGGCACAGAACCAGAGGATCAGGTGGTATCTG
CTCAGCATGGGGTCCAATGAGAATATCCATTCTATCCACTTCTCAGGACATGTCTTTTCAGTGAGGAAGAAAGAGG
AATATAAAATGGCTGTGTACAATCTGTATCCAGGGGTCTTTGAGACAGTGGAAATGCTGCCTAGCAAAGTGGGGA
TCTGGAGAATTGAGTGCCTCATTGGAGAACACCTGCAGGCAGGGATGTCCACCACATTTCTGGTGTACTCAAAGA
AATGCCAGACTCCCCTGGGGATGGCAAGTGGACATATCAGGGACTTCCAGATCACTGCATCAGGACAGTATGGAC
AGTGGGCACCAAAGCTGGCTAGGCTCCACTATAGTGGCTCTATCAATGCTTGGAGTACCAAAGAGCCTTTCTCTTG
GATCAAGGTGGATCTGCTGGCCCCCATGATCATCCATGGAATCAAAACACAGGGAGCTAGACAGAAGTTCAGCTC
CCTGTACATCAGTCAGTTTATCATCATGTATTCTCTGGATGGGAAGAAATGGCAGACCTACAGGGGCAATAGCACT
GGGACACTGATGGTCTTCTTTGGAAATGTGGATTCAAGTGGCATCAAGCACAACATCTTCAATCCTCCCATCATTGC
CAGGTACATCAGACTGCATCCCACACACTATTCAATCAGGAGTACTCTCAGAATGGAGCTGATGGGGTGTGACCTC
AACAGCTGCTCCATGCCACTGGGAATGGAATCCAAGGCAATCTCAGATGCCCAGATCACTGCTTCTAGCTACTTCA
CCAATATGTTTGCAACATGGTCACCCAGTAAAGCAAGGCTGCACCTCCAGGGAAGGTCCAATGCTTGGAGACCCC
AGGTGAACAATCCAAAGGAGTGGCTGCAGGTGGACTTTCAGAAAACCATGAAGGTCACAGGGGTGACTACCCAG
GGAGTGAAAAGTCTGCTCACCTCTATGTATGTCAAGGAGTTCCTGATCTCCTCAAGTCAGGATGGCCACCAGTGGA
CACTGTTCTTTCAGAATGGCAAGGTCAAAGTGTTCCAGGGGAATCAGGACAGCTTTACACCAGTGGTGAACAGCC
TGGACCCCCCTCTGCTCACTAGATATCTGAGAATCCATCCACAGAGCTGGGTGCACCAGATTGCACTCAGAATGGA
GGTCCTGGGCTGTGAAGCCCAGGACCTGTATTGA

<u>HSQ-LCO with CpGs</u>
ATGCAGATCGAACTGTCTACCTGTTTCTTTCTGTGCCTGCTGCGGTTTTGTTTTTCCGCT
ACCAGAAGATACTACCTGGGAGCCGTCGAACTGAGCTGGGATTACATGCAGTCTGACCTG
GGAGAGCTGCCCGTGGACGCTAGATTCCCACCTAGAGTCCCTAAGTCCTTCCCCTTCAAC
ACCAGCGTGGTCTACAAGAAAACCCTGTTCGTGGAGTTTACCGACCACCTGTTCAACATC
GCTAAGCCTAGACCACCATGGATGGGACTGCTGGGACCAACCATCCAGGCCGAGGTGTAC
GACACCGTGGTCATCACCCTGAAAAACATGGCTTCTCACCCCGTGTCCCTGCATGCTGTG
GGCGTCTCCTACTGGAAGGCCAGCGAAGGGGCTGAGTATGACGATCAGACCAGCCAGCGG
GAAAAAGAGGACGATAAGGTGTTCCCTGGCGGGTCCCATACCTACGTGTGGCAGGTCCTG
AAGGAGAATGGACCAATGGCTTCCGACCCTCTGTGCCTGACCTACTCTTATCTGTCCCAC
GTGGACCTGGTCAAGGATCTGAACAGCGGCCTGATCGGGGCTCTGCTGGTGTGTCGCGAA
GGGTCCCTGGCCAAGGAGAAAACCCAGACCCTGCATAAGTTCATCCTGCTGTTCGCCGTG
TTTGACGAAGGAAAAAGCTGGCACTCTGAGACCAAGAACTCTCTGATGCAGGACAGGGAT
GCCGCTTCCGCCAGAGCTTGGCCCAAGATGCACACCGTGAACGGCTACGTCAATAGGAGC
CTGCCTGGACTGATCGGCTGCCACAGAAAGTCCGTGTATTGGCATGTCATCGGAATGGGC
ACCACCCCTGAAGTGCACAGCATCTTCCTGGAGGGGCATACCTTTCTGGTCCGCAACCAC
CGGCAGGCTAGCCTGGAGATCTCTCCAATCACCTTCCTGACCGCCCAGACCCTGCTGATG
GACCTGGGACAGTTCCTGCTGTTTTGCCACATCTCCAGCCACCAGCATGATGGCATGGAG
GCTTACGTGAAAGTCGACTCCTGTCCCGAGGAACCTCAGCTGAGGATGAAGAACAATGAG
GAAGCCGAAGACTATGACGATGACCTGACCGACAGCGAGATGGATGTGGTCCGCTTCGAT
GACGATAACTCTCCCTCCTTTATCCAGATCCGGTCCGTGGCCAAGAAACACCCTAAGACC
TGGGTCCATTACATCGCCGCTGAGGAAGAGGACTGGGATTATGCTCCACTGGTGCTGGCC
CCCGACGATAGATCCTACAAAAGCCAGTATCTGAACAATGGACCCCAGAGGATCGGCAGA
AAGTACAAGAAAGTGAGGTTCATGGCTTATACCGATGAGACCTTTAAGACCAGAGAAGCC
ATCCAGCACGAGTCCGGGATCCTGGGACCTCTGCTGTACGGCGAAGTGGGGGACACCCTG
CTGATCATCTTCAAGAACCAGGCCAGCAGGCCTTACAATATCTATCCACATGGCATCACC
GATGTGAGACCTCTGTACTCCCGCCGGCTGCCAAAGGGCGTGAAACACCTGAAGGACTTC
CCAATCCTGCCCGGGGAAATCTTTAAGTATAAATGGACCGTCACCGTCGAGGATGGGCCC
ACCAAGAGCGACCCTAGGTGCCTGACCAGATACTATTCTTCCTTCGTGAATATGGAGAGA
GACCTGGCTTCCGGACTGATCGGACCCCTGCTGATCTGTTACAAAGAGAGCGTGGATCAG

CGCGGCAACCAGATCATGTCTGACAAGCGGAATGTGATCCTGTTCAGCGTCTTTGACGAA
AACCGCTCTTGGTACCTGACCGAGAACATCCAGCGGTTCCTGCCTAATCCAGCTGGAGTG
CAGCTGGAAGATCCCGAGTTCCAGGCCTCTAACATCATGCATTCCATCAATGGCTACGTG
TTCGACTCCCTGCAGCTGAGCGTGTGCCTGCACGAGGTCGCTTACTGGTATATCCTGAGC
ATCGGAGCCCAGACCGATTTCCTGTCTGTGTTCTTTTCCGGCTACACCTTTAAGCATAAA
ATGGTGTATGAGGACACCCTGACCCTGTTCCCATTTTCCGGCGAAACCGTGTTCATGAGC
ATGGAGAATCCCGGGCTGTGGATCCTGGGATGCCACAACTCCGATTTCAGGAATAGAGGG
ATGACCGCCCTGCTGAAAGTGAGCTCTTGTGACAAGAACACCGGAGACTACTATGAAGAT
AGCTACGAGGACATCTCTGCTTATCTGCTGTCCAAAAACAATGCCATCGAGCCCAGGAGC
TTCTCTCAGAACCCTCCAGTGCTGAAGCGCCACCAGCGGGAGATCACCAGAACCACCCTG
CAGAGCGATCAGGAAGAGATCGACTACGACGATACCATCTCCGTGGAAATGAAGAAAGAG
GACTTCGATATCTATGACGAAGATGAGAACCAGTCTCCCAGGTCCTTCCAGAAGAAAACC
AGACATTACTTTATCGCCGCTGTGGAGCGGCTGTGGGACTATGGCATGTCCAGCTCTCCT
CACGTGCTGAGAAATAGAGCTCAGTCCGGAAGCGTCCCACAGTTCAAGAAAGTGGTCTTC
CAGGAGTTTACCGACGGAAGCTTTACCCAGCCACTGTACCGCGGCGAACTGAACGAGCAC
CTGGGGCTGCTGGGACCCTATATCCGGGCTGAAGTGGAGGATAACATCATGGTCACCTTC
AGGAATCAGGCCAGCAGACCCTACTCTTTTTATTCCAGCCTGATCTCCTACGAAGAGGAC
CAGAGACAGGGAGCTGAACCAAGAAAAAACTTCGTGAAGCCTAATGAGACCAAAACCTAC
TTTTGGAAGGTGCAGCACCATATGGCCCCTACCAAAGACGAGTTCGATTGCAAGGCCTGG
GCTTATTTTAGCGACGTGGATCTGGAGAAGGACGTCCACTCCGGCCTGATCGGGCCACTG
CTGGTGTGTCATACCAACACCCTGAATCCAGCTCACGGAAGGCAGGTGACCGTCCAGGAA
TTCGCCCTGTTCTTTACCATCTTTGATGAGACCAAGAGCTGGTACTTCACCGAAAACATG
GAGAGGAATTGCAGAGCCCCATGTAACATCCAGATGGAAGACCCCACCTTCAAGGAGAAC
TACAGATTTCATGCTATCAATGGGTATATCATGGATACCCTGCCAGGACTGGTCATGGCT
CAGGACCAGAGGATCAGATGGTACCTGCTGAGCATGGGGTCTAACGAGAATATCCACTCC
ATCCATTTCAGCGGACACGTGTTTACCGTCCGCAAGAAAGAAGAGTACAAGATGGCCCTG
TACAACCTGTATCCCGGCGTGTTCGAAACCGTCGAGATGCTGCCTTCCAAGGCTGGGATC
TGGCGGGTGGAATGCCTGATCGGGGAGCACCTGCATGCCGGAATGTCTACCCTGTTCCTG
GTGTACTCCAATAAGTGTCAGACCCCCCTGGGGATGGCTAGCGGACATATCCGCGACTTC
CAGATCACCGCTTCCGGACAGTACGGACAGTGGGCTCCTAAGCTGGCTAGACTGCACTAT
TCTGGCTCCATCAACGCTTGGTCTACCAAAGAGCCTTTCTCCTGGATCAAGGTGGACCTG
CTGGCTCCAATGATCATCCATGGCATCAAAACCCAGGGGGCCAGGCAGAAGTTCTCTTCC
CTGTACATCAGCCAGTTTATCATCATGTATTCTCTGGATGGGAAGAAATGGCAGACCTAC
AGAGGCAATTCCACCGGGACCCTGATGGTGTTCTTTGGCAACGTCGACAGCTCTGGGATC
AAGCACAACATCTTCAATCCCCCTATCATCGCCCGCTACATCCGGCTGCACCCAACCCAT
TATTCCATCCGCAGCACCCTGCGGATGGAGCTGATGGGGTGCGATCTGAACAGCTGTTCT
ATGCCCCTGGGAATGGAGTCTAAGGCCATCTCCGACGCTCAGATCACCGCCTCCAGCTAC
TTCACCAATATGTTTGCTACCTGGTCCCCAAGCAAGGCTAGACTGCATCTGCAGGGAAGA
AGCAACGCTTGGAGACCACAGGTGAACAATCCCAAGGAGTGGCTGCAGGTCGACTTCCAG
AAAACCATGAAGGTGACCGGAGTCACCACCCAGGGCGTGAAAAGCCTGCTGACCTCTATG
TACGTCAAGGAGTTCCTGATCTCTTCCAGCCAGGACGGCACCAGTGGACCCTGTTCTTT
CAGAACGGAAAGGTGAAAGTCTTCCAGGGCAATCAGGATTCCTTTACCCCTGTGGTCAAC
AGCCTGGACCCACCCCTGCTGACCAGGTACCTGAGAATCCACCCACAGTCCTGGGTGCAT
CAGATCGCTCTGAGGATGGAAGTCCTGGGCTGCGAGGCCCAGGACCTGTATTGA

HSQ-LCO CpGs removed

ATGCAGATTGAACTGTCTACCTGTTTCTTTCTGTGCCTGCTGAGGTTTTGTTTTTCTGCT

ACCAGAAGATACTACCTGGGAGCTGTGGAACTGAGCTGGGATTACATGCAGTCTGACCTG
GGAGAGCTGCCTGTGGATGCTAGATTCCCACCTAGAGTCCCTAAGTCCTTCCCCTTCAAC
ACCTCTGTGGTCTACAAGAAAACCCTGTTTGTGGAGTTTACAGACCACCTGTTCAACATT
GCTAAGCCTAGACCACCATGGATGGGACTGCTGGGACCAACCATCCAGGCAGAGGTGTAT
GACACAGTGGTCATCACCCTGAAAAACATGGCTTCTCACCCTGTGTCCCTGCATGCTGTG
GGAGTCTCCTACTGGAAGGCCTCTGAAGGGGCTGAGTATGATGATCAGACCAGCCAGAGG
GAAAAAGAGGATGATAAGGTGTTCCCTGGAGGGTCCCATACCTATGTGTGGCAGGTCCTG
AAGGAGAATGGACCAATGGCTTCTGACCCTCTGTGCCTGACCTACTCTTATCTGTCCCAT
GTGGACCTGGTCAAGGATCTGAACTCTGGCCTGATTGGGGCTCTGCTGGTGTGTAGGGAA
GGGTCCCTGGCCAAGGAGAAAACCCAGACCCTGCATAAGTTCATCCTGCTGTTTGCTGTG
TTTGATGAAGGAAAAAGCTGGCACTCTGAGACCAAGAACTCTCTGATGCAGGACAGGGAT
GCTGCTTCTGCCAGAGCTTGGCCCAAGATGCACACAGTGAATGGCTATGTCAATAGGAGC
CTGCCTGGACTGATTGGCTGCCACAGAAAGTCTGTGTATTGGCATGTCATTGGAATGGGC
ACCACCCCTGAAGTGCACAGCATCTTCCTGGAGGGGCATACCTTTCTGGTCAGGAACCAC
AGGCAGGCTAGCCTGGAGATCTCTCCAATCACCTTCCTGACAGCCCAGACCCTGCTGATG
GACCTGGGACAGTTCCTGCTGTTTTGCCACATCTCCAGCCACCAGCATGATGGCATGGAG
GCTTATGTGAAAGTGGACTCCTGTCCTGAGGAACCTCAGCTGAGGATGAAGAACAATGAG
GAAGCTGAAGACTATGATGATGACCTGACAGACTCTGAGATGGATGTGGTCAGGTTTGAT
GATGATAACTCTCCCTCCTTTATCCAGATCAGGTCTGTGGCCAAGAAACACCCTAAGACC
TGGGTCCATTACATTGCTGCTGAGGAAGAGGACTGGGATTATGCTCCACTGGTGCTGGCC
CCTGATGATAGATCCTACAAAAGCCAGTATCTGAACAATGGACCCCAGAGGATTGGCAGA
AAGTACAAGAAAGTGAGGTTCATGGCTTATACAGATGAGACCTTTAAGACCAGAGAAGCC
ATCCAGCATGAGTCTGGGATCCTGGGACCTCTGCTGTATGGGGAAGTGGGGGACACCCTG
CTGATCATCTTCAAGAACCAGGCCAGCAGGCCTTACAATATCTATCCACATGGCATCACA
GATGTGAGACCTCTGTACTCCAGGAGGCTGCCAAAGGGGGTGAAACACCTGAAGGACTTC
CCAATCCTGCCTGGGGAAATCTTTAAGTATAAATGGACAGTCACAGTGGAGGATGGGCCC
ACCAAGTCTGACCCTAGGTGCCTGACCAGATACTATTCTTCCTTTGTGAATATGGAGAGA
GACCTGGCTTCTGGACTGATTGGACCCCTGCTGATCTGTTACAAAGAGTCTGTGGATCAG
AGGGGCAACCAGATCATGTCTGACAAGAGGAATGTGATCCTGTTCTCTGTCTTTGATGAA
AACAGGTCTTGGTACCTGACAGAGAACATCCAGAGGTTCCTGCCTAATCCAGCTGGAGTG
CAGCTGGAAGATCCTGAGTTCCAGGCCTCTAACATCATGCATTCCATCAATGGCTATGTG
TTTGACTCCCTGCAGCTGTCTGTGTGCCTGCATGAGGTGGCTTACTGGTATATCCTGAGC
ATTGGAGCCCAGACAGATTTCCTGTCTGTGTTCTTTTCTGGCTACACCTTTAAGCATAAA
ATGGTGTATGAGGACACCCTGACCCTGTTCCCATTTTCTGGAGAAACTGTGTTCATGAGC
ATGGAGAATCCTGGGCTGTGGATCCTGGGATGCCACAACTCTGATTTCAGGAATAGAGGG
ATGACAGCCCTGCTGAAAGTGAGCTCTTGTGACAAGAACACAGGAGACTACTATGAAGAT
AGCTATGAGGACATCTCTGCTTATCTGCTGTCCAAAAACAATGCCATTGAGCCCAGGAGC
TTCTCTCAGAACCCTCCAGTGCTGAAGAGGCACCAGAGGGAGATCACCAGAACCACCCTG
CAGTCTGATCAGGAAGAGATTGACTATGATGATACCATCTCTGTGGAAATGAAGAAAGAG
GACTTTGATATCTATGATGAAGATGAGAACCAGTCTCCCAGGTCCTTCCAGAAGAAAACC
AGACATTACTTTATTGCTGCTGTGGAGAGGCTGTGGGACTATGGCATGTCCAGCTCTCCT
CATGTGCTGAGAAATAGAGCTCAGTCTGGATCTGTCCCACAGTTCAAGAAAGTGGTCTTC
CAGGAGTTTACAGATGGAAGCTTTACCCAGCCACTGTACAGGGGAGAACTGAATGAGCAC
CTGGGGCTGCTGGGACCCTATATCAGGGCTGAAGTGGAGGATAACATCATGGTCACCTTC
AGGAATCAGGCCAGCAGACCCTACTCTTTTTATTCCAGCCTGATCTCCTATGAAGAGGAC
CAGAGACAGGGAGCTGAACCAAGAAAAAACTTTGTGAAGCCTAATGAGACCAAAACCTAC
TTTTGGAAGGTGCAGCACCATATGGCCCCTACCAAAGATGAGTTTGATTGCAAGGCCTGG
GCTTATTTTTCTGATGTGGATCTGGAGAAGGATGTCCACTCTGGCCTGATTGGGCCACTG

CTGGTGTGTCATACCAACACCCTGAATCCAGCTCATGGAAGGCAGGTGACAGTCCAGGAA
TTTGCCCTGTTCTTTACCATCTTTGATGAGACCAAGAGCTGGTACTTCACAGAAAACATG
GAGAGGAATTGCAGAGCCCCATGTAACATCCAGATGGAAGACCCCACCTTCAAGGAGAAC
TACAGATTTCATGCTATCAATGGGTATATCATGGATACCCTGCCAGGACTGGTCATGGCT
CAGGACCAGAGGATCAGATGGTACCTGCTGAGCATGGGGTCTAATGAGAATATCCACTCC
ATCCATTTCTCTGGACATGTGTTTACAGTAAGGAAGAAAGAAGAGTACAAGATGGCCCTG
TACAACCTGTATCCTGGGGTGTTTGAAACAGTGGAGATGCTGCCTTCCAAGGCTGGGATC
TGGAGGGTGGAATGCCTGATTGGGGAGCACCTGCATGCTGGAATGTCTACCCTGTTCCTG
GTGTACTCCAATAAGTGTCAGACCCCCCTGGGGATGGCTTCTGGACATATCAGGGACTTC
CAGATCACAGCTTCTGGACAGTATGGACAGTGGGCTCCTAAGCTGGCTAGACTGCACTAT
TCTGGCTCCATCAATGCTTGGTCTACCAAAGAGCCTTTCTCCTGGATCAAGGTGGACCTG
CTGGCTCCAATGATCATCCATGGCATCAAAACCCAGGGGGCCAGGCAGAAGTTCTCTTCC
CTGTACATCAGCCAGTTTATCATCATGTATTCTCTGGATGGGAAGAAATGGCAGACCTAC
AGAGGCAATTCCACAGGGACCCTGATGGTGTTCTTTGGCAATGTGGACAGCTCTGGGATC
AAGCACAACATCTTCAATCCCCCTATCATTGCCAGGTACATCAGACTGCACCCAACCCAT
TATTCCATCAGGAGCACCCTGAGAATGGAGCTGATGGGGTGTGATCTGAACAGCTGTTCT
ATGCCCCTGGGAATGGAGTCTAAGGCCATCTCTGATGCTCAGATCACAGCCTCCAGCTAC
TTCACCAATATGTTTGCTACCTGGTCCCCAAGCAAGGCTAGACTGCATCTGCAGGGAAGA
AGCAATGCTTGGAGACCACAGGTGAACAATCCCAAGGAGTGGCTGCAGGTGGACTTCCAG
AAAACCATGAAGGTGACAGGAGTCACCACCCAGGGAGTGAAAAGCCTGCTGACCTCTATG
TATGTCAAGGAGTTCCTGATCTCTTCCAGCCAGGATGGGCACCAGTGGACCCTGTTCTTT
CAGAATGGAAAGGTGAAAGTCTTCCAGGGCAATCAGGATTCCTTTACCCCTGTGGTCAAC
AGCCTGGACCCACCCCTGCTGACCAGGTACCTGAGAATCCACCCACAGTCCTGGGTGCAT
CAGATTGCTCTGAGGATGGAAGTCCTGGGCTGTGAGGCCCAGGACCTGTATTGA

<u>HSQ-NoCo</u>
ATGCAAATAGAGCTCTCCACCTGCTTCTTTCTGTGCCTTTTGCGATTCTGCTTTAGTGCCACCAGAAGATACTACCTG
GGTGCAGTGGAACTGTCATGGGACTATATGCAAAGTGATCTCGGTGAGCTGCCTGTGGACGCAAGATTTCCTCCT
AGAGTGCCAAAATCTTTTCCATTCAACACCTCAGTCGTGTACAAAAAGACTCTGTTTGTAGAATTCACGGACCACCT
TTTCAACATCGCTAAGCCAAGGCCACCCTGGATGGGTCTGCTAGGTCCTACCATCCAGGCTGAGGTTTATGATACA
GTGGTCATTACACTTAAGAACATGGCTTCCCATCCTGTCAGTCTTCATGCTGTTGGTGTATCCTACTGGAAAGCTTC
TGAGGGAGCTGAATATGATGATCAGACCAGTCAAAGGGAGAAAGAAGATGATAAAGTCTTCCCTGGTGGAAGCC
ATACATATGTCTGGCAGGTCCTGAAAGAGAATGGTCCAATGGCCTCTGACCCACTGTGCCTTACCTACTCATATCTT
TCTCATGTGGACCTGGTAAAAGACTTGAATTCAGGCCTCATTGGAGCCCTACTAGTATGTAGAGAAGGGAGTCTG
GCCAAGGAAAAGACACAGACCTTGCACAAATTTATACTACTTTTTGCTGTATTTGATGAAGGGAAAAGTTGGCACT
CAGAAACAAAGAACTCCTTGATGCAGGATAGGGATGCTGCATCTGCTCGGGCCTGGCCTAAAATGCACACAGTCA
ATGGTTATGTAAACAGGTCTCTGCCAGGTCTGATTGGATGCCACAGGAAATCAGTCTATTGGCATGTGATTGGAAT
GGGCACCACTCCTGAAGTGCACTCAATATTCCTCGAAGGTCACACATTTCTTGTGAGGAACCATCGCCAGGCGTCC
TTGGAAATCTCGCCAATAACTTTCCTTACTGCTCAAACACTCTTGATGGACCTTGGACAGTTTCTACTGTTTTGTCAT
ATCTCTTCCCACCAACATGATGGCATGGAAGCTTATGTCAAAGTAGACAGCTGTCCAGAGGAACCCCAACTACGAA
TGAAAAATAATGAAGAAGCGGAAGACTATGATGATGATCTTACTGATTCTGAAATGGATGTGGTCAGGTTTGATG
ATGACAACTCTCCTTCCTTTATCCAAATTCGCTCAGTTGCCAAGAAGCATCCTAAAACTTGGGTACATTACATTGCT
GCTGAAGAGGAGGACTGGGACTATGCTCCCTTAGTCCTCGCCCCCGATGACAGAAGTTATAAAAGTCAATATTTG
AACAATGGCCCTCAGCGGATTGGTAGGAAGTACAAAAAAGTCCGATTTATGGCATACACAGATGAAACCTTTAAG
ACGCGTGAAGCTATTCAGCATGAATCAGGAATCTTGGGACCTTTACTTTATGGGGAAGTTGGAGACACACTGTTG
ATTATATTTAAGAATCAAGCAAGCAGACCATATAACATCTACCCTCACGGAATCACTGATGTCCGTCCTTTGTATTC
AAGGAGATTACCAAAAGGTGTAAAACATTTGAAGGATTTTCCAATTCTGCCAGGAGAAATATTCAAATATAAATGG
ACAGTGACTGTAGAAGATGGGCCAACTAAATCAGATCCGCGGTGCCTGACCCGCTATTACTCTAGTTTCGTTAATA

TGGAGAGAGATCTAGCTTCAGGACTCATTGGCCCTCTCCTCATCTGCTACAAAGAATCTGTAGATCAAAGAGGAAA
CCAGATAATGTCAGACAAGAGGAATGTCATCCTGTTTTCTGTATTTGATGAGAACCGAAGCTGGTACCTCACAGAG
AATATACAACGCTTTCTCCCCAATCCAGCTGGAGTGCAGCTTGAGGATCCAGAGTTCCAAGCCTCCAACATCATGC
ACAGCATCAATGGCTATGTTTTTGATAGTTTGCAGTTGTCAGTTTGTTTGCATGAGGTGGCATACTGGTACATTCTA
AGCATTGGAGCACAGACTGACTTCCTTTCTGTCTTCTTCTCTGGATATACCTTCAAACACAAAATGGTCTATGAAGA
CACACTCACCCTATTCCCATTCTCAGGAGAAACTGTCTTCATGTCGATGGAAAACCCAGGTCTATGGATTCTGGGGT
GCCACAACTCAGACTTTCGGAACAGAGGCATGACCGCCTTACTGAAGGTTTCTAGTTGTGACAAGAACACTGGTG
ATTATTACGAGGACAGTTATGAAGATATTTCAGCATACTTGCTGAGTAAAAACAATGCCATTGAACCTAGGAGCTT
CTCTCAGAATCCACCAGTCTTGAAACGCCATCAACGGGAAATAACTCGTACTACTCTTCAGTCAGATCAAGAGGAA
ATTGACTATGATGATACCATATCAGTTGAAATGAAGAAGGAAGATTTTGACATTTATGATGAGGATGAAAATCAG
AGCCCCCGCAGCTTTCAAAAGAAAACACGACACTATTTTATTGCTGCAGTGGAGAGGCTCTGGGATTATGGGATG
AGTAGCTCCCCACATGTTCTAAGAAACAGGGCTCAGAGTGGCAGTGTCCCTCAGTTCAAGAAAGTTGTTTTCCAGG
AATTTACTGATGGCTCCTTTACTCAGCCCTTATACCGTGGAGAACTAAATGAACATTTGGGACTCCTGGGGCCATAT
ATAAGAGCAGAAGTTGAAGATAATATCATGGTAACTTTCAGAAATCAGGCCTCTCGTCCCTATTCCTTCTATTCTAG
CCTTATTTCTTATGAGGAAGATCAGAGGCAAGGAGCAGAACCTAGAAAAAACTTTGTCAAGCCTAATGAAACCAA
AACTTACTTTTGGAAAGTGCAACATCATATGGCACCCACTAAAGATGAGTTTGACTGCAAAGCCTGGGCTTATTTCT
CTGATGTTGACCTGGAAAAAGATGTGCACTCAGGCCTGATTGGACCCCTTCTGGTCTGCCACACTAACACACTGAA
CCCTGCTCATGGGAGACAAGTGACAGTACAGGAATTTGCTCTGTTTTTCACCATCTTTGATGAGACCAAAAGCTGG
TACTTCACTGAAAATATGGAAAGAAACTGCAGGGCTCCCTGCAATATCCAGATGGAAGATCCCACTTTTAAAGAGA
ATTATCGCTTCCATGCAATCAATGGCTACATAATGGATACACTACCTGGCTTAGTAATGGCTCAGGATCAAAGGAT
TCGATGGTATCTGCTCAGCATGGGCAGCAATGAAAACATCCATTCTATTCATTTCAGTGGACATGTGTTCACTGTAC
GAAAAAAAGAGGAGTATAAAATGGCACTGTACAATCTCTATCCAGGTGTTTTTGAGACAGTGGAAATGTTACCATC
CAAAGCTGGAATTTGGCGGGTGGAATGCCTTATTGGCGAGCATCTACATGCTGGGATGAGCACACTTTTTCTGGT
GTACAGCAATAAGTGTCAGACTCCCCTGGGAATGGCTTCTGGACACATTAGAGATTTTCAGATTACAGCTTCAGGA
CAATATGGACAGTGGGCCCCAAAGCTGGCCAGACTTCATTATTCCGGATCAATCAATGCCTGGAGCACCAAGGAG
CCCTTTTCTTGGATCAAGGTGGATCTGTTGGCACCAATGATTATTCACGGCATCAAGACCCAGGGTGCCCGTCAGA
AGTTCTCCAGCCTCTACATCTCTCAGTTTATCATCATGTATAGTCTTGATGGGAAGAAGTGGCAGACTTATCGAGGA
AATTCCACTGGAACCTTAATGGTCTTCTTTGGCAATGTGGATTCATCTGGGATAAAACACAATATTTTTAACCCTCC
AATTATTGCTCGATACATCCGTTTGCACCCAACTCATTATAGCATTCGCAGCACTCTTCGCATGGAGTTGATGGGCT
GTGATTTAAATAGTTGCAGCATGCCATTGGGAATGGAGAGTAAAGCAATATCAGATGCACAGATTACTGCTTCATC
CTACTTTACCAATATGTTTGCCACCTGGTCTCCTTCAAAAGCTCGACTTCACCTCCAAGGGAGGAGTAATGCCTGGA
GACCTCAGGTGAATAATCCAAAAGAGTGGCTGCAAGTGGACTTCCAGAAGACAATGAAAGTCACAGGAGTAACT
ACTCAGGGAGTAAAATCTCTGCTTACCAGCATGTATGTGAAGGAGTTCCTCATCTCCAGCAGTCAAGATGGCCATC
AGTGGACTCTCTTTTTTCAGAATGGCAAAGTAAAGGTTTTTCAGGGAAATCAAGACTCCTTCACACCTGTGGTGAA
CTCTCTAGACCCACCGTTACTGACTCGCTACCTTCGAATTCACCCCCAGAGTTGGGTGCACCAGATTGCCCTGAGGA
TGGAGGTTCTGGGCTGCGAGGCACAGGACCTCTACTGA

HSQ-MCO
ATGCAGATTGAGCTCAGCACCTGCTTCTTTCTGTGCCTGCTCAGGTTCTGCTTTTCAGCCACAAGGAGATACTATCT
GGGAGCTGTGGAACTGTCATGGGATTACATGCAGAGTGACCTGGGAGAGCTCCCTGTGGATGCTAGGTTCCCCCC
AAGGGTCCCAAAGTCTTTCCCTTTTAATACCAGTGTGGTCTATAAGAAAACACTCTTTGTGGAATTTACTGATCACC
TGTTCAACATTGCAAAGCCAAGGCCTCCCTGGATGGGACTGCTGGGACCTACCATCCAGGCTGAGGTGTATGACA
CTGTGGTCATCACACTGAAAAACATGGCATCTCACCCTGTCAGCCTGCATGCAGTGGGAGTCAGCTACTGGAAGG
CTTCAGAAGGGGCAGAGTATGATGATCAGACAAGCCAGAGAGAAAAAGAGGATGATAAGGTGTTCCCAGGAGG
GAGCCATACTTATGTGTGGCAGGTCCTGAAGGAGAATGGCCCAATGGCCAGTGACCCACTGTGCCTCACCTACTC
ATATCTGAGTCATGTGGACCTGGTCAAGGATCTCAACTCAGGCCTGATTGGGGCACTGCTGGTGTGCAGGGAAGG
CTCACTGGCCAAGGAGAAAACCCAGACACTGCATAAGTTCATCCTGCTCTTTGCTGTGTTTGATGAAGGGAAATCT
TGGCACAGTGAGACCAAGAACAGTCTGATGCAGGACAGGGATGCTGCTTCTGCCAGAGCTTGGCCCAAGATGCA

CACAGTGAATGGATATGTCAATAGGTCCCTGCCAGGACTCATTGGCTGCCACAGAAAGTCAGTGTATTGGCATGTC
ATTGGAATGGGCACCACACCAGAAGTGCACAGCATCTTCCTGGAGGGGCATACCTTTCTGGTCAGGAACCACAGG
CAGGCCAGCCTGGAGATCAGCCCAATCACCTTCCTGACAGCCCAGACTCTGCTCATGGATCTGGGGCAGTTCCTGC
TCTTTTGCCACATCAGCTCCCACCAGCATGATGGAATGGAGGCATATGTGAAAGTGGACTCCTGCCCAGAGGAAC
CACAGCTGAGGATGAAGAACAATGAGGAAGCTGAAGACTATGATGATGACCTGACAGACTCAGAGATGGATGTG
GTCAGGTTTGATGATGATAACAGCCCCTCCTTTATCCAGATCAGAAGTGTGGCCAAGAAACACCCAAAGACATGG
GTCCATTACATTGCAGCTGAGGAAGAGGACTGGGATTATGCACCTCTGGTGCTGGCCCCAGATGATAGATCCTAC
AAATCACAGTATCTGAACAATGGACCCCAGAGGATTGGCAGAAAGTACAAGAAAGTGAGGTTCATGGCCTATACT
GATGAAACATTTAAGACTAGAGAAGCTATCCAGCATGAGTCAGGCATCCTGGGACCACTGCTCTATGGAGAAGTG
GGGGACACCCTGCTCATCATCTTCAAGAACCAGGCTTCCAGGCCATACAATATCTATCCTCATGGCATCACAGATG
TGAGACCACTCTACTCAAGGAGACTGCCTAAGGGAGTCAAACACCTCAAGGACTTCCCTATCCTGCCAGGGGAAA
TCTTTAAGTATAAATGGACTGTGACAGTGGAGGATGGGCCCACTAAGAGTGACCCAAGGTGCCTGACCAGATACT
ATTCAAGTTTTGTGAATATGGAAAGGGATCTGGCATCAGGACTGATTGGACCTCTGCTCATCTGCTACAAAGAGAG
TGTGGATCAGAGGGGCAACCAGATCATGTCAGACAAGAGGAATGTGATCCTGTTCAGTGTCTTTGATGAAAACAG
GTCTTGGTATCTGACAGAGAACATCCAGAGATTCCTGCCAAATCCTGCAGGGGTGCAGCTGGAAGATCCAGAGTT
TCAGGCCTCAAACATCATGCATAGTATCAATGGATATGTGTTTGACAGTCTGCAGCTCTCTGTGTGCCTGCATGAA
GTGGCCTACTGGTATATCCTGTCCATTGGAGCTCAGACAGATTTCCTGAGTGTGTTCTTTTCAGGCTACACTTTTAA
GCATAAAATGGTCTATGAGGACACACTGACTCTCTTCCCTTTTAGTGGGGAAACAGTGTTTATGAGCATGGAGAAT
CCAGGGCTGTGGATTCTGGGATGCCACAACAGTGATTTCAGGAATAGAGGCATGACTGCTCTGCTCAAAGTGTCT
AGCTGTGACAAGAACACAGGGGACTACTATGAAGATTCTTATGAGGACATCAGTGCTTATCTGCTCTCCAAAAACA
ATGCAATTGAACCCAGATCATTCAGTCAGAATCCACCTGTGCTGAAGAGGCACCAGAGAGAGATCACTAGGACTA
CCCTGCAGTCAGATCAGGAAGAGATTGACTATGATGATACCATCTCAGTGGAAATGAAGAAAGAGGACTTTGATA
TCTATGATGAAGATGAGAACCAGAGTCCAAGGTCTTTCCAGAAGAAAACCAGACATTACTTTATTGCTGCAGTGGA
GAGGCTGTGGGATTATGGAATGTCCTCAAGTCCACATGTGCTGAGGAATAGGGCACAGTCTGGCAGTGTCCCTCA
GTTCAAGAAAGTGGTCTTCCAGGAGTTTACAGATGGCAGCTTCACTCAGCCTCTGTACAGGGGAGAACTCAATGA
GCACCTGGGGCTGCTGGGACCCTATATCAGAGCTGAAGTGGAGGATAACATCATGGTCACCTTCAGGAATCAGGC
TTCAAGACCCTACAGTTTTTATTCTAGCCTGATCAGCTATGAAGAGGACCAGAGGCAGGGAGCTGAACCTAGGAA
AAACTTTGTGAAGCCAAATGAGACCAAAACATACTTTTGGAAGGTCCAGCACCACATGGCACCAACCAAAGATGA
GTTTGATTGCAAGGCATGGGCCTATTTTTCAGATGTGGATCTGGAGAAGGATGTCCACAGTGGCCTCATTGGGCCT
CTGCTGGTGTGCCATACTAACACCCTGAATCCAGCTCATGGCAGGCAGGTGACAGTCCAGGAGTTTGCACTGTTCT
TTACCATCTTTGATGAGACAAAGTCCTGGTACTTCACTGAAAACATGGAGAGGAATTGCAGAGCTCCTTGCAACAT
CCAGATGGAAGACCCCACCTTCAAGGAGAACTACAGATTTCATGCAATCAATGGGTATATCATGGATACACTGCCA
GGACTGGTGATGGCCCAGGACCAGAGGATCAGATGGTATCTGCTCAGCATGGGGTCCAATGAGAATATCCACTCT
ATCCATTTCAGTGGACATGTGTTTACAGTCAGAAAGAAAGAAGAGTATAAAATGGCCCTGTACAACCTCTATCCAG
GAGTGTTTGAAACAGTGGAGATGCTGCCAAGCAAGGCTGGGATCTGGAGGGTGGAATGCCTCATTGGGGAGCAC
CTGCATGCAGGAATGTCAACCCTGTTTCTGGTCTACAGTAATAAGTGCCAGACACCTCTGGGAATGGCAAGTGGA
CATATCAGGGATTTCCAGATCACTGCTAGTGGACAGTATGGACAGTGGGCACCAAAGCTGGCTAGACTCCACTATT
CAGGCTCAATCAATGCTTGGTCCACCAAAGAGCCATTCTCATGGATCAAGGTGGACCTGCTGGCTCCTATGATCAT
CCATGGCATCAAAACACAGGGGGCAAGGCAGAAGTTCTCCTCACTGTACATCTCTCAGTTTATCATCATGTATAGC
CTGGATGGCAAGAAATGGCAGACCTACAGGGGCAATAGCACAGGGACTCTGATGGTGTTCTTTGGCAATGTGGA
CAGCAGTGGGATCAAGCACAACATCTTCAATCCCCCAATCATTGCAAGGTACATCAGACTGCACCCCACCCATTATT
CAATCAGGAGTACACTCAGGATGGAACTGATGGGGTGTGATCTCAACAGTTGCTCTATGCCACTGGGAATGGAGT
CCAAGGCAATCTCAGATGCCCAGATCACTGCTAGCTCCTACTTCACTAATATGTTTGCTACCTGGAGCCCCTCCAAA
GCAAGGCTGCACCTCCAGGGAAGGAGCAATGCATGGAGGCCTCAGGTGAACAATCCCAAGGAATGGCTGCAGGT
GGATTTCCAGAAAACTATGAAGGTGACTGGAGTCACAACTCAGGGAGTGAAAAGTCTGCTCACTTCTATGTATGTC
AAGGAGTTCCTGATCTCAAGTTCTCAGGATGGCCACCAGTGGACCCTGTTCTTTCAGAATGGAAAGGTGAAAGTCT
TCCAGGGCAATCAGGATTCCTTTACACCAGTGGTCAACTCACTGGACCCTCCCCTGCTCACTAGATATCTGAGAATC

CACCCTCAGAGCTGGGTGCATCAGATTGCTCTCAGAATGGAAGTCCTGGGCTGTGAGGCACAGGACCTGTATTGA
G

## FIX sequences

FIX-LCO
ATGCAGAGGGTGAACATGATCATGGCTGAGTCTCCTGGACTGATCACCATCTGCCTGCTGGGCTATCTGCTGTCTG
CTGAGTGTACAGTGTTCCTGGACCATGAAAATGCTAATAAAATCCTGAACAGGCCAAAGAGGTACAATTCTGGGA
AACTGGAGGAATTTGTGCAGGGAAACCTGGAGAGGGAATGCATGGAGGAAAAGTGTAGCTTTGAGGAAGCCAG
GGAGGTGTTTGAAAATACAGAGAGGACCACAGAGTTCTGGAAACAGTATGTGGATGGGGATCAGTGTGAGTCCA
ACCCCTGTCTGAATGGAGGGTCTTGCAAGGATGATATCAACTCCTATGAGTGCTGGTGTCCTTTTGGATTTGAAGG
CAAGAATTGTGAGCTGGATGTGACCTGTAACATCAAAAATGGGAGGTGTGAGCAGTTCTGTAAGAACTCTGCTGA
TAATAAAGTGGTCTGCAGCTGTACAGAAGGCTACAGGCTGGCTGAGAACCAGAAGAGCTGTGAACCAGCTGTGC
CCTTCCCTTGTGGGAGGGTGTCTGTCAGCCAGACCAGCAAGCTGACCAGAGCTGAGACAGTGTTTCCTGATGTGG
ATTATGTCAACTCTACAGAGGCTGAAACCATCCTGGACAACATCACCCAGTCTACCCAGTCCTTCAATGACTTTACC
AGGGTGGTGGGAGGGGAGGATGCTAAGCCAGGACAGTTCCCCTGGCAGGTGGTCCTGAATGGCAAAGTGGATG
CTTTTTGTGGGGGCTCCATTGTGAATGAGAAGTGGATTGTCACAGCTGCTCACTGTGTGGAAACTGGGGTCAAGA
TCACAGTGGTGGCTGGAGAGCACAACATTGAGGAAACTGAACATACAGAGCAGAAAAGGAATGTGATCAGAATC
ATCCCCCACCATAACTACAATGCTGCTATCAACAAGTATAATCATGACATTGCCCTGCTGGAACTGGATGAGCCTCT
GGTGCTGAACAGCTATGTCACCCCAATCTGCATTGCTGACAAGGAGTATACCAATATCTTCCTGAAATTTGGGTCT
GGATATGTGTCTGGGTGGGGAAGGGTCTTCCACAAGGGAAGGTCTGCTCTGGTGCTGCAGTATCTGAGGGTGCC
CCTGGTGGACAGAGCTACCTGCCTGCTGAGCACCAAGTTCACCATCTACAACAATATGTTCTGTGCTGGATTTCAT
GAGGGAGGGAGGGACTCCTGTCAGGGAGATTCTGGAGGCCCTCATGTGACAGAGGTGGAAGGCACCAGCTTCCT
GACTGGCATCATCTCTTGGGGGGAGGAATGTGCTATGAAGGGGAAATATGGAATCTATACCAAGGTGTCCAGATA
TGTCAACTGGATCAAGGAGAAAACCAAGCTGACCTGA

FIX-HCO
ATGCAGAGGGTGAATATGATTATGGCTGAGTCCCCTGGGCTGATTACCATTTGCCTGCTGGGATACCTGCTGTCTG
CTGAGTGTACAGTGTTCCTGGACCATGAGAATGCAAATAAGATCCTGAACAGGCCCAAAAGATATAATAGTGGAA
AGCTGGAGGAATTTGTGCAGGGCAACCTGGAGAGAGAATGCATGGAGGAAAAGTGTAGCTTTGAGGAAGCCAG
GGAGGTGTTTGAAAATACAGAGAGAACCACAGAATTCTGGAAGCAGTATGTGGATGGAGATCAGTGTGAGAGCA
ACCCCTGTCTGAATGGAGGGAGTTGCAAAGATGATATCAACTCATATGAATGCTGGTGTCCTTTTGGATTTGAAGG
CAAAAATTGTGAGCTGGATGTGACCTGTAACATTAAGAATGGGAGGTGTGAGCAGTTTTGTAAAAACTCTGCTGA
TAATAAGGTGGTCTGCAGTTGTACAGAAGGGTATAGACTGGCTGAGAACCAGAAGTCCTGTGAACCAGCTGTGCC
CTTCCCTTGTGGAAGGGTGTCTGTCTCCCAGACTTCAAAACTGACCAGAGCTGAGACTGTGTTTCCTGATGTGGAT
TATGTCAACAGCACAGAGGCTGAAACTATCCTGGACAACATTACTCAGTCTACCCAGAGTTTCAATGACTTTACCA
GAGTGGTGGGAGGAGAGGATGCTAAACCAGGCCAGTTCCCCTGGCAGGTGGTCCTGAATGGGAAGGTGGATGC
ATTTTGTGGGGGATCTATTGTGAATGAGAAATGGATTGTCACAGCTGCTCACTGTGTGGAAACTGGGGTCAAGAT
CACAGTGGTGGCTGGAGAGCACAACATTGAGGAAACAGAACATACTGAGCAGAAGAGGAATGTGATCAGAATCA
TTCCTCACCATAACTACAATGCAGCCATCAACAAATATAATCATGACATTGCCCTGCTGGAACTGGATGAGCCTCTG
GTGCTGAACAGCTATGTCACACCAATCTGCATTGCTGACAAGGAGTACACTAACATCTTCCTGAAGTTTGGGTCAG
GATATGTGTCTGGATGGGGAAGAGTCTTCCACAAGGGCAGGTCTGCACTGGTGCTGCAGTATCTGAGAGTGCCTC
TGGTGGATAGGGCCACTTGTCTGCTGTCTACCAAGTTCACCATCTACAACAATATGTTCTGTGCTGGATTTCATGAG
GGAGGGAGAGACTCCTGTCAGGGAGATTCTGGAGGCCCACATGTGACAGAGGTGGAAGGCACCAGCTTCCTGAC
AGGCATCATTTCCTGGGGGGAGGAATGTGCAATGAAGGGGAAATATGGAATCTACACCAAAGTGAGCAGGTATG
TGAACTGGATCAAGGAAAAGACCAAACTGACATGA

## 6x tRNA sequence

6x-tRNA
GTCGACTACGTAGAATCCGGAGCCGTCTTTGTCTTCCAGCTCCATCTTTTCCACCTTTTGCTTAGGCAGTCCCCCGA
GTCGTGTCAAGGCTGAGGAGTAGAAATGGAACAGCACTAATATTAATGGCAAAACCGTTGTGAAATAGGGTTACT
TTCTGTTTAAGCAAGGAAAATAAAGTAAAGCAATGGGAAAAAAATTAAAAGCAAAAGGAATGGAGGTGCCGGGG
ATTGAACCCGGGGCCTCGTGCATGCTAAGCACGCGCTCTACCACTGAGCTACACCCCCGTACTGAAACGGTTCTCT
CGAGAGTATATTCAAGATCAGAATCTGACCCTTTTGCTAGGTTTCAGAACCATTAGTTGTAATCAGCCAAGGTCTAT
TTTATTTAGTTATTTCTGATATCTCAAATTTAGGTTTTGCGTCCCTCTTTGCTGACAGCTGAGCAAACCGCATTCTAC
ACCGAAGGCCCTCTATTGATGGCCCTGATTTAAATCCCTTCCGCCGCCTGCCGCAGGTGGCTAGGGTCTGAGCACA
CTTGAACCTCACACCCGCCCCAGGGGTAGCTCCTTGGTTCCCTTCAGCCCCGATGTGTCCCTCGTGCTTTGAAATGG
AATTACAGTTTTGGTTAAAAACATGCCTTTTCCGAGTTAGGAAGAATCTAAATCGACTGAACGCCAGTCTAAAATTT
CGGCGTTCCCACACCGGGAGTCGAACCCGGGCCGCCTGGGTGAAAACCAGGAATCCTAACCGCTAGACCATGTG
GGAGACGGCAATAGCGACTCCAAGCCTAGACAAATTGAGTCTTCTCGGTCGGCTTCCGCCCACTCCATCGCGTTCA
TCCGTAGGCGTCAAACCTGCTCCTGCGCCTGCGCGGAGTCTGCAGCGGTTTAAACCGTTCAGGTTCGCATTCTACT
GTTTCTCTCCTTGCAGGGGCCCTTGAATCTTTCTTCAATCTACTCTCGCGTGCCCGGGCGACTGGGCATAACCCTAC
AGGTTCATGTGGGGTGGGTGGCGCGCGCTAGCGGTGAAGGTCACTCACAATTGCGCGCTGGGCAGACGACGGCA
GCCATTACTTTTACCTCGATCGCTGTTTTCCTGGATCCGCACGGGTCCAACCCGACTCATCCCAACCAACCTGAGGT
ATGAAAACCAGGAAAGAGAGCTAGCACCGGAGCGTTGGTGGTATAGTGGTAAGCATAGCTGCCTTCCAAGCAGT
TGACCCGGGTTCGATTCCCGGCCAACGCAAGTCGTTTTGGGTGTTTTTTCCCCCCCCCGCCTTTTCCTTTTCGTGTTT
TCTGGGCCCCAGCATCGTTGAGGGTTTTCGTGAGGTTTTCCTGAGGAAACTTCCGCTCCGAAAGGACCCACTTTCC
GCTACACCCGCGACCACGGCTGGACCACCGCGCTCCTGACGGATGCGCCCTGCAAGCCCCTCCAGGCGAGAGCAG
GCCGGCCTGTGCTCAGTTTTGTAGCATCAAAACTAGGATTTCTCTTGTTACCCCCAGTCACTCCATTCAGTTTTCGTG
TCTTTCCCAGCTGCATCCATCCTTTCCTCATTTTCGTATGCAGCCGACTTTTTGTGACATCTTTGTATTCATTCTCTGC
AATTCAGCTGACCTGGCCAAGGAAACAAGATCCTAAGCGTCTTTCCGGCGGCGCCGTGGCTTAGTTGGTTAAAGC
GCCTGTCTAGTAAACAGGAGATCCTGGGTTCGAATCCCAGCGGTGCCTCCGTGTTTCCCCCACGCTTTTGCCAACAT
TAAACATTGTGAGGACAGTTGCAGAAACTCATAACTTCCATCCTACATGGTTTACTCACGTACCCATCTATCCTCTCC
CGGTGCATCTGCCACACGCTGTTGGGTTTTTGCTCTTCGTGCACATGGTACTTGCGCCTCGACCTGCAGTTACACCA
GTCGCATCATCTGTACAGCGCTAAAACCTAGCTGGGCGTGGTGGTCCTGCAGTCCCAGCTACTCGGGAGGCTGAG
GCAGGAGAATGGCTTGAATCCAGGAGGCGGAGGTGGCAGTGAGCCGAGATTGCGCCACTGCACTCCAGCCTGGT
GACAGAGCGAGACTCCATCTCAAAAAAAAAAAAAAAAAAAAAAGTCAGAATTAGGTACTAAAAGCCATATGACATG
CCTGAACAGGGACTTGAACCCTGGACCCTCAGATTAAAAGTCTGATGCTCTACCAACTGAGCTATCCAGGCTTCTT
CCCTGCTAGTTTATTTTAATGCAGTAATAAATAACAGCACTTTGTTAAAAATAATAAAGGTATAATCTGTGACACAT
CCAAAGTGGACAAGATGAAGAGATAATAGGTATACACCAGGATTCCCCTGGGCAAACTGGGACCTCTTGGTACCC
TATATATTAAGAGTCTCGGGTTTTGTTTTCACTTAAGCAAATGGTTAACGAATTAGCAGGTTAAGAAAAACTGTTTC
CCCGTAAGAAGCAGGGTTCTTTGGTGTTCAATGTGGAGCTCCGCCACTCCCAGCCCGGGTGAAGGAAAACTGGGA
AACAGAATGAATGTGATTATCTATTCGAAGATAAATTTCCACAAAGCATGCCGTTTGATAGTAGCTTATAATGTGG
AAGTAAGGCATCCTGTCATCCGGCCGGTTAGCTCAGTTGGTTAGAGCGTGGTGCTAATAACGCCAAGGTCGCGGG
TTCGATCCCCGTACTGGCCAAGTATTCTCTGTGGCTTTTATCACCAGAATGGATAGTAACCCAGACATCGATCTAAA
CGTGTACCTGTGTGTTTCTCCAGGCTTAACTTTGCCCCGAGAAAACGGATCTGTGAATTTGGTGCGCCCTCGCTTAC
TCGACAGCGGTTAATTTGAACGGGGACGTTTCTTTCCGCTGCCTCCAAGGCATACCCACATCCTACCACGATGGTG
GCGGCCGCAA

**Supplementary Figure 2**

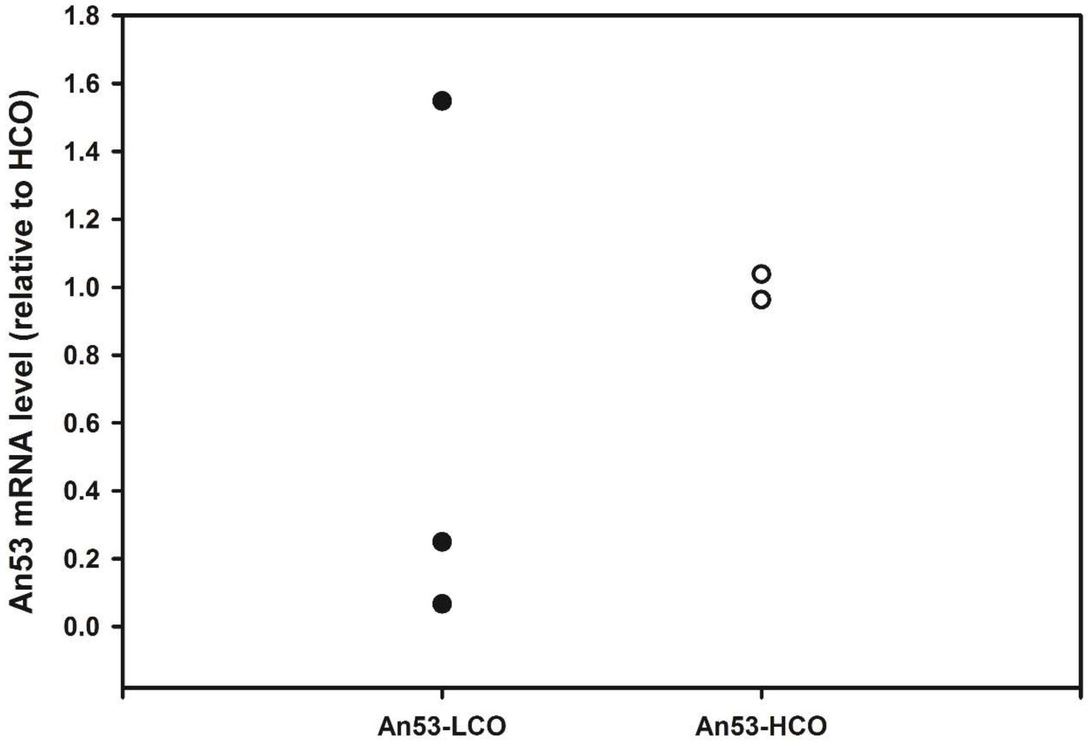


***In vivo* HCO and LCO An53 mRNA expression:** mRNA was extracted from the livers of mice dosed with AAV-HLP-An53-LCO and AAV-HLP-An53-HCO and relative levels of An53 mRNA was determined by quantitative PCR.
